# Mesenchymal Stem Cells-Derived Exosomes Alleviate Acute Lung Injury by Inhibiting Alveolar Macrophage Pyroptosis

**DOI:** 10.1093/stcltm/szad094

**Published:** 2024-02-13

**Authors:** Peipei Liu, Shengnan Yang, Xuecheng Shao, Chen Li, Zai Wang, Huaping Dai, Chen Wang

**Affiliations:** Department of Pulmonary and Critical Care Medicine, China-Japan Friendship Hospital, National Center for Respiratory Medicine, Institute of Respiratory Medicine, Chinese Academy of Medical Sciences, National Clinical Research Center for Respiratory Diseases, Beijing, People’s Republic of China; Chinese Academy of Medical Sciences, Peking Union Medical College, Beijing, People’s Republic of China; Department of Pulmonary and Critical Care Medicine, China-Japan Friendship Hospital, National Center for Respiratory Medicine, Institute of Respiratory Medicine, Chinese Academy of Medical Sciences, National Clinical Research Center for Respiratory Diseases, Beijing, People’s Republic of China; Harbin Medical University, Harbin, People’s Republic of China; Department of Obstetric Medicine, Tianjin Third Central Hospital, Tianjin, People’s Republic of China; Department of Pulmonary and Critical Care Medicine, China-Japan Friendship Hospital, National Center for Respiratory Medicine, Institute of Respiratory Medicine, Chinese Academy of Medical Sciences, National Clinical Research Center for Respiratory Diseases, Beijing, People’s Republic of China; China Capital Medical University, Beijing, People’s Republic of China; Institute of Clinical Medical Sciences, China-Japan Friendship Hospital, Beijing, People’s Republic of China; Department of Pulmonary and Critical Care Medicine, China-Japan Friendship Hospital, National Center for Respiratory Medicine, Institute of Respiratory Medicine, Chinese Academy of Medical Sciences, National Clinical Research Center for Respiratory Diseases, Beijing, People’s Republic of China; Chinese Academy of Medical Sciences, Peking Union Medical College, Beijing, People’s Republic of China; Department of Pulmonary and Critical Care Medicine, China-Japan Friendship Hospital, National Center for Respiratory Medicine, Institute of Respiratory Medicine, Chinese Academy of Medical Sciences, National Clinical Research Center for Respiratory Diseases, Beijing, People’s Republic of China; Chinese Academy of Medical Sciences, Peking Union Medical College, Beijing, People’s Republic of China; Harbin Medical University, Harbin, People’s Republic of China

**Keywords:** mesenchymal stem cells, exosomes, acute lung injury, pyroptosis, caspase-1

## Abstract

Acute lung injury (ALI) is an important pathological process of acute respiratory distress syndrome, yet there are limited therapies for its treatment. Mesenchymal stem cells-derived exosomes (MSCs-Exo) have been shown to be effective in suppressing inflammation. However, the effects of MSCs-Exo on ALI and the underlying mechanisms have not been well elucidated. Our data showed that MSCs-Exo, but not exosomes derived from MRC-5 cells (MRC-5-Exo), which are human fetal lung fibroblast cells, significantly improved chest imaging, histological observations, alveolocapillary membrane permeability, and reduced inflammatory response in ALI mice model. According to miRNA sequencing and proteomic analysis of MSCs-Exo and MRC-5-Exo, MSCs-Exo may inhibit pyroptosis by miRNAs targeting caspase-1-mediated pathway, and by proteins with immunoregulation functions. Taken together, our study demonstrated that MSCs-Exo were effective in treating ALI by inhibiting the pyroptosis of alveolar macrophages and reducing inflammation response. Its mechanism may be through pyroptosis-targeting miRNAs and immunoregulating proteins delivered by MSCs-Exo. Therefore, MSCs-Exo may be a new treatment option in the early stage of ALI.

## Graphical Abstract

**Graphical Abstract:**
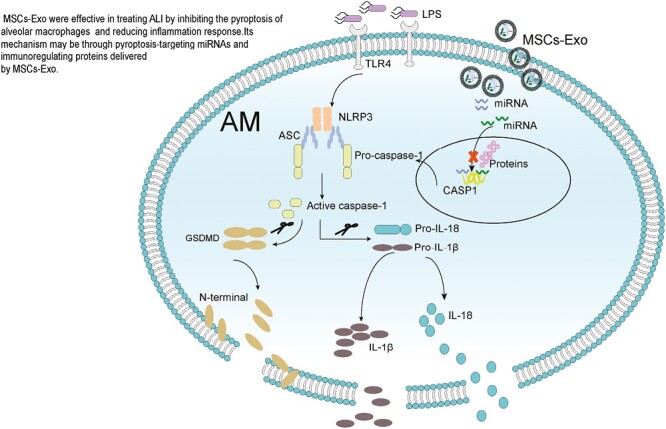

**Significance Statement**

Our study demonstrated that MSCs-Exo were effective in treating acute lung injury (ALI) by inhibiting the pyroptosis of alveolar macrophage and reducing inflammation response. Its mechanism may be through pyroptosis-targeting miRNAs and immunoregulating proteins delivered by MSCs-Exo. Therefore, MSCs-Exo may be a new treatment option in the early stage of ALI.

## Introduction

Acute respiratory distress syndrome (ARDS) is a disease characterized by diffuse pulmonary interstitial and alveolar edema. Its common risk factors may include pneumonia, non-pulmonary sepsis, aspiration of gastric contents, or non-cardiogenic shock.^1,2^ Acute lung injury (ALI) is an important pathological process of ARDS. Approximately 3 million patients worldwide are diagnosed with ARDS each year, with 10% of these patients admitted to intensive care units.^3^ Mechanical ventilation, with the use of supplemental oxygen and positive end-expiratory pressure, is the main treatment strategy for ARDS.^4^ However, it may lead to overdistension of the lung and increased transpulmonary pressure, leading to ventilator-induced or ventilator-associated lung injury by increasing epithelial injury, inflammation, and edema.^5^ Therefore, the mortality of ARDS remains high. An observational study on ARDS found that the in-hospital mortality was 34.9% for mild ARDS, 40.3% for moderate ARDS, and 46.1% for severe ARDS in the intensive care unit.^6^ Therefore, it is of great significance to explore new targets and effective treatments for ARDS.

Alveolar macrophages (AMs) are the first line of defense for the lung. It has been reported that the different forms of AM death, such as pyroptosis, autophagy, and necroptosis work in concert to induce lung inflammation.^7^ Among the 3 forms of death, pyroptosis can induce the secretion of a large amount of proinflammatory cytokines such as IL-1β and IL-18.^8^ Therefore, it is a form of cell death that may have crucial roles in the inflammatory response. Pyroptosis is a canonical caspase-1- or noncanonical caspase-11/4/5-mediated inflammatory cell death, whose occurrence depends on the activation of the Gasdermin protein family.^9^ Activation of inflammasomes by pathogen-associated molecular patterns or damage-associated molecular patterns causes caspase-1 activation. The activated caspase-1 causes the cleavage of GSDMD which results in the swelling of the cell and perforation of the cell membrane. In addition, activated caspase-1 induces cleavage of pro-IL-1β and pro-IL-18 to their mature forms of IL-1β and IL-18, respectively, which are subsequently released through the pores on the cell membranes.^10^ Studies have suggested that macrophage pyroptosis plays an important role in the inflammatory process in the lung. This inflammation promotes the accumulation of neutrophils in the lung, increases the levels of cytokines IL-6, IL-1β, and TNF-α in alveolar lavage fluid, and aggravates lung injury.^11^ Therefore, alveolar macrophage pyroptosis may be a new target for the treatment of ALI.

MSCs have the characteristics of immunomodulation, low immunogenicity, ease of in vitro culture, and promotion of tissue regeneration, making them an ideal choice for cell therapy.^12^ MSCs have been observed to promote tissue repair by reducing alveolar leakage, suppressing inflammation, and enhancing survival in animal models of ARDS induced by endotoxin.^13^ MSCs promote tissue repair mainly through paracrine actions by secreting exosomes, biologically active molecules, and microvesicles.^14^ Exosomes are microvesicles with diameters of 40–160 nm (100 nm on average) that originate from endosomes and are rich in DNA, RNA, and proteins.^15^ They are released into the extracellular space and participate in the physiopathological processes of the body. Due to the ability of MSCs-derived exosomes (MSCs-Exo) to inhibit inflammation and promote tissue repair, they have been used for the treatment of cardiovascular disease, kidney injury, traumatic brain injury, and several other diseases.^16-19^ Studies have expounded MSCs-Exo is a new therapeutic agent in rheumatoid arthritis, brain tumors, respiratory diseases, etc.^20-23^ The advantages of exosomes lie in the following aspects. For the whole cells, the increase in dosage may bring a risk of thrombosis.^24^ For the exosomes, this risk has been greatly reduced since the size of the exosomes is much smaller than the cells. In addition, exosomes can easily penetrate biological barriers, be modified in vitro, and are easier to store and administer.^25^ These qualities make them an emerging therapy for the treatment of many diseases. Studies found that MSCs-Exo can modulate macrophages by delivering miRNAs or proteins. In a hypoxia-induced pulmonary hypertension model, researchers demonstrated that MSCs-Exo regulated macrophage polarization not only by suppressing proinflammatory of TNF-α, Il6, and Ccl2, but by modulating anti-inflammatory of CD206, Arginase-1, and Retnla.^26^ According to proteomics analysis, MSC-Exo can attenuate inflammation by reshaping macrophage polarization by inhibiting the expression of TRAF1 and activating PI3K/AKT signaling pathway.^27^ Besides, miRNAs have been confirmed to have the function of regulating the macrophage, such as miR-21, miR-155, miR-125b, miR467b, miR-124, miR-142-5p, miR-146a, and miR-511.^28^ MSCs-Exo containing miR-21a-5p attenuated atherosclerosis by promoting macrophage M2 polarization, targeting KLF6 and ERK1/2 signaling pathways to reduce macrophage infiltration.^29^ The research revealed that MSCs were effective in improving cell survival and preventing pyroptosis in macrophages.^30^ However, the impact of MSC-Exo on alveolar macrophage (AM) pyroptosis in ALI remains not very clear. More evidence is required to ascertain the suitability of MSCs-Exo for the treatment of ALI and the underlying mechanisms for MSCs-Exo to regulate AM pyroptosis to alleviate ALI.

In this study, we aimed to investigate the efficacy of MSCs-Exo in alleviating lipopolysaccharide (LPS)-induced ALI and to explore the underlying mechanisms associated with alveolar macrophage pyroptosis.

## Materials and Methods

### Isolation and Characterization of Exosomes

MSCs were isolated from umbilical cord and expanded in culture according to the previously described protocol.^31^ Cells at second to 5 passages were used for further experiments. Umbilical cord tissues were obtained after normal delivery and after informed consent had been given by the patient or the family of the patient. We tested the cells in the third passage for stem cell properties. The cells were analyzed for stem cell surface markers using flow cytometry according to the instructions of the Human MSC Analysis Kit (BD, 562245). The cells were incubated with antibodies against MSC positive markers (CD90^+^, CD105^+^, CD73^+^) and negative cocktail markers (CD45, CD34, CD11b, CD19, HLA-DR) named as lineage cocktail (LIN-) before analysis using flow cytometry. Mesenchymal Stem Cell Adipogenesis Kit (Chemicon, SCR020), Mesenchymal Stem Cell Osteogenesis Kit (Chemicon, SCR028), and Mesenchymal Stem Cell chondrogenic Kit (Cyagen, HUXUC-90041) were used to determine the differentiation ability of the cells. In addition, the cells at the second passage were tested using karyotype analysis at our hospital.

MSCs or Medical Research Council cell strain 5 (MRC-5) cells which are human fetal lung fibroblast cells were cultured in an exosomes-free complete medium.^32,33^ Here, MRC-5 cells as the comparison object for it has been proven to be safe. The cell culture supernatant was collected to isolate exosomes. The supernatant was centrifuged at 300*g* for 10 minutes and at 2000*g* for 20 minutes at 4 °C to remove cell debris, filtered using a 0.22 μm filter, and centrifuged at 110 000 g for 90 minutes at 4 °C to get exosomes. The exosomes were washed with phosphate-buffered saline (PBS) and centrifuged at 110 000*g* for 90 minutes at 4 °C. The pellet was resuspended in 150 μL PBS and stored at −80 °C. The concentration of proteins in exosomes was measured using the BCA protein assay kit (Solarbio, PC0020). Exosomes were photographed using transmission electron microscopy and their size and quantity were analyzed using nanoparticle tracking analysis. Finally, the identity of exosomes was verified by assessing the expression of markers such as CD63, CD81, and Calnexin using Western blot.

### Cell Culture

MRC-5 cells were obtained from American Type Culture Collection and cultured in an exosomes-free complete medium at 37 °C with 5% CO_2_. J774A.1 murine macrophages were purchased from Beina Biology (Shanghai, China) and cultured in DMEM medium (BI, 06-1055-57-1ACS-1) supplemented with 10% FBS. Alveolar macrophages were isolated according to the previously described protocol.^34^

### Internalization of Exosomes

MSCs-Exo were labeled with PKH26 Red Fluorescent Cell Linker Kits (Sigma) according to the instructions of the manufacturer. AMs were seeded in an 8-well chamber slide and incubated with a complete medium overnight. Thereafter, the medium was replaced with an FBS-free medium, and the cells were incubated with PKH26-labeled exosomes for 4 hours at 37 °C with 5% CO_2_. The slide was then washed with PBS, fixed with paraformaldehyde, and washed with PBS again. The cells were permeabilized using 0.2% Triton X-100 for 10 minutes, incubated with phalloidin (YEASEN,40736ES75) in the dark for 30 minutes, washed with PBS, mounted with mounting medium containing DAPI, and imaged using confocal microscopy (Zeiss).

### Development of the ALI Model and Collection of Samples

Ten to 12-weeks-old C57BL/6 male mice were purchased from Vital River Laboratory Animal Technology (Beijing, China). The mice were fed with the standard diet and housed under SPF conditions at temperatures ranging from 23 °C to 25 °C, with 60%-70% humidity and 12/12 h dark-light cycle. Mice were anesthetized using isoflurane. The ALI model was induced through intratracheal instillation of 10 mg/kg LPS in 50 μL PBS (Sigma, L2630). The control mice were administered with equal volumes of saline. The mice were divided into 4 treatment groups (16 mice per group): control group, LPS group, MSCs-Exo therapy group, and MRC-5-Exo therapy group. Intratracheal instillation of 200 µg Exo was performed in mice 4 hours after ALI was induced using LPS, and the mice were euthanized 24 hours later. The blood and tissue samples of 3 mice per group were collected for histological staining and protein analysis, BALF of 5 mice per group was collected for analyzing macrophages using flow cytometry and supernatants by ELISA, while lung tissues of 5 mice per group were isolated for analyzing lung wet-to-dry weight ratio. In addition, 3 mice per group were sacrificed after 48 hours and analyzed using Micro-CT.

### Histological Staining, Lung Injury Score and Micro-CT

The left lung tissues were fixed in paraformaldehyde, dehydrated, paraffin-embedded, sectioned, and stained with H&E. The criteria for assessing lung injury score was based on the official American Thoracic Society workshop report.^35^ On the other hand, mice were anesthetized using isoflurane and scanned using a micro-CT according to the instructions of the manufacturer.

### Cell Pyroptosis Model and Live-Cell Imaging

AM was seeded in 35 mm glass-bottom dishes (MatTek, P35G-0-10-C). For the pyroptosis model, the AM was cultured in an FBS-free medium and primed with 50 ng/mL LPS for 2 hours. Thereafter, the culture medium was replaced with an FBS-free medium containing 10 µM Nig and the cells were cultured at 37 °C with 5% CO_2_. Cell images were captured using the Olympus IX71 at a time interval of 5 minutes per image until moderate time. For the treatment group, the culture medium was replaced with FBS-free medium containing 10 µM Nig and MSCs-Exo, and the other procedures carried out as in the pyroptosis model group. For the treatment with MSCs-Exo, cells were divided into 4 groups: control group, LPS/ Nig group, MSCs-Exo therapy group, and Ac-YVAD-cmk (YVAD) therapy group. Cells were imaged using a microscope or collected for analysis using WB and flow cytometry at the appropriate time points.

### Analysis of BALF, Serum, and Cell supernatants

The total protein content of BALF supernatants was analyzed using the BCA protein assay kit (Solarbio, PC0020). BALF, serum, and cell supernatants were analyzed for Interleukin-1β (IL-1β, ExcellBio, EM001-96) and interleukin-18 (IL-18, Cloud-Clone Corp, SEA064Mu) using ELISA. CytoTox 96 Non-Radioactive Cytotoxicity Assay (Promega, G1780) was used to evaluate lactate dehydrogenase (LDH) activity in BALF and cell supernatants.

### Propidium iodide, immunofluorescence staining, and flow cytometric analyses

AMs or J774A.1 cells were seeded in 24-well plates, treated with the indicated reagents, and harvested after 12 hours or 24 hours. The plates were incubated with PI staining (Immunochemistry, 98) in the dark for 5 minutes and observed using a fluorescence microscope. For immunofluorescence analysis, cells were fixed with paraformaldehyde, permeabilized with 0.2% Triton X-100, blocked with animal-free blocking solution, and incubated with caspase-1 p20 (Santa Cruz, sc-398715) at 4 °C overnight. Afterward, the cells were washed with PBS, incubated with Alexa Fluor-conjugated secondary antibodies, and mounted using a mounting medium containing DAPI.

For flow cytometric analyses, cells were harvested as described before, blocked with TruStain fcX, incubated with CD11c (Biolegend, 117307), and CD170 (Biolegend, 155507) for 20 minutes. Thereafter, the cells were washed with cell staining buffer (Biolegend, 420201), centrifuged at 300*g* for 5 minutes, resuspended in cell staining buffer, and analyzed using flow cytometry. Caspase-1 activity was analyzed by treating the cells with FAM-FLICA Caspase Assays (Immunochemistry, 98) according to the instructions of the manufacturer, followed by analysis using flow cytometry.

### Western Blot

Cell supernatants were centrifuged in a 10 kDa ultrafiltration filter (Millipore, UFC801096), and lysed using a loading buffer. The cells and mice lung tissues were also lysed with loading buffer and all samples were denatured. The moderate proteins were then separated in 10% or 12% BeyoGel Plus PAGE gels (Beyotime, P0455S or P0458M) and transferred to polyvinylidene fluoride membranes (PVDF, Millipore, SLGVV255F). The membranes were then blocked with 5% skim milk in TBST, and incubated with primary antibodies: NLRP3 (CST, 15101), Cleaved-IL-1β (CST, 63124), IL-1β (CST, 31202), Cleaved Caspase-1 (CST, 89332&SantaCruz, SC-398715), Caspase-1 (Santa Cruz, SC-398715 & Proteintech, 22915-1-AP), IL-18 (Proteintech, 60070-1-Ig), GSDMD & Cleaved Gasdermin D (Abcam, ab209845), and GAPDH (Proteintech, 60004-1-Ig). Subsequently, the membranes were incubated with secondary antibodies, visualized using an electrochemiluminescence (ECL) kit (Millipore, WBKLS0100), and photographed using the Bio-Rad imaging system (Bio-Rad).

### MiRNA Sequencing of Exosomes

For miRNA sequencing, total RNA was extracted from exosomes using the miRNeasy Micro Kit (Qiagen, 217084). RNA quality was assessed using the Agilent Bioanalyzer 4200 (Agilent Technologies). Then, we generated sequencing libraries using the QIAseq miRNA Library Kit (Qiagen) for Illumina and performed on the Illumina Novaseq platform to sequencing (Illumina). The experiments and data analysis were offered by Wayen Biotechnologies (Shanghai, China). We used Ingenuity pathway analysis (IPA) software, which was performed by the Proteomics Platform of Core Facility of Basic Medical Sciences, Shanghai Jiao Tong University School of Medicine to further analyze part of the miRNA sequencing data. Besides, we used the Cytoscape 3.8.2 software to visualize part of the miRNA sequencing data.^36^ The data can be retrieved through GSE209966.

### Proteomic Analysis of Exosomes

The proteomic analysis of exosomes was performed using a label-free analysis technology. Protein was extracted from exosomes, quantified with BCA assay, digested by trypsin, and dried by centrifugal concentration. Then, the peptides were desalted using MonoSpin C18 desalting column (GL Sciences Inc., Japan, 5010-21701), analyzed by Orbitrap Fusion Lumos tandem mass spectrometry (Thermo Fisher Scientific) coupled to an EASY-nLC 1200 liquid chromatography system (Thermo Fisher Scientific). Protein identification and quantification were performed using Proteome Discoverer 2.4 (Database: Swissprot; taxonomy: Homo sapiens) with the default setting. The experiments and data analysis were offered by Wayen Biotechnologies (Shanghai, China). We used the Metascape platform online analytical tool to further analyze part of the proteomic analysis data.^37^ The mass spectrometry proteomics data have been deposited to the ProteomeXchange Consortium via the PRIDE^38^ partner repository with the dataset identifier PXD033839.

### Statistical Analysis

All data are presented as the mean ±  SD. All results were analyzed using the GraphPad Prism 8.0 statistical software. The means of 2 groups were compared using Student’s t test, while multiple groups were compared using non-parametric one-way ANOVA. Differences were considered to be statistically significant when the *P* value was <.05.

## Results

### The Characteristics of MSCs-Exo

Characterization of cells extracted from the umbilical cord showed that the cells maintained fibroblast-like morphology (Supplementary Fig. S1A) and possessed normal diploid karyotypes (Supplementary Fig. S1B). The cells also showed the ability to differentiate into the adipogenic, osteogenic, and chondrogenic lineages compared to controls, and expressed positive MSCs markers CD105, CD73, and CD90 (Supplementary Fig. S1C and S1D). These results indicated that the isolated cells were MSCs. We further assessed the presence of extracellular vesicles derived from MSCs, as well as from MRC-5 cells which are human embryonic lung fibroblast cells. The MRC-5 cell line was first isolated in 1966 from the lung tissue of a 14-week fetus, researchers repeated testing it found that the cells have characteristics as follows: stable diploid karyotype, no effect for invasive nodule in vivo, and no expression of HL-A7 antigen.^32,33^ Since MRC-5 cells have been proven to be safe and is currently widely used in the field of vaccines,^39^ and as a fibroblast cell, it might play a role in the fibrotic repair of lung injury, we chose to use exosomes derived from MRC-5 cells as the comparison object. From our analysis, we identified extracellular vesicles from both cell types. We observed round vesicles with a mean diameter of 100 nm (Supplementary Fig. S2A and S2B). The vesicles expressed common exosomal markers such as CD63 and CD81 but did not express Calnexin when compared to the cell lysates (Supplementary Fig. S2C). These results suggested that the extracellular vesicles we isolated were exosomes.

### Therapeutic Effects of MSCs-Exo on ALI

**Figure 1.**
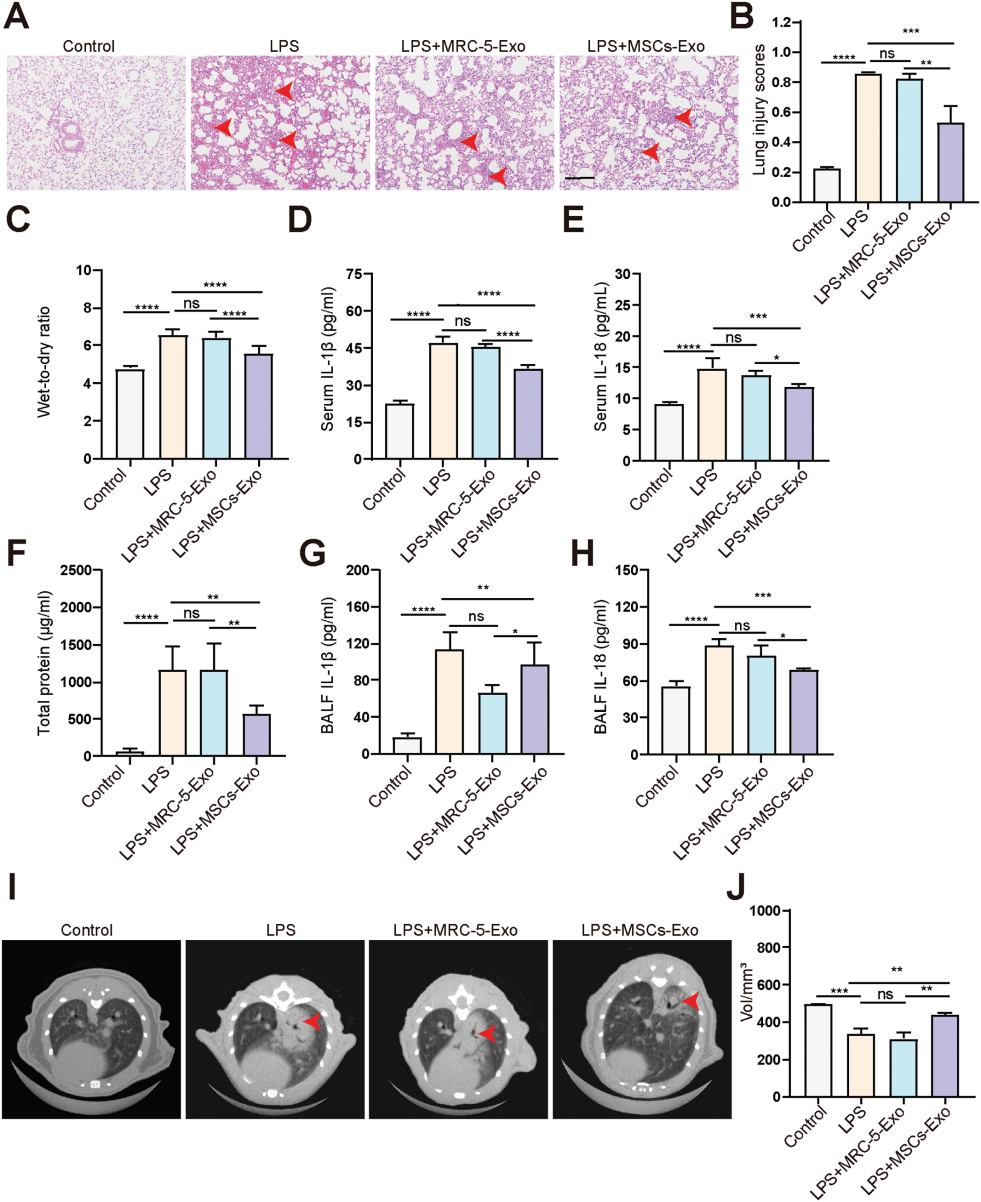
MSCs-Exo alleviate LPS-induced acute lung injury (*n* = 16/group). (**A**) H&E staining for the lung tissues. Lung lesions indicated by red arrowhead. Scale bar = 50 µm. (**B**) Quantification scores of lung injury based on histological analysis. (**C**) Analysis of lung wet-to-dry weight ratio in mice. (**D&E**) Analysis of IL-1β (D) and IL-18 (**E**) levels in serum using ELISA. (**F**) Total protein levels in BALF. (**G&H**) Analysis of IL-1β (G) and IL-18 (H) levels in BALF using ELISA. (I) Micro-CT horizontal images of mice. Lung lesions indicated by arrowhead. (J) Quantification of lung volume. ****P < .0001, ***P < .001, **P < .01, *P < .05, ns: not significant. Data are presented as the mean ±  SD.

This assay was carried out according to the standard of animal experimental acute lung injury recommended by the American Thoracic Association,^22^ We first established an ALI mice model by administering LPS to mice. We then evaluated the effects of MSCs-Exo in the ALI mice model. The histopathologic manifestations of LPS-induced ALI mice model included diffuse alveolar injury, lung tissue structure destruction, alveolar septal thickening, inflammatory cell infiltration, a little pink fibrinous deposit, increased levels of IL-1β and IL-18 in serum and bronchoalveolar lavage fluid (BALF), and increased secretion of LDH and total protein in BALF (*P* < .05) (Fig. 1A–1H). Treatment with MSCs-Exo alleviated LPS-induced lung injury, reduced the level of IL-1β and IL-18 in serum and BALF, and reduced the lung wet-to-dry ratio and total protein in BALF (*P* < .05; Fig. 1A–1H). However, MRC-5-Exo treatment had no effect on the mice. Interestingly, MSCs-Exo treatment alleviated LPS-induced ALI in a dose-dependent manner, with high-dose MSCs-Exo being more effective than medium- and low-dose MSCs-Exo (*P* < .05; Supplementary Fig. S2D and S2E). We also used Micro CT and quantification of lung volume to evaluate LPS-induced mice lung injury after 48 hours. The CT showed that there were diffuse and part-solid lesions in the LPS group. MSCs-Exo treatment alleviated LPS-induced exudative and patchy lesions, while MRC-5-Exo treatment had no effect (*P* < .01; Fig. 1I and 1J). These results suggested that MSCs-Exo had therapeutic effects on ALI.

### MSCs-Exo Alleviate LPS-Induced Inflammation by Inhibiting AM Pyroptosis

**Figure 2.**
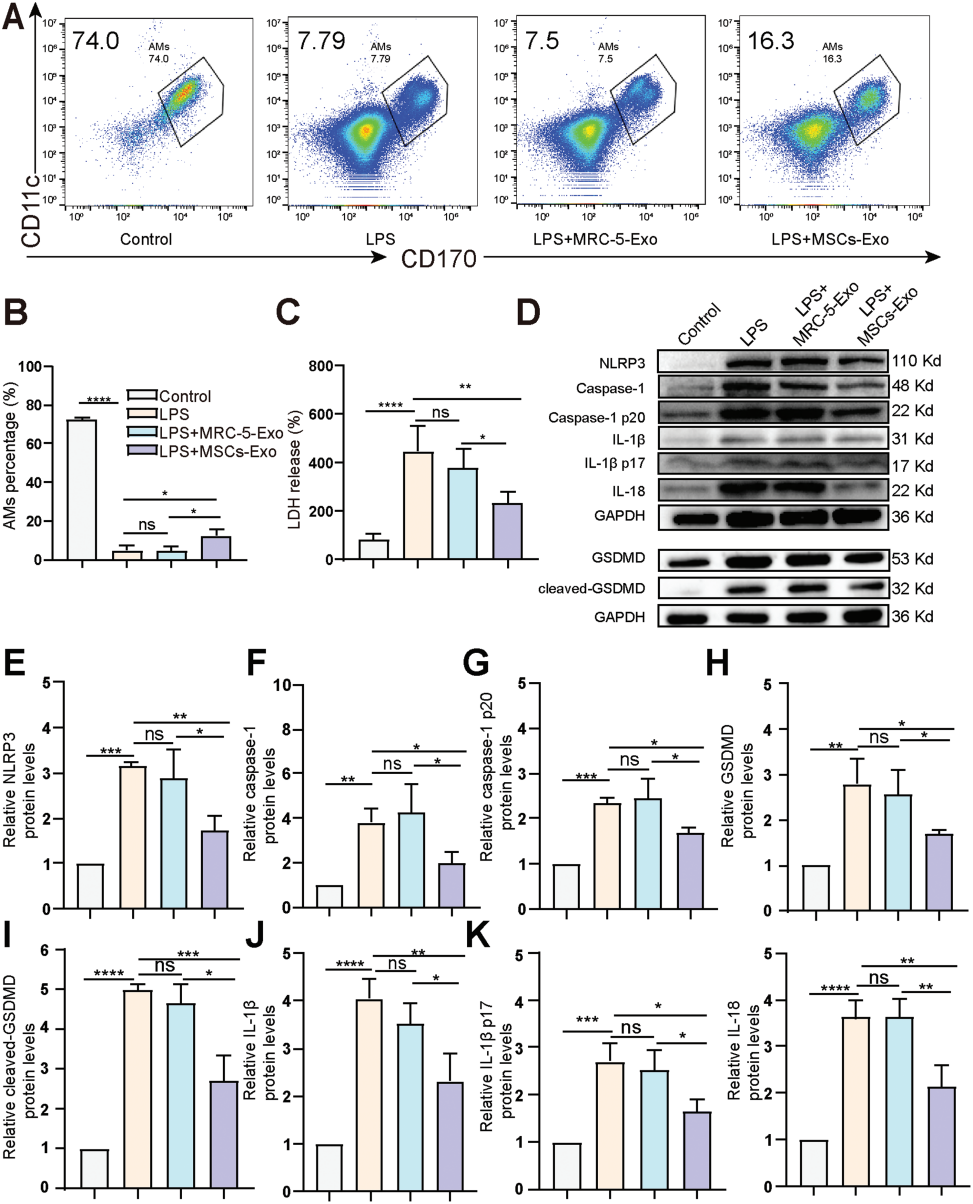
MSCs-Exo inhibits pyroptosis in mice. (**A**) Flow cytometry analysis to identify the proportion of AM in BALF. AM is CD170^+^, and CD11c^+^ cells. (**B**) The proportion of AM in BALF. (**C**) Relative LDH release in BALF. (**D**) Expression analysis of NLRP3, caspase-1, caspase-1 p20, GSDMD, cleaved-GSDMD, IL-1β, IL-1β p17, and IL-18 in lung tissues using Western blot. (**E-L**) Quantification of protein levels of NLRP3 (E), caspase-1 (F), caspase-1 p20 (G), GSDMD (H), cleaved-GSDMD (I), IL-1β (J), IL-1β p17 (K) and IL-18 (L). *n* = 3/group. ****P < .0001, ***P < .001, **P < .01, *P < .05, ns: not significant. Data are presented as the mean ± SD.

Given that MSCs-Exo have anti-inflammatory effects and AM pyroptosis promotes inflammatory reactions in the development of ALI, we proposed that MSCs-Exo regulate AM pyroptosis. We evaluated the effects of MSCs-Exo on AM pyroptosis by determining the proportion of AM in BALF in each group of mice. The results showed that LPS treatment reduced the proportion of AM in BALF, but treatment with MSCs-Exo was able to reverse this effect (*P* < .05; Fig. 2A and 2B). We further found that MSCs-Exo inhibited LPS-induced secretion of LDH in BALF (*P* < .01; Fig. 2C). We also examined caspase-1-mediated pyroptosis in LPS-induced ALI. The results showed LPS treatment increased the expression of NLRP3, caspase-1, GSDMD, IL-1β, and IL-18 protein in the lung tissue, as well as the expression of cleaved caspase-1, GSDMD, and IL-1β protein (*P* < .05). MSCs-Exo treatment decreased the expression of NLRP3, caspase-1, GSDMD, IL-1β, IL-18, caspase-1 p20, cleaved-GSDMD, and IL-1β p17 caused by LPS (*P* < .05), while MRC-5-Exo treatment had no effect (Fig. 2D–2L). These results suggested that AM may undergo pyroptosis after lung injury and produce pro-inflammatory factors that promote the pathogenesis of ALI and that MSCs-Exo may alleviate LPS-induced inflammation by inhibiting AM pyroptosis.

### MSCs-Exo Inhibit AM Pyroptosis by Targeting Activated Caspase-1

**Figure 3.**
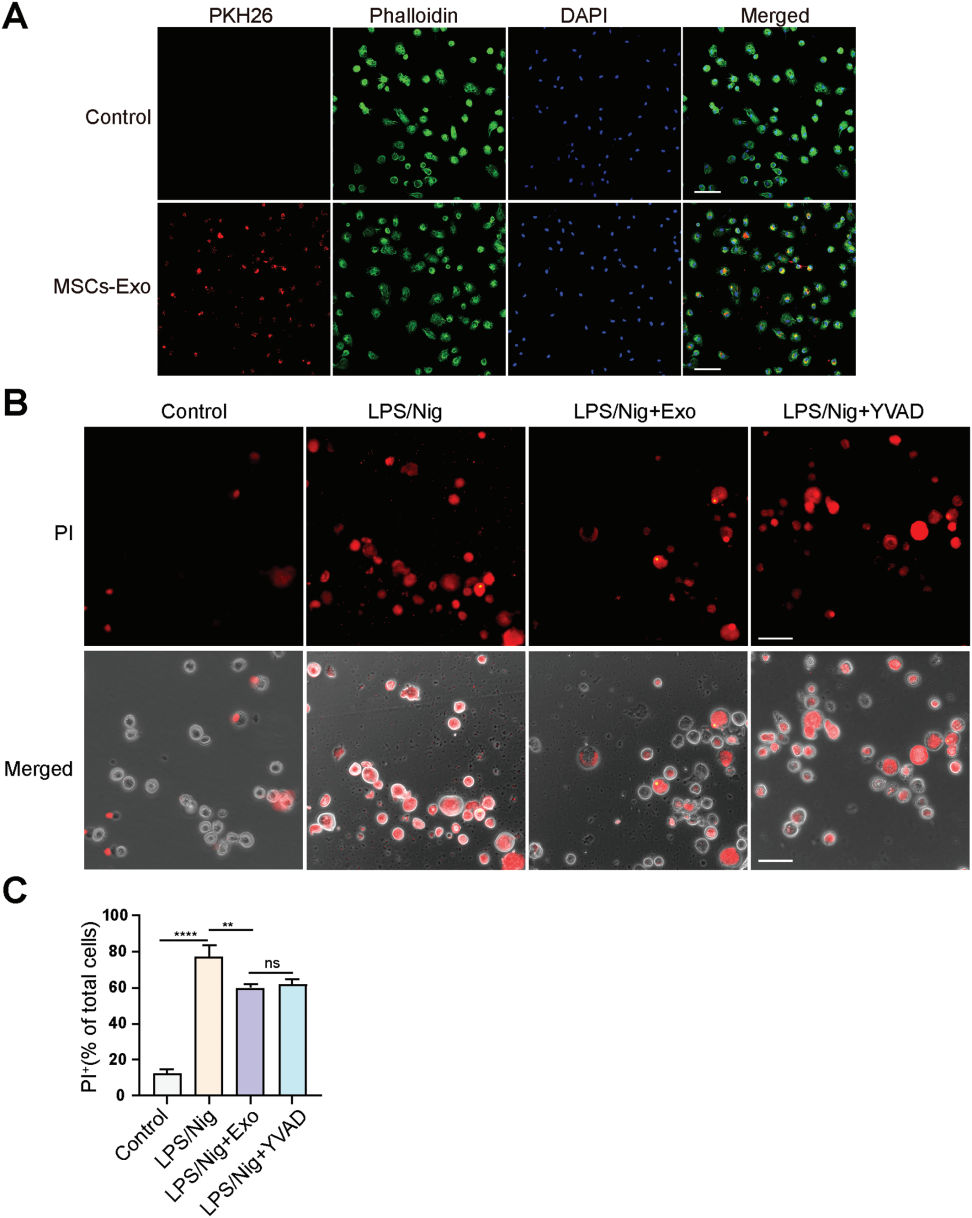
MSCs-Exo inhibits AM pyroptosis. (**A**) Confocal microscopy analysis of internalization of MSCs-Exo by AM. MSCs-Exo were labelled with PKH26 , AM was stained with phalloidin, nuclear was stained with DAPI . Scale bar = 50 µm. (**B**) AM were primed with LPS for 2 h, treated with Nig, and subsequently treated with Exo or YVAD. PI staining of AM 12 h after treatment. PI (red) images were merged with bright-field images. Scale bar = 100 µm. (**C**) Quantification of the percentage of PI^+^ AM. ****P < .0001, **P < .01, ns: not significant. Data are presented as the mean ± SD. Exo: SCs-Exo. YVAD: Ac-YVAD-cmk. Nig: Nigericin.

To further prove the role of MSCs-Exo in AM pyroptosis, we used primary AM to address this issue. We isolated AM from BALF and analyzed the expression of CD170 and CD11c, which are cell surface markers of AM. The results showed that the purity of AM in the isolated cells was over 90% (Supplementary Fig. S3A). We then labeled MSCs-Exo with PKH26 and incubated them with AM. The data showed that MSCs-Exo could be internalized by AM (Fig. 3A). Nigericin (Nig) induced pyroptosis in LPS-primed AM, by causing the morphology of cells to change to spherical and the cell membranes to swell and rupture (Supplementary Fig. S3B and S3C and Supplementary Video S1). MSCs-Exo increased the time taken for the LPS/Nig-induced swelling and rupture of AM to occur (Supplementary Video S2). We further assessed the effects of MSCs-Exo on AM pyroptosis. A caspase-1 inhibitor, YVAD, was used as the control.^40^ Treatment with MSCs-Exo and YVAD for 12 hours suppressed AM pyroptosis, MSCs-Exo reduced the number of PI-positive cells induced by LPS/Nig (*P* < .01) (Fig. 3B and 3C). This provides initial evidence that MSCs-Exo inhibits AM pyroptosis.

**Figure 4.**
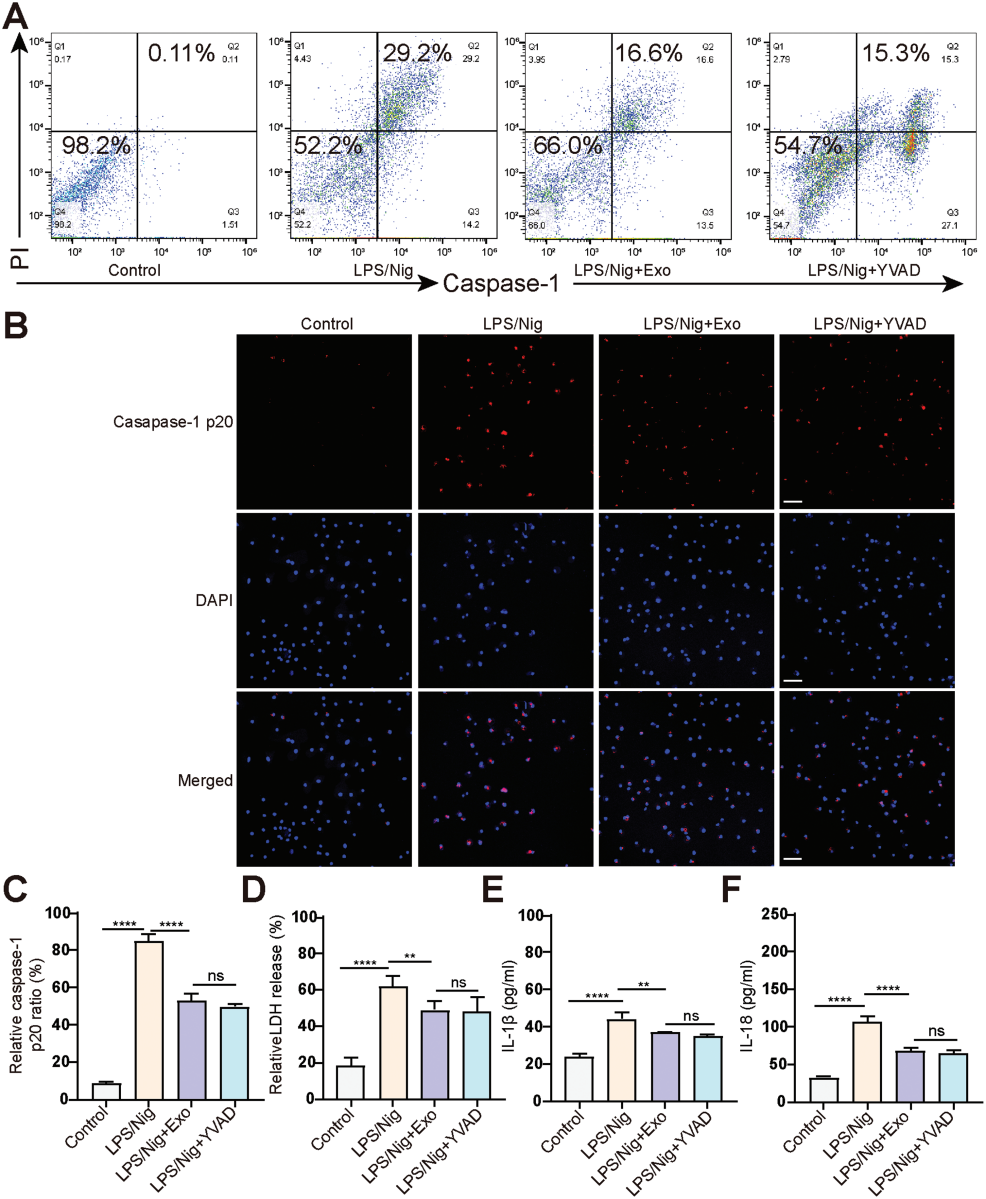
`MSCs-Exo inhibits the activation of caspase-1 during AM pyroptosis. (**A**) Flow cytometry analysis to identify the percentage of PI^+^ and active caspase-1^+^ AM. (**B**) Immunofluorescence staining for caspase-1 p20 in AM. Cells were immunostained for caspase-1 p20 , while nuclear was stained with DAPI . Scale bar = 50 µm. (**C**) Quantification of the percentage of caspase-1 p20^+^ cells. (**D**) Relative LDH release in supernatant of AM. (**E&F**) Analysis of the IL-1β (E) and IL-18 (F) levels in supernatant of AM using ELISA. ****P < .0001, **P < .01, ns: not significant.

Since caspase-1 activation is critical for pyroptosis to occur, we further investigated the effect of MSCs-Exo on caspase-1 activation. The FAM-FLICA caspase activation assay results showed that LPS/Nig treatment caused caspase-1 activation, while MSCs-Exo treatment inhibited caspase-1 activation in AM (Fig. 4A). In addition, we evaluated the levels of the cleaved caspase-1 (caspase-1 p20) in AM and found that MSCs-Exo also reduced the ratio of caspase-1 p20 activated by LPS/Nig (*P* < .0001; Fig. 4B and 4C). The measurement of LDH, IL-1β, and IL-18 levels in the culture supernatant indicated that MSCs-Exo reduced LDH, IL-1β, and IL-18 release in AM (*P* < .01; Fig. 4D–4F).

**Figure 5.**
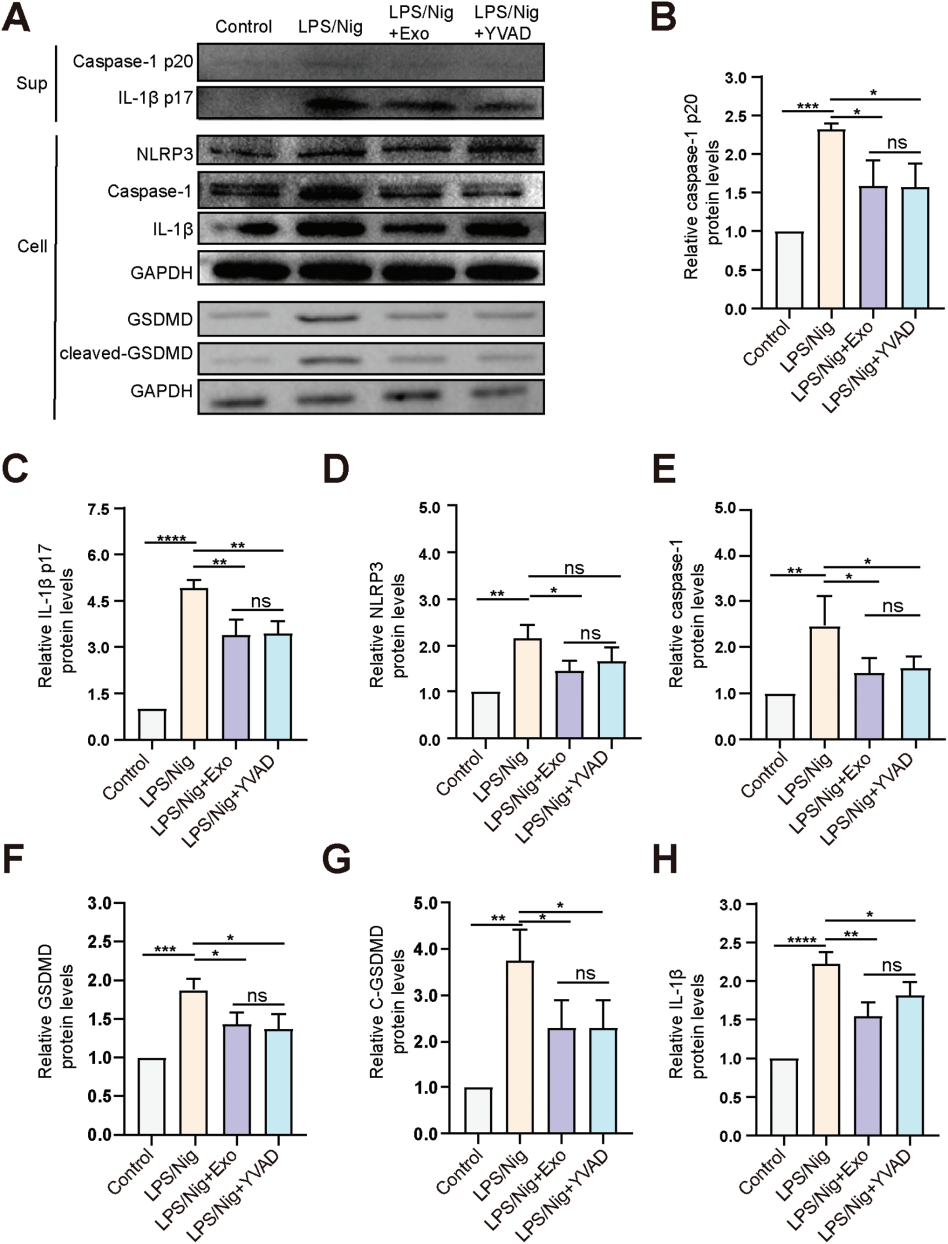
MSCs-Exo inhibits the activation of caspase-1 and pyroptosis of J774A.1 cells. (**A**) Expression analysis of caspase-1 p20 and IL-1β p17 in supernatant of J774A.1 cells, and NLRP3, caspase-1, GSDMD, cleaved-GSDMD, and IL-1β in J774A.1 cell lysates using Western blot. (**B–H**) Quantification of protein levels of caspase-1 p20 (B), IL-1β p17 (C), NLRP3 (D), caspase-1 (E), GSDMD (F), cleaved-GSDMD (G), and IL-1β (H). *n* = 3/group, ****P < .0001, ***P < .001, **P < .01, *P < .05, ns: not significant. Data are presented as the mean ± SD.

Since AM is difficult to expand in vitro and does not meet WB experimental needs, we used J774A.1 macrophage cell line to verify the inhibitory effect of MSCs-Exo on the cell pyroptosis. The results of J774A.1 were consistent with AMs. MSCs-Exo could be internalized by J774A.1. Treatment of MSCs-Exo delayed the swelling and rupture of J774A.1 caused by LPS/Nig, reduced the number of PI-positive cells and the release of LDH, IL-1β, and IL-18 induced by LPS/Nig, and inhibited caspase-1 activation in J774A.1 (*P* < .05; Fig. S4). Besides, MSCs-Exo also reduced the ratio of caspase-1 p20 activated by LPS/Nig in J774A.1 (*P* < .0001) (Supplementary Fig. S5). We first evaluated the expression of pyroptosis-related proteins in J774A.1 cells. LPS/Nig treatment not only increased the levels of NLRP3, Caspase-1, GSDMD, IL-1β, and cleaved-GSDMD in cell lysates but it also increased Caspase-1 p20 and IL-1β p17 levels in cell-culture supernatants (*P* < .001). MSCs-Exo reduced the elevated levels of pyroptosis-related proteins induced by LPS, as well as cleaved Caspase-1 (*P* < .05; Fig. 5). These results suggested that MSCs-Exo inhibited J774A.1 pyroptosis by inhibiting the activation of caspase-1.

### Multiomics Analysis Reveals the Possible Mechanism of MSCs-Exo Inhibiting Pyroptosis

**Figure 6.**
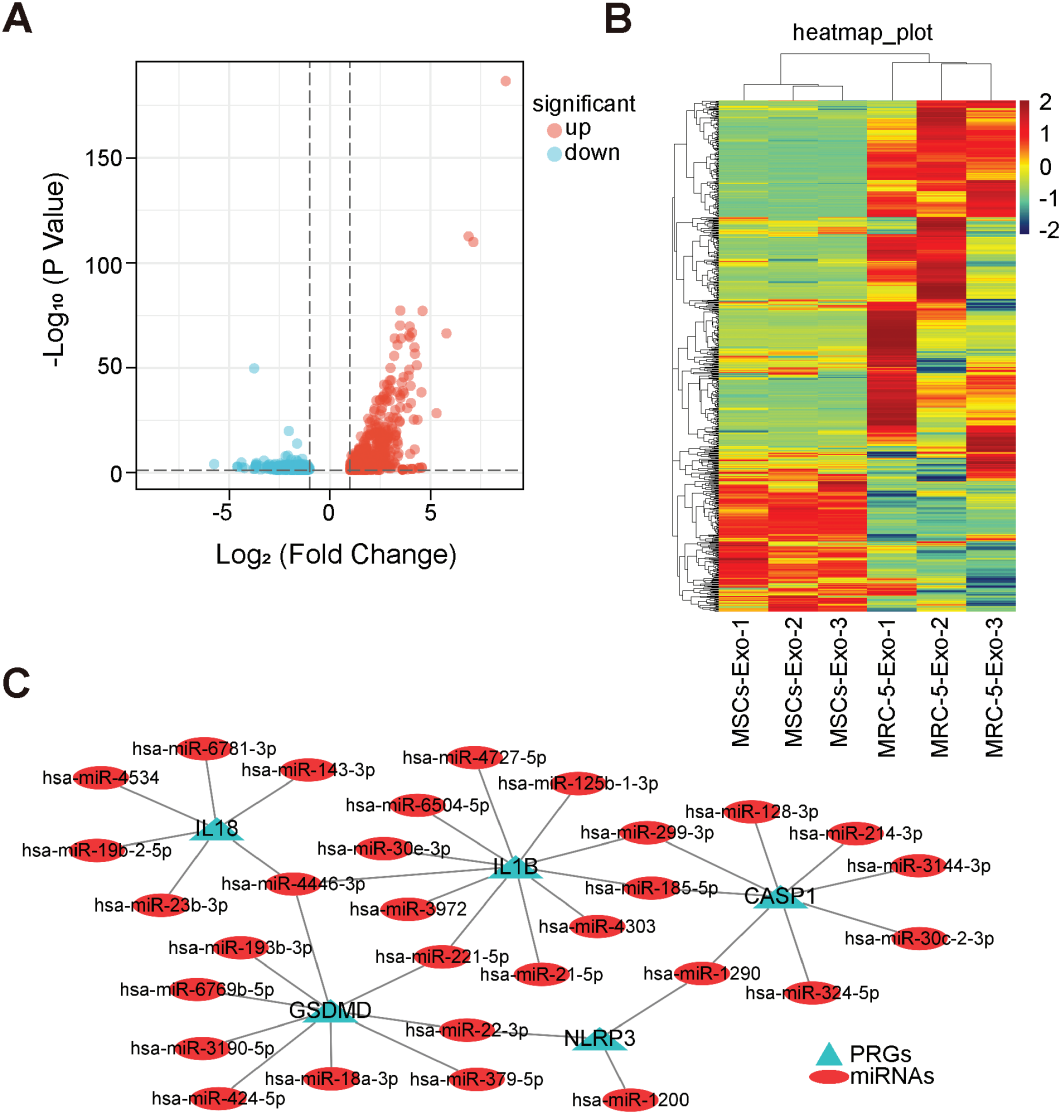
MiRNAs sequencing analysis of MSCs-Exo and MRC-5-Exo. (**A**) Volcano map illustrating differentially expressed miRNAs in MSCs-Exo and MRC-5-Exo. Absolute log2 fold change > 1. *P*-value < .05. Dots indicate upregulated and downregulated miRNAs (*n* = 3). (**B**) Heatmap illustrating hierarchical cluster analysis of differentially expressed miRNAs between MSCs-Exo and MRC-5-Exo. (**C**) The network of upregulated miRNAs and pyroptosis-related genes (NLRP3, CASP1, GSDMD, IL1B, and IL18 genes) interactions. Ellipses represent upregulated miRNAs. Triangles represent pyroptosis-related genes.

As previous studies have found that exosomes could regulate the biological functions of the target cells by delivering miRNAs and proteins, we compared the differential miRNAs and proteins between MSCs-Exo and MRC-5-Exo. MiRNA sequencing revealed that a total of 710 miRNAs were differentially expressed in MSCs-Exo compared with MRC-5-Exo, including 398 upregulated and 312 downregulated miRNAs (absolute log2FoldChange > 1; *P*-value < 0.05; Fig. 6A Supplementary Table S1). The above 710 miRNAs were also analyzed by hierarchical clustering (Supplementary Fig. S6B). To further explore the mechanism of miRNAs in MSCs-Exo-inhibited pyroptosis, a total of 86 pyroptosis-related genes (PRGs) were collected from the IPA (Supplementary Table S2). We obtained interactions between upregulated miRNA in MSCs-Exo and PRGs using the TargetScan database.^41^ There were 175 upregulated miRNAs targeting 82 PRGs (Supplementary Table S3). Among them, 30 upregulated miRNAs could target NLRP3, CASP1, GSDMD, IL1B, and IL18 genes (Fig. 6C), which are key factors in the caspase-1-mediated pyroptosis pathway.

**Figure 7.**
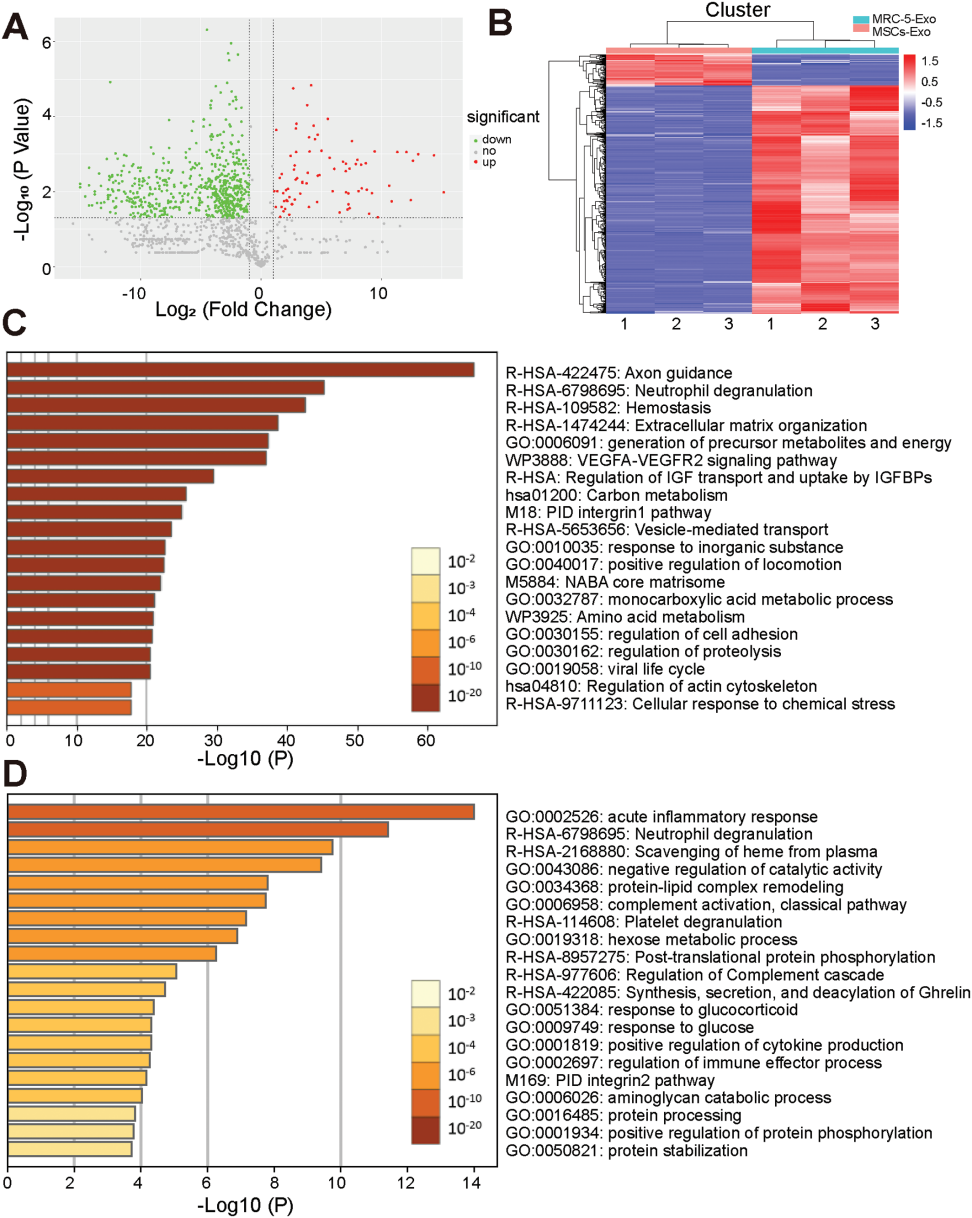
Proteomic analysis of MSCs-Exo and MRC-5-Exo. (**A**) Volcano map illustrating differentially expressed proteins in MSCs-Exo and MRC-5-Exo. Fold change ≥ 2 or ≤ 0.5. *P*-value < .05. Dots indicate upregulated, downregulated and no change proteins (*n* = 3). (**B**) Heatmap illustrating hierarchical cluster analysis of differentially expressed proteins between MSCs-Exo and MRC-5-Exo. (**C**) Pathway and process enrichment analysis of proteins with lower level in MSCs-Exo compared with MRC-5-Exo. Log10(*P*) is the *P*-value in log base 10. (**D**) Pathway and process enrichment analysis of proteins with higher level in MSCs-Exo compared with MRC-5-Exo. Log10(*P*) is the *P*-value in log base 10.

Proteomics analysis identified a total of 693 differentially expressed proteins, including 83 upregulated and 610 downregulated proteins (fold change ≥ 2 or ≤ 0.5; *P*-value < .05; Fig. 7A; Supplementary Table S4). The above 693 proteins were also analyzed by hierarchical clustering (Fig. 7B). Further enrichment analysis of 610 proteins with higher levels in MRC-5-Exo using the Metascape platform revealed that these proteins, including many extracellular matrix proteins and ribosomal proteins, may be involved in multiple pathways and processes, including metabolism, locomotion, and biological adhesion. The top 20 clusters are shown in Fig. 7C; Supplementary Table S5 (*P*-value < .01, a minimum count of 3, and an enrichment factor > 1.5). Further, we also enriched analysis of 83 upregulated proteins in MSCs-Exo (Fig. 7D; Supplementary Table S6). Interestingly, these proteins are mainly immunoregulating proteins, that are involved in the processes of response to stimulus, detoxification, and biological regulation, indicating that MSCs-Exo may inhibit AM pyroptosis through immunoregulation.

In summary, our study showed that the pyroptosis of AM plays a crucial role in the pathogenesis of ALI, and that MSCs-Exo may ameliorate ALI by inhibiting AM pyroptosis through delivered miRNAs and proteins (Supplementary Fig. S6).

## Discussion

Currently, MSCs are widely used in basic and clinical research. They have been shown to reduce the inflammatory response, promote lung tissue regeneration, and improve the outcome of patients in coronavirus-induced lung injury.^42^ Extracellular vesicles (EVs) are particles naturally released from cells. According to physical characteristics of size, EV includes small EVs (<100 nm or < 200 nm) and medium/large EVs (>200 nm).^43^ Exosomes was one kind of the small EVs. MSCs-derived exosomes are effective in the therapy of various diseases in research, such as brain, lung, liver, and cardiovascular diseases.^20-23,44-47^ It has been reported that prophylactically injection of MSCs-exo had a therapeutic effect on traumatic ALI.^48^ However, more evidence is required to show that MSCs-Exo is suitable for treating ALI. Our current study demonstrated that MSCs-Exo could alleviate acute lung injury by inhibiting alveolar macrophage pyroptosis.

The vesicles in our experiment were approximately 100 nm diameter and expressed exosomal markers such as CD63 and CD81. Therefore, they were identified as exosomes. Although the administration of exosomes in advance may enable them to take effect earlier and achieve better results, we chose to evaluate the effect of therapeutic drug administration to get closer to clinical application scenarios. We applied MSCs-Exo by intratracheal instillation^49^ in ALI mice model and found that MSCs-Exo alleviated histological severity, protein permeability, and inflammatory response induced by LPS. Our findings were consistent with findings from other studies, showing that MSCs-Exo has the capacity for tissue repair. We also observed that the efficacy of MSCs-Exo was dose-dependent, with the high-dose being more effective than the middle dose. These data indicated that MSCs-Exo has therapeutic potential for treating ALI. Our results were in line with results from another study, although results from which showed that exosomes elicited a negative response when the dosage was above 2 × 10^6^ particles. They therefore concluded that the beneficial effects of adipose mesenchymal stromal cells-derived extracellular vesicles can only be harnessed by identifying the effective dose.^50^ Unfortunately, the dose and unit of exosomes have not yet been standardized. It will be interesting to explore the appropriate dosage and pharmacokinetics of MSC-Exo in the future according to this phenomenon.

AM has a vital impact on natural immunity, which can release inflammatory cytokines and interact with other immune cells to facilitate the development of ALI. Researchers found that AM pyroptosis caused a complex of inflammatory responses and signaling transduction.^51^ AM pyroptosis exaggerated lung inflammation and promoted the pathogenesis of ALI. It has been reported that caspase-1-dependent pyroptosis in macrophages is associated with the secretion of inflammatory factors IL-1β and IL-18.^52^ Besides, caspase-1-deficient mice can resist endotoxic shock caused by large doses of LPS.^53^ We also observed that AM pyroptosis participated in the LPS-induced ALI.

MSCs-Exo reduce inflammation and alleviate lung injury, through their immunomodulatory properties or inhibiting endothelial cell apoptosis.^54,55^ However, the exact mechanism underlying the anti-inflammatory effects of MSCs-Exo is not well understood. Recent studies showed the therapeutic effects of MSCs for many diseases partly depended on their function in regulating cell death.^56^ MSCs have the function of preventing macrophage pyroptosis.^30,57^ Exosomes are one of the essential paracrine mediators of MSCs, which may have similar functions to parental cells. It has been demonstrated that exosomes derived from bone marrow mesenchymal stem cells can inhibit pyroptosis in epithelial cells, thus alleviating lung ischemic-reperfusion injury.^58^ In another study, adipose-derived stem cell-derived exosomes were found to mitigate pyroptosis-related gene expression in AMs induced by smoking.^59^ However, the specific role of exosomes in the occurrence of pyroptosis was not fully elucidated. In our study, we discovered that MSCs-Exo may reduces inflammation by inhibiting cell pyroptosis. We further analyzed the effects of MSCs-Exo on AM pyroptosis in vivo and in vitro. First, we compared the effects of Ac-YVAD-CMK and MSCs-Exo on pyroptosis of primarily cultured AM. Ac-YVAD-CMK is a specific and irreversible inhibitor of pyroptosis. Ac-YVAD-CMK has been shown to inhibit the secretion of mature IL-1β by blocking caspase-1 activation, which alleviated *A. baumannii*-induced lung injury.^60^ Ac-YVAD-CMK pretreatment also alleviated LPS-induced lung injury by inhibiting AM pyroptosis.^61^ Our results showed that MSCs-Exo directly inhibited AM pyroptosis, with an effect comparable to Ac-YVAD-CMK. We further explored how MSCs-Exo inhibited AM pyroptosis, and found that MSCs-Exo inhibited caspase-1 expression and activation. These results showed that MSCs-Exo may inhibit AM pyroptosis by suppressing caspase-1 activation.

To further identify the components and the specific regulatory mechanisms involved in inhibiting AM pyroptosis by MSCs-Exo, we analyzed the components of MSCs-Exo and MRC-5-Exo by miRNA sequencing and proteomic analysis. Our results showed that 30 upregulated miRNAs could target NLRP3, CASP1, GSDMD, IL1B, and IL18 genes. Researchers found that miR-22-3p plays a protective role in asthma. Overexpression of miR-22-3p attenuated asthma in mice by regulating NLRP3-caspase-1-IL-1β axis.^62^ Besides, miR-22-3p could directly target NLRP3 according to dual-luciferase reporter assay.^63^ Studies found that miR-214-3p contains caspase-1-binding sites, overexpression of miR-214-3p decreased caspase-1 levels in fibroblasts.^64^ Another study also verified that transfection of miR-214-3p reduced CASP1 activity by luciferase assay and decreased gene expression of CASP1 by qPCR experiment.^65^ MiR-221-5p regulated inflammatory responses in acute gouty arthritis, which can target IL1B gene. Its overexpression reduced the expression of inflammatory factors such as TNF-α, IL-8, and IL-1β.^66^ The relationship between some upregulated miRNAs and GSDMD gene has been verified. MiR-18a-3p not only directly bound to GSDMD but also reduced the levels of LDH, IL-1β, and IL-18 induced by LPS.^67^ Our results showed that miR-16-5p may target GSDMD. Another studied found that Casp1 was the direct target gene of miR-16-5p.^68^ These results suggested that multiple miRNAs enriched in MSCs-Exo may have the potential to target the caspase-1-related pyroptosis genes, which needs to be verified by more experiments.

Our proteomic analysis of differential proteins between MSCs-Exo and MRC-5-Exo indicated that MSCs-Exo may inhibit AM pyroptosis through immunoregulation. According to Reactome Gene Sets and KEGG analysis, upregulated proteins in MSCs-Exo are mostly enriched in pathways of binding and uptake of ligands by scavenger receptors, vesicle-mediated transport, and complement and coagulation cascades. This added evidence to explain that MSCs-Exo participated in immune system response and response to stimulus. Some studies had represented similar conclusions that MSCs-EVs exert immunomodulatory function by delivering proteins.^69,70^ Interestingly, according to Reactome Gene Sets and KEGG analysis, enriched proteins in MRC-5-Exo mostly enriched in the pathway of axon guidance, peptide chain elongation, eukaryotic translation elongation, and ribosome. It might provide an alternate explanation for MSCs-Exo inhibiting AM pyroptosis by reducing the biosynthesis of pyroptosis-related proteins.^71^ Further investigations are of substantial importance to elucidate these potential mechanisms.

## Conclusions

Findings from our study suggested that MSCs-Exo have anti-inflammatory properties like their parental cells and therapeutic effects against ALI were dose-dependent. In addition, we provided evidence that pyroptosis of AM plays an important role in LPS-induced ALI. We also demonstrated that MSCs-Exo reduce inflammation by inhibiting AM pyroptosis. MiRNA sequencing and proteomic results showed that MSCs-Exo may inhibit pyroptosis by miRNAs targeting caspase-1-mediated pathway, and by proteins possessing immunoregulation functions. In summary, we demonstrated that MSCs-Exo has the potential to be applied in the development of new therapeutic strategies for the early stage of ALI.

## Supplementary material

Supplementary material is available at *Stem Cells Translational Medicine* online.

szad094_suppl_Supplementary_Materials

## Data Availability

The data underlying this article are available in the article and in its online supplementary material.

## References

[1] Matthay MA , Zemans RL. The acute respiratory distress syndrome: pathogenesis and treatment. Annu Rev Pathol. 2011;6: 1, 147-163. 10.1146/annurev-pathol-011110-130158 20936936 PMC3108259

[2] Meyer NJ , Gattinoni L, Calfee CS. Acute respiratory distress syndrome. Lancet. 2021;398(10300):622-637. 10.1016/S0140-6736(21)00439-6 34217425 PMC8248927

[3] Fan E , Brodie D, Slutsky AS. Acute respiratory distress syndrome: advances in diagnosis and treatment. JAMA. 2018;319(7):698-710. 10.1001/jama.2017.21907 29466596

[4] Matthay MA , Zemans RL, Zimmerman GA, et al. Acute respiratory distress syndrome. Nat Rev Dis Primers. 2019;5(1):18. 10.1038/s41572-019-0069-0 30872586 PMC6709677

[5] Bos LDJ , Ware LB. Acute respiratory distress syndrome: causes, pathophysiology, and phenotypes. Lancet. 2022;400(10358):1145-1156. 10.1016/S0140-6736(22)01485-4 36070787

[6] Bellani G , Laffey JG, Pham T, et al; LUNG SAFE Investigators. Epidemiology, patterns of care, and mortality for patients with acute respiratory distress syndrome in intensive care units in 50 countries. JAMA. 2016;315(8):788-800. 10.1001/jama.2016.0291 26903337

[7] Fan EKY , Fan J. Regulation of alveolar macrophage death in acute lung inflammation. Respir Res. 2018;19(1):50. 10.1186/s12931-018-0756-5 29587748 PMC5872399

[8] Fink SL , Cookson BT. Caspase-1-dependent pore formation during pyroptosis leads to osmotic lysis of infected host macrophages. Cell Microbiol. 2006;8(11):1812-1825. 10.1111/j.1462-5822.2006.00751.x 16824040

[9] Shi J , Zhao Y, Wang K, et al. Cleavage of GSDMD by inflammatory caspases determines pyroptotic cell death. Nature. 2015;526(7575):660-665. 10.1038/nature15514 26375003

[10] Pinkerton JW , Kim RY, Robertson AAB, et al. Inflammasomes in the lung. Mol Immunol. 2017 Jun;86:44-55. 10.1016/j.molimm.2017.01.014 28129896

[11] He X , Qian Y, Li Z, et al. TLR4-upregulated IL-1beta and IL-1RI promote alveolar macrophage pyroptosis and lung inflammation through an autocrine mechanism. Sci Rep. 2016 Aug;6:31663. 10.1038/srep31663 27526865 PMC4985817

[12] Mohammadian M , Shamsasenjan K, Lotfi Nezhad P, et al. Mesenchymal stem cells: new aspect in cell-based regenerative therapy. Adv Pharm Bull. 2013;3(2):433-437. 10.5681/apb.2013.070 24312873 PMC3848236

[13] Maron-Gutierrez T , Silva JD, Asensi KD, et al. Effects of mesenchymal stem cell therapy on the time course of pulmonary remodeling depend on the etiology of lung injury in mice. Crit Care Med. 2013;41(11):e319-e333. 10.1097/CCM.0b013e31828a663e 23760104

[14] Liang X , Ding Y, Zhang Y, Tse H-F, Lian Q. Paracrine mechanisms of mesenchymal stem cell-based therapy: current status and perspectives. Cell Transplant. 2014;23(9):1045-1059. 10.3727/096368913X667709 23676629

[15] Kalluri R , LeBleu VS. The biology, function, and biomedical applications of exosomes. Science. 2020 Feb 7;367(6478):eaau6977. 10.1126/science.aau6977 32029601 PMC7717626

[16] Zhou Y , Xu H, Xu W, et al. Exosomes released by human umbilical cord mesenchymal stem cells protect against cisplatin-induced renal oxidative stress and apoptosis in vivo and in vitro. Stem Cell Res Ther. 2013;4(2):34. 10.1186/scrt194 23618405 PMC3707035

[17] Nakamura Y , Miyaki S, Ishitobi H, et al. Mesenchymal-stem-cell-derived exosomes accelerate skeletal muscle regeneration. FEBS Lett. 2015;589(11):1257-1265. 10.1016/j.febslet.2015.03.031 25862500

[18] Shao L , Zhang Y, Lan B, et al. MiRNA-sequence indicates that mesenchymal stem cells and exosomes have similar mechanism to enhance cardiac repair. Biomed Res Int. 2017;2017:4150705. 10.1155/2017/4150705 28203568 PMC5292186

[19] Zhang Y , Chopp M, Meng Y, et al. Effect of exosomes derived from multipluripotent mesenchymal stromal cells on functional recovery and neurovascular plasticity in rats after traumatic brain injury. J Neurosurg. 2015;122(4):856-867. 10.3171/2014.11.JNS14770 25594326 PMC4382456

[20] Heydari R , Koohi F, Rasouli M, et al. Exosomes as rheumatoid arthritis diagnostic biomarkers and therapeutic agents. Vaccines (Basel) 2023;11(3):687. 10.3390/vaccines11030687 36992270 PMC10057381

[21] Ghasempour E , Hesami S, Movahed E, Keshel SH, Doroudian M. Mesenchymal stem cell-derived exosomes as a new therapeutic strategy in the brain tumors. Stem Cell Res Ther. 2022;13(1):527. 10.1186/s13287-022-03212-4 36536420 PMC9764546

[22] Azhdari MH , Goodarzi N, Doroudian M, MacLoughlin R. Molecular insight into the therapeutic effects of stem cell-derived exosomes in respiratory diseases and the potential for pulmonary delivery. Int J Mol Sci . 2022;23(11):6273. 10.3390/ijms23116273 35682948 PMC9181737

[23] Doroudian M , Armstrong ME, Donnelly SC. Nano-based therapies for acute and chronic lung diseases. In: Ribeiro de Araujo D, Carneiro-Ramos M, eds. Biotechnology Applied to Inflammatory Diseases: Cellular Mechanisms and Nanomedicine. Springer Nature Singapore; 2023:271–86.

[24] Coppin L , Sokal E, Stephenne X. Thrombogenic risk induced by intravascular mesenchymal stem cell therapy: current status and future perspectives. Cells. 2019;8(10):1160. 10.3390/cells8101160 31569696 PMC6829440

[25] Forsberg MH , Kink JA, Hematti P, Capitini CM. Mesenchymal stromal cells and exosomes: progress and challenges. Front Cell Dev Biol. 2020 Jul 17;8:665. 10.3389/fcell.2020.00665 32766255 PMC7379234

[26] Willis GR , Fernandez-Gonzalez A, Reis M, Mitsialis SA, Kourembanas S. Macrophage immunomodulation: the gatekeeper for mesenchymal stem cell derived-exosomes in pulmonary arterial hypertension? Int J Mol Sci. 2018;19(9):2534. 10.3390/ijms19092534 30150544 PMC6164282

[27] Dong B , Wang C, Zhang J, et al. Exosomes from human umbilical cord mesenchymal stem cells attenuate the inflammation of severe steroid-resistant asthma by reshaping macrophage polarization. Stem Cell Res Ther. 2021;12(1):204. 10.1186/s13287-021-02244-6 33761997 PMC7988945

[28] Self-Fordham JB , Naqvi AR, Uttamani JR, Kulkarni V, Nares S. MicroRNA: dynamic regulators of macrophage polarization and plasticity. Front Immunol. 2017 Aug 31;8:1062. 10.3389/fimmu.2017.01062 28912781 PMC5583156

[29] Ma J , Chen L, Zhu X, et al. Mesenchymal stem cell-derived exosomal miR-21a-5p promotes M2 macrophage polarization and reduces macrophage infiltration to attenuate atherosclerosis. Acta Biochim Biophys Sin (Shanghai). 2021;53(9):1227-1236. 10.1093/abbs/gmab102 34350954

[30] Naji A , Suganuma N, Espagnolle N, et al. Rationale for determining the functional potency of mesenchymal stem cells in preventing regulated cell death for therapeutic use. Stem Cells Transl Med. 2017;6(3):713-719. 10.5966/sctm.2016-0289 28297565 PMC5442793

[31] Corotchi MC , Popa MA, Remes A, et al. Isolation method and xeno-free culture conditions influence multipotent differentiation capacity of human Wharton’s jelly-derived mesenchymal stem cells. Stem Cell Res Ther. 2013;4(4):81. 10.1186/scrt232 23845279 PMC3854854

[32] Jacobs JP , Jones CM, Baille JP. Characteristics of a human di ploid cell designated MRC-5. Nature. 1970;227(5254):168-170. 10.1038/227168a0 4316953

[33] Jacobs JP. The status of human diploid cell strain MRC-5 as an approved substrate for the production of viral vaccines. J Biol Stand. 1976;4(2):97-99. 10.1016/0092-1157(76)90018-4 932048

[34] Busch CJ , Favret J, Geirsdottir L, Molawi K, Sieweke MH. Isolation and long-term cultivation of mouse alveolar macrophages. Bio Protoc. 2019;9(14):e3302. 10.21769/BioProtoc.3302 PMC6944498 31909091

[35] Matute-Bello G , Downey G, Moore BB, et al; Acute Lung Injury in Animals Study Group. An Official American Thoracic Society Workshop Report: features and measurements of experimental acute lung injury in animals. Am J Respir Cell Mol Biol. 2011;44(5):725-738. 10.1165/rcmb.2009-0210ST 21531958 PMC7328339

[36] Kramer A , Green J, Pollard J Jr, Tugendreich S. Causal analysis approaches in ingenuity pathway analysis. Bioinformatics. 2014;30(4):523-530. 10.1093/bioinformatics/btt703 24336805 PMC3928520

[37] Zhou Y , Zhou B, Pache L, et al. Metascape provides a biologist-oriented resource for the analysis of systems-level datasets. Nat Commun. 2019;10(1):1523. 10.1038/s41467-019-09234-6 30944313 PMC6447622

[38] Perez-Riverol Y , Bai J, Bandla C, et al. The PRIDE database resources in 2022: a hub for mass spectrometry-based proteomics evidences. Nucleic Acids Res. 2022;50(D1):D543-D552. 10.1093/nar/gkab1038 34723319 PMC8728295

[39] Wu X , Smith TG, Rupprecht CE. From brain passage to cell adaptation: the road of human rabies vaccine development. Expert Rev Vaccines. 2011;10(11):1597-1608. 10.1586/erv.11.140 22043958

[40] Garcia-Calvo M , Peterson EP, Leiting B, et al. Inhibition of human caspases by peptide-based and macromolecular inhibitors. J Biol Chem. 1998;273(49):32608-32613. 10.1074/jbc.273.49.32608 9829999

[41] Agarwal V , Bell GW, Nam JW, Bartel DP. Predicting effective microRNA target sites in mammalian mRNAs. Elife. 2015;4(Aug 12):e05005. 26267216 10.7554/eLife.05005 PMC4532895

[42] Leng Z , Zhu R, Hou W, et al. Transplantation of ACE2(-) mesenchymal stem cells improves the outcome of patients with COVID-19 pneumonia. Aging Dis. 2020;11(2):216-228. 10.14336/AD.2020.0228 32257537 PMC7069465

[43] Thery C , Witwer KW, Aikawa E, et al. Minimal information for studies of extracellular vesicles 2018 (MISEV2018): a position statement of the International Society for Extracellular Vesicles and update of the MISEV2014 guidelines. J Extracell Vesicles. 2018;7(1):1535750. 10.1080/20013078.2018.1535750 30637094 PMC6322352

[44] Cai G , Cai G, Zhou H, et al. Mesenchymal stem cell-derived exosome miR-542-3p suppresses inflammation and prevents cerebral infarction. Stem Cell Res Ther. 2021;12(1):2. 10.1186/s13287-020-02030-w 33407827 PMC7786953

[45] Willis GR , Fernandez-Gonzalez A, Anastas J, et al. Mesenchymal stromal cell exosomes ameliorate experimental bronchopulmonary dysplasia and restore lung function through macrophage immunomodulation. Am J Respir Crit Care Med. 2018;197(1):104-116. 10.1164/rccm.201705-0925OC 28853608 PMC5765387

[46] Jiang L , Zhang S, Hu H, et al. Exosomes derived from human umbilical cord mesenchymal stem cells alleviate acute liver failure by reducing the activity of the NLRP3 inflammasome in macrophages. Biochem Biophys Res Commun. 2019;508(3):735-741. 10.1016/j.bbrc.2018.11.189 30528233

[47] Mao Q , Liang XL, Zhang CL, Pang Y-H, Lu Y-X. LncRNA KLF3-AS1 in human mesenchymal stem cell-derived exosomes ameliorates pyroptosis of cardiomyocytes and myocardial infarction through miR-138-5p/Sirt1 axis. Stem Cell Res Ther. 2019;10(1):393. 10.1186/s13287-019-1522-4 31847890 PMC6918658

[48] Li QC , Liang Y, Su ZB. Prophylactic treatment with MSC-derived exosomes attenuates traumatic acute lung injury in rats. Am J Physiol Lung Cell Mol Physiol. 2019;316(6):L1107-L1117. 10.1152/ajplung.00391.2018 30892077

[49] Yang S , Liu P, Gao T, et al. Every road leads to Rome: therapeutic effect and mechanism of the extracellular vesicles of human embryonic stem cell-derived immune and matrix regulatory cells administered to mouse models of pulmonary fibrosis through different routes. Stem Cell Res Ther. 2022;13(1):163. 35413874 10.1186/s13287-022-02839-7 PMC9006546

[50] Shi MM , Yang QY, Monsel A, et al. Preclinical efficacy and clinical safety of clinical-grade nebulized allogenic adipose mesenchymal stromal cells-derived extracellular vesicles. J Extracell Vesicles. 2021;10(10):e12134. 10.1002/jev2.12134 34429860 PMC8363910

[51] Liu B , He R, Zhang L, et al. Inflammatory caspases drive pyroptosis in acute lung injury. Front Pharmacol. 2021 Feb 5;12:631256. 10.3389/fphar.2021.631256 33613295 PMC7892432

[52] Jorgensen I , Miao EA. Pyroptotic cell death defends against intracellular pathogens. Immunol Rev. 2015;265(1):130-142. 10.1111/imr.12287 25879289 PMC4400865

[53] Li P , Allen H, Banerjee S, et al. Mice deficient in IL-1 beta-converting enzyme are defective in production of mature IL-1 beta and resistant to endotoxic shock. Cell. 1995;80(3):401-411. 10.1016/0092-8674(95)90490-5 7859282

[54] Khatri M , Richardson LA, Meulia T. Mesenchymal stem cell-derived extracellular vesicles attenuate influenza virus-induced acute lung injury in a pig model. Stem Cell Res Ther. 2018;9(1):17. 10.1186/s13287-018-0774-8 29378639 PMC5789598

[55] Shah TG , Predescu D, Predescu S. Mesenchymal stem cells-derived extracellular vesicles in acute respiratory distress syndrome: a review of current literature and potential future treatment options. Clin Transl Med. 2019;8(1):25. 10.1186/s40169-019-0242-9 31512000 PMC6739436

[56] Naji A , Favier B, Deschaseaux F, et al. Mesenchymal stem/stromal cell function in modulating cell death. Stem Cell Res Ther. 2019;10(1):56. 10.1186/s13287-019-1158-4 30760307 PMC6374902

[57] Naji A , Muzembo BA, Yagyu K, et al. Endocytosis of indium-tin-oxide nanoparticles by macrophages provokes pyroptosis requiring NLRP3-ASC-Caspase1 axis that can be prevented by mesenchymal stem cells. Sci Rep. 2016 May 19;6:26162. 10.1038/srep26162 27194621 PMC4872131

[58] Sun ZL , You T, Zhang BH, Liu Y, Liu J. Bone marrow mesenchymal stem cell-derived exosomes miR-202-5p inhibited pyroptosis to alleviate lung ischemic-reperfusion injury by targeting CMPK2. Kaohsiung J Med Sci. 2023;39(7):688-698. 10.1002/kjm2.12688 37092308 PMC11895865

[59] Zhu Z , Lian X, Su X, et al. Exosomes derived from adipose-derived stem cells alleviate cigarette smoke-induced lung inflammation and injury by inhibiting alveolar macrophages pyroptosis. Respir Res. 2022;23(1):5. 10.1186/s12931-022-01926-w 35016678 PMC8753876

[60] Zhang Y , Zhou X, Zhang H, Huan C, Ye Z. Caspase-1 inhibitor AC-YVAD-CMK blocks IL-1beta secretion of bone marrow-derived macrophages induced by *Acinetobacter baumannii*. Xi Bao Yu Fen Zi Mian Yi Xue Za Zhi 2017;33(12):1594-1599. 29382416

[61] Wu DD , Pan PH, Liu B, et al. Inhibition of alveolar macrophage pyroptosis reduces lipopolysaccharide-induced acute lung injury in mice. Chin Med J (Engl). 2015;128(19):2638-2645. 10.4103/0366-6999.166039 26415803 PMC4736856

[62] Guo S , Chen R, Zhang L, et al. microRNA-22-3p plays a protective role in a murine asthma model through the inhibition of the NLRP3-caspase-1-IL-1beta axis. Exp Physiol. 2021;106(8):1829-1838. 10.1113/EP089575 33932961

[63] Wang X , Chi J, Dong B, et al. MiR-223-3p and miR-22-3p inhibit monosodium urate-induced gouty inflammation by targeting NLRP3. Int J Rheum Dis. 2021;24(4):599-607. 10.1111/1756-185X.14089 33650318

[64] Yang F , Qin Y, Lv J, et al. Silencing long non-coding RNA Kcnq1ot1 alleviates pyroptosis and fibrosis in diabetic cardiomyopathy. Cell Death Dis. 2018;9(10):1000. 10.1038/s41419-018-1029-4 30250027 PMC6155223

[65] Xiao W , Zheng D, Chen X, et al. Long non-coding RNA MIAT is involved in the regulation of pyroptosis in diabetic cardiomyopathy via targeting miR-214-3p. iScience. 2021;24(12):103518. 10.1016/j.isci.2021.103518 34950859 PMC8671938

[66] Li G , Zhang H, Ma H, et al. MiR-221-5p is involved in the regulation of inflammatory responses in acute gouty arthritis by targeting IL-1beta. Int J Rheum Dis. 2021;24(3):335-340. 33201565 10.1111/1756-185X.14028

[67] Deng J , Tan W, Luo Q, et al. Long non-coding RNA MEG3 promotes renal tubular epithelial cell pyroptosis by regulating the miR-18a-3p/GSDMD pathway in lipopolysaccharide-induced acute kidney injury. Front Physiol. 2021 Apr 29;12:663216. 10.3389/fphys.2021.663216 34012408 PMC8128073

[68] Wang X , Li Y, Li J, Li S, Wang F. Mechanism of METTL3-mediated m(6)A modification in cardiomyocyte pyroptosis and myocardial ischemia-reperfusion injury. Cardiovasc Drugs Ther. 2022 Jun;37(3):435-448. 35066738 10.1007/s10557-021-07300-0

[69] Mardpour S , Hamidieh AA, Taleahmad S, et al. Interaction between mesenchymal stromal cell-derived extracellular vesicles and immune cells by distinct protein content. J Cell Physiol. 2019;234(6):8249-8258. 10.1002/jcp.27669 30378105

[70] Wang ZG , He ZY, Liang S, et al. Comprehensive proteomic analysis of exosomes derived from human bone marrow, adipose tissue, and umbilical cord mesenchymal stem cells. Stem Cell Res Ther. 2020;11(1):511. 10.1186/s13287-020-02032-8 33246507 PMC7694919

[71] Fu Y , Shen J, Li Y, et al. Inhibition of the PERK/TXNIP/NLRP3 axis by baicalin reduces NLRP3 inflammasome-mediated pyroptosis in macrophages infected with mycobacterium tuberculosis. Mediators Inflamm. 2021 Nov 8;2021:1805147. 10.1155/2021/1805147 34790063 PMC8592748

