# Supplementary material for: Mesenchymal Stem Cells-Derived Exosomes Alleviate Acute Lung Injury by Inhibiting Alveolar Macrophage Pyroptosis
Source: Stem Cells Transl Med. 2024 Feb 13;13(4):371–86. doi: 10.1093/stcltm/szad094 (PMC11016849; doi:10.1093/stcltm/szad094)
Supplement: szad094_suppl_Supplementary_Materials [file szad094_suppl_supplementary_materials.zip › szad094_suppl_Supplementary_Tables_S1.docx]

**Table S1. The differential miRNAs between MSCs-Exo and MRC-5-Exo.**

| **MiRNA** | **Log_2_FC** | ***P*-value** | **FDR** |
| --- | --- | --- | --- |
| hsa-let-7a-5p  hsa-let-7b-5p  hsa-let-7c-5p  hsa-let-7d-3p  hsa-let-7d-5p  hsa-let-7e-5p  hsa-let-7f-1-3p  hsa-let-7f-5p  hsa-let-7g-5p  hsa-let-7i-5p  hsa-miR-100-5p  hsa-miR-101-3p  hsa-miR-10396a-3p  hsa-miR-10396b-3p  hsa-miR-10398-5p  hsa-miR-10399-5p  hsa-miR-103a-3p  hsa-miR-10400-5p  hsa-miR-10401-3p  hsa-miR-10401-5p  hsa-miR-10523-5p  hsa-miR-106a-5p  hsa-miR-106b-3p  hsa-miR-107  hsa-miR-10a-3p  hsa-miR-10b-5p  hsa-miR-11181-3p  hsa-miR-11399  hsa-miR-11401  hsa-miR-1178-3p  hsa-miR-1193  hsa-miR-1197  hsa-miR-1199-5p  hsa-miR-1200  hsa-miR-1208  hsa-miR-12114  hsa-miR-12116  hsa-miR-12119  hsa-miR-12122  hsa-miR-12125  hsa-miR-12128  hsa-miR-12132  hsa-miR-12136  hsa-miR-1226-3p  hsa-miR-1237-3p  hsa-miR-1238-3p  hsa-miR-1238-5p  hsa-miR-1244  hsa-miR-1258  hsa-miR-125b-1-3p  hsa-miR-125b-5p  hsa-miR-127-3p  hsa-miR-127-5p  hsa-miR-1271-5p  hsa-miR-1273c  hsa-miR-1273h-3p  hsa-miR-1275  hsa-miR-1277-5p  hsa-miR-1278  hsa-miR-128-2-5p  hsa-miR-128-3p  hsa-miR-129-5p  hsa-miR-1290  hsa-miR-1292-3p  hsa-miR-1292-5p  hsa-miR-1294  hsa-miR-1295b-5p  hsa-miR-1296-5p  hsa-miR-1298-3p  hsa-miR-1299  hsa-miR-1303  hsa-miR-1304-3p  hsa-miR-1306-5p  hsa-miR-130a-3p  hsa-miR-130b-3p  hsa-miR-130b-5p  hsa-miR-132-3p  hsa-miR-133a-5p  hsa-miR-134-5p  hsa-miR-1343-3p  hsa-miR-1343-5p  hsa-miR-135b-3p  hsa-miR-136-3p  hsa-miR-137-3p  hsa-miR-138-1-3p  hsa-miR-139-3p  hsa-miR-140-3p  hsa-miR-140-5p  hsa-miR-141-3p  hsa-miR-141-5p  hsa-miR-143-3p  hsa-miR-143-5p  hsa-miR-145-3p  hsa-miR-145-5p  hsa-miR-146a-5p  hsa-miR-146b-5p  hsa-miR-147b-3p  hsa-miR-148a-3p  hsa-miR-148b-3p  hsa-miR-150-5p  hsa-miR-151a-3p  hsa-miR-151a-5p  hsa-miR-152-3p  hsa-miR-154-5p  hsa-miR-155-5p  hsa-miR-1587  hsa-miR-15a-5p  hsa-miR-15b-3p  hsa-miR-15b-5p  hsa-miR-16-5p  hsa-miR-17-5p  hsa-miR-181a-2-3p  hsa-miR-181a-3p  hsa-miR-181a-5p  hsa-miR-181b-3p  hsa-miR-181b-5p  hsa-miR-181d-5p  hsa-miR-183-5p  hsa-miR-184  hsa-miR-185-5p  hsa-miR-186-5p  hsa-miR-18a-3p  hsa-miR-1908-5p  hsa-miR-1909-5p  hsa-miR-190a-5p  hsa-miR-190b-5p  hsa-miR-191-5p  hsa-miR-1910-5p  hsa-miR-1913  hsa-miR-192-3p  hsa-miR-192-5p  hsa-miR-193a-5p  hsa-miR-193b-3p  hsa-miR-196a-5p  hsa-miR-196b-5p  hsa-miR-1976  hsa-miR-199a-3p  hsa-miR-199a-5p  hsa-miR-199b-3p  hsa-miR-199b-5p  hsa-miR-19a-3p  hsa-miR-19b-1-5p  hsa-miR-19b-2-5p  hsa-miR-19b-3p  hsa-miR-200b-5p  hsa-miR-202-3p  hsa-miR-203b-3p  hsa-miR-203b-5p  hsa-miR-204-3p  hsa-miR-205-5p  hsa-miR-20a-5p  hsa-miR-20b-3p  hsa-miR-21-3p  hsa-miR-21-5p  hsa-miR-2115-5p  hsa-miR-214-3p  hsa-miR-215-5p  hsa-miR-218-1-3p  hsa-miR-218-5p  hsa-miR-22-3p  hsa-miR-221-3p  hsa-miR-221-5p  hsa-miR-222-3p  hsa-miR-224-5p  hsa-miR-2278  hsa-miR-2392  hsa-miR-23a-3p  hsa-miR-23b-3p  hsa-miR-24-3p  hsa-miR-2467-3p  hsa-miR-25-3p  hsa-miR-26a-5p  hsa-miR-26b-5p  hsa-miR-27a-3p  hsa-miR-27a-5p  hsa-miR-27b-3p  hsa-miR-28-3p  hsa-miR-2861  hsa-miR-297  hsa-miR-298  hsa-miR-299-3p  hsa-miR-29a-3p  hsa-miR-29b-3p  hsa-miR-29c-3p  hsa-miR-29c-5p  hsa-miR-301a-3p  hsa-miR-301b-3p  hsa-miR-302a-3p  hsa-miR-302b-5p  hsa-miR-302c-3p  hsa-miR-30a-3p  hsa-miR-30a-5p  hsa-miR-30b-3p  hsa-miR-30c-2-3p  hsa-miR-30c-5p  hsa-miR-30d-5p  hsa-miR-30e-3p  hsa-miR-30e-5p  hsa-miR-31-3p  hsa-miR-31-5p  hsa-miR-3117-3p  hsa-miR-3118  hsa-miR-3119  hsa-miR-3125  hsa-miR-3126-5p  hsa-miR-3134  hsa-miR-3135a  hsa-miR-3144-3p  hsa-miR-3151-3p  hsa-miR-3152-5p  hsa-miR-3157-5p  hsa-miR-3162-3p  hsa-miR-3162-5p  hsa-miR-3165  hsa-miR-3179  hsa-miR-3181  hsa-miR-3182  hsa-miR-3184-5p  hsa-miR-3189-5p  hsa-miR-3190-5p  hsa-miR-3193  hsa-miR-3195  hsa-miR-320a-3p  hsa-miR-320b  hsa-miR-320c  hsa-miR-323a-3p  hsa-miR-323b-3p  hsa-miR-323b-5p  hsa-miR-324-5p  hsa-miR-329-3p  hsa-miR-335-5p  hsa-miR-337-3p  hsa-miR-337-5p  hsa-miR-339-5p  hsa-miR-345-5p  hsa-miR-34a-5p  hsa-miR-34b-5p  hsa-miR-34c-5p  hsa-miR-3529-3p  hsa-miR-3609  hsa-miR-361-3p  hsa-miR-361-5p  hsa-miR-3612  hsa-miR-3615  hsa-miR-3617-5p  hsa-miR-3621  hsa-miR-3622b-3p  hsa-miR-363-5p  hsa-miR-3648  hsa-miR-3654  hsa-miR-365a-3p  hsa-miR-365b-3p  hsa-miR-365b-5p  hsa-miR-3660  hsa-miR-3666  hsa-miR-3667-5p  hsa-miR-367-3p  hsa-miR-3671  hsa-miR-3672  hsa-miR-3679-3p  hsa-miR-3680-5p  hsa-miR-3683  hsa-miR-3684  hsa-miR-369-3p  hsa-miR-369-5p  hsa-miR-373-3p  hsa-miR-374a-3p  hsa-miR-374a-5p  hsa-miR-374b-3p  hsa-miR-374b-5p  hsa-miR-375-5p  hsa-miR-376a-2-5p  hsa-miR-376a-3p  hsa-miR-376b-3p  hsa-miR-376b-5p  hsa-miR-376c-3p  hsa-miR-376c-5p  hsa-miR-377-3p  hsa-miR-378a-3p  hsa-miR-378b  hsa-miR-378j  hsa-miR-379-5p  hsa-miR-380-5p  hsa-miR-381-3p  hsa-miR-382-3p  hsa-miR-382-5p  hsa-miR-3917  hsa-miR-3919  hsa-miR-3920  hsa-miR-3922-3p  hsa-miR-3934-3p  hsa-miR-3940-5p  hsa-miR-3942-3p  hsa-miR-3944-5p  hsa-miR-3972  hsa-miR-3974  hsa-miR-3978  hsa-miR-409-3p  hsa-miR-410-3p  hsa-miR-411-3p  hsa-miR-411-5p  hsa-miR-412-5p  hsa-miR-421  hsa-miR-423-3p  hsa-miR-423-5p  hsa-miR-424-3p  hsa-miR-424-5p  hsa-miR-425-3p  hsa-miR-425-5p  hsa-miR-4263  hsa-miR-4269  hsa-miR-4272  hsa-miR-4279  hsa-miR-4281  hsa-miR-4282  hsa-miR-4288  hsa-miR-429  hsa-miR-4293  hsa-miR-4298  hsa-miR-4303  hsa-miR-4305  hsa-miR-4307  hsa-miR-431-5p  hsa-miR-4310  hsa-miR-4319  hsa-miR-432-3p  hsa-miR-432-5p  hsa-miR-433-5p  hsa-miR-4430  hsa-miR-4435  hsa-miR-4438  hsa-miR-4445-5p  hsa-miR-4446-3p  hsa-miR-4448  hsa-miR-4449  hsa-miR-4450  hsa-miR-4457  hsa-miR-4458  hsa-miR-4462  hsa-miR-4463  hsa-miR-4474-5p  hsa-miR-4479  hsa-miR-4484  hsa-miR-4487  hsa-miR-4493  hsa-miR-4496  hsa-miR-449c-3p  hsa-miR-449c-5p  hsa-miR-450b-5p  hsa-miR-4513  hsa-miR-4515  hsa-miR-452-5p  hsa-miR-4530  hsa-miR-4531  hsa-miR-4534  hsa-miR-454-3p  hsa-miR-4540  hsa-miR-455-3p  hsa-miR-455-5p  hsa-miR-4632-3p  hsa-miR-4634  hsa-miR-4635  hsa-miR-4636  hsa-miR-4640-3p  hsa-miR-4644  hsa-miR-4645-3p  hsa-miR-4646-3p  hsa-miR-4649-3p  hsa-miR-4649-5p  hsa-miR-4651  hsa-miR-4652-3p  hsa-miR-4652-5p  hsa-miR-4660  hsa-miR-4666a-5p  hsa-miR-4666b  hsa-miR-4668-3p  hsa-miR-4670-5p  hsa-miR-4678  hsa-miR-4680-5p  hsa-miR-4681  hsa-miR-4688  hsa-miR-4693-3p  hsa-miR-4693-5p  hsa-miR-4694-5p  hsa-miR-4695-3p  hsa-miR-4700-3p  hsa-miR-4703-3p  hsa-miR-4708-5p  hsa-miR-4715-5p  hsa-miR-4721  hsa-miR-4725-3p  hsa-miR-4727-5p  hsa-miR-4732-3p  hsa-miR-4734  hsa-miR-4737  hsa-miR-4738-3p  hsa-miR-4741  hsa-miR-4744  hsa-miR-4746-3p  hsa-miR-4751  hsa-miR-4753-3p  hsa-miR-4753-5p  hsa-miR-4754  hsa-miR-4755-3p  hsa-miR-4758-3p  hsa-miR-4758-5p  hsa-miR-4759  hsa-miR-4762-3p  hsa-miR-4763-3p  hsa-miR-4764-3p  hsa-miR-4772-5p  hsa-miR-4774-3p  hsa-miR-4775  hsa-miR-4779  hsa-miR-4785  hsa-miR-4788  hsa-miR-4800-5p  hsa-miR-4803  hsa-miR-485-5p  hsa-miR-486-3p  hsa-miR-486-5p  hsa-miR-487a-3p  hsa-miR-487a-5p  hsa-miR-487b-3p  hsa-miR-493-3p  hsa-miR-493-5p  hsa-miR-494-3p  hsa-miR-496  hsa-miR-4999-3p  hsa-miR-5001-5p  hsa-miR-5002-3p  hsa-miR-5009-5p  hsa-miR-500a-3p  hsa-miR-501-3p  hsa-miR-5011-5p  hsa-miR-502-3p  hsa-miR-503-3p  hsa-miR-503-5p  hsa-miR-504-5p  hsa-miR-508-3p  hsa-miR-5089-3p  hsa-miR-5091  hsa-miR-510-3p  hsa-miR-513a-5p  hsa-miR-513c-5p  hsa-miR-514b-3p  hsa-miR-516b-5p  hsa-miR-5187-3p  hsa-miR-5188  hsa-miR-518d-3p  hsa-miR-5192  hsa-miR-5195-3p  hsa-miR-5196-3p  hsa-miR-5196-5p  hsa-miR-519d-3p  hsa-miR-520a-3p  hsa-miR-520b-3p  hsa-miR-520f-3p  hsa-miR-520g-3p  hsa-miR-520h  hsa-miR-521  hsa-miR-522-3p  hsa-miR-523-5p  hsa-miR-524-3p  hsa-miR-525-3p  hsa-miR-525-5p  hsa-miR-526b-5p  hsa-miR-532-5p  hsa-miR-539-3p  hsa-miR-542-3p  hsa-miR-544b  hsa-miR-548a-5p  hsa-miR-548al  hsa-miR-548an  hsa-miR-548ao-5p  hsa-miR-548bb-3p  hsa-miR-548h-3p  hsa-miR-548h-5p  hsa-miR-548j-5p  hsa-miR-548t-5p  hsa-miR-548x-3p  hsa-miR-548y  hsa-miR-548z  hsa-miR-551a  hsa-miR-5572  hsa-miR-5580-3p  hsa-miR-5586-5p  hsa-miR-5587-5p  hsa-miR-561-3p  hsa-miR-5680  hsa-miR-5681b  hsa-miR-5684  hsa-miR-5689  hsa-miR-5690  hsa-miR-5692b  hsa-miR-5693  hsa-miR-5695  hsa-miR-5697  hsa-miR-5701  hsa-miR-571  hsa-miR-572  hsa-miR-573  hsa-miR-574-3p  hsa-miR-574-5p  hsa-miR-579-5p  hsa-miR-580-3p  hsa-miR-582-3p  hsa-miR-586  hsa-miR-588  hsa-miR-589-5p  hsa-miR-590-3p  hsa-miR-598-3p  hsa-miR-598-5p  hsa-miR-600  hsa-miR-6071  hsa-miR-6073  hsa-miR-6075  hsa-miR-6080  hsa-miR-6082  hsa-miR-6090  hsa-miR-611  hsa-miR-6126  hsa-miR-613  hsa-miR-615-3p  hsa-miR-615-5p  hsa-miR-6165  hsa-miR-618  hsa-miR-619-3p  hsa-miR-619-5p  hsa-miR-625-3p  hsa-miR-627-5p  hsa-miR-629-5p  hsa-miR-635  hsa-miR-639  hsa-miR-641  hsa-miR-642a-3p  hsa-miR-643  hsa-miR-6499-3p  hsa-miR-6502-3p  hsa-miR-6503-3p  hsa-miR-6504-5p  hsa-miR-6510-5p  hsa-miR-6513-5p  hsa-miR-6515-5p  hsa-miR-6516-3p  hsa-miR-6516-5p  hsa-miR-652-3p  hsa-miR-653-5p  hsa-miR-654-3p  hsa-miR-660-5p  hsa-miR-663b  hsa-miR-665  hsa-miR-670-3p  hsa-miR-671-5p  hsa-miR-6715a-3p  hsa-miR-6715b-3p  hsa-miR-6724-5p  hsa-miR-6728-3p  hsa-miR-6729-3p  hsa-miR-6729-5p  hsa-miR-6732-3p  hsa-miR-6734-3p  hsa-miR-6739-3p  hsa-miR-6739-5p  hsa-miR-6740-3p  hsa-miR-6741-3p  hsa-miR-6743-5p  hsa-miR-6744-3p  hsa-miR-6745  hsa-miR-6749-3p  hsa-miR-6749-5p  hsa-miR-6751-3p  hsa-miR-6751-5p  hsa-miR-6752-3p  hsa-miR-6752-5p  hsa-miR-6754-3p  hsa-miR-6755-3p  hsa-miR-6755-5p  hsa-miR-6758-5p  hsa-miR-6760-3p  hsa-miR-6764-5p  hsa-miR-6769b-5p  hsa-miR-6770-5p  hsa-miR-6771-3p  hsa-miR-6771-5p  hsa-miR-6773-3p  hsa-miR-6775-5p  hsa-miR-6776-3p  hsa-miR-6777-5p  hsa-miR-6780b-3p  hsa-miR-6781-3p  hsa-miR-6781-5p  hsa-miR-6785-5p  hsa-miR-6787-5p  hsa-miR-6789-5p  hsa-miR-6790-5p  hsa-miR-6793-3p  hsa-miR-6794-3p  hsa-miR-6795-5p  hsa-miR-6797-3p  hsa-miR-6798-3p  hsa-miR-6800-3p  hsa-miR-6801-3p  hsa-miR-6803-3p  hsa-miR-6803-5p  hsa-miR-6804-3p  hsa-miR-6804-5p  hsa-miR-6805-3p  hsa-miR-6806-3p  hsa-miR-6808-3p  hsa-miR-6821-3p  hsa-miR-6822-3p  hsa-miR-6823-5p  hsa-miR-6826-3p  hsa-miR-6827-5p  hsa-miR-6832-3p  hsa-miR-6836-5p  hsa-miR-6845-3p  hsa-miR-6847-5p  hsa-miR-6848-3p  hsa-miR-6849-3p  hsa-miR-6854-3p  hsa-miR-6856-5p  hsa-miR-6864-3p  hsa-miR-6866-3p  hsa-miR-6867-3p  hsa-miR-6868-3p  hsa-miR-6868-5p  hsa-miR-6869-3p  hsa-miR-6870-5p  hsa-miR-6872-5p  hsa-miR-6873-3p  hsa-miR-6874-3p  hsa-miR-6880-3p  hsa-miR-6881-3p  hsa-miR-6883-3p  hsa-miR-6885-3p  hsa-miR-6886-3p  hsa-miR-6888-5p  hsa-miR-6891-5p  hsa-miR-6892-5p  hsa-miR-6893-5p  hsa-miR-7-2-3p  hsa-miR-7-5p  hsa-miR-708-3p  hsa-miR-708-5p  hsa-miR-7106-3p  hsa-miR-7107-3p  hsa-miR-7107-5p  hsa-miR-7108-3p  hsa-miR-7108-5p  hsa-miR-7111-3p  hsa-miR-7112-5p  hsa-miR-7113-5p  hsa-miR-7155-5p  hsa-miR-7157-3p  hsa-miR-7158-3p  hsa-miR-7158-5p  hsa-miR-7161-5p  hsa-miR-718  hsa-miR-758-3p  hsa-miR-759  hsa-miR-765  hsa-miR-769-5p  hsa-miR-7702  hsa-miR-7704  hsa-miR-7852-3p  hsa-miR-7853-5p  hsa-miR-7855-5p  hsa-miR-7974  hsa-miR-8053  hsa-miR-8054  hsa-miR-8058  hsa-miR-8060  hsa-miR-8065  hsa-miR-8067  hsa-miR-8070  hsa-miR-8074  hsa-miR-8084  hsa-miR-8085  hsa-miR-8088  hsa-miR-8485  hsa-miR-874-5p  hsa-miR-875-3p  hsa-miR-885-5p  hsa-miR-887-3p  hsa-miR-887-5p  hsa-miR-889-3p  hsa-miR-892a  hsa-miR-892c-3p  hsa-miR-922  hsa-miR-92a-2-5p  hsa-miR-92a-3p  hsa-miR-93-3p  hsa-miR-93-5p  hsa-miR-934  hsa-miR-937-3p  hsa-miR-940  hsa-miR-944  hsa-miR-98-3p  hsa-miR-98-5p  hsa-miR-9851-5p  hsa-miR-9898  hsa-miR-9899  hsa-miR-9900  hsa-miR-9901  hsa-miR-9985  hsa-miR-99a-5p  hsa-miR-99b-3p  hsa-miR-99b-5p | 1.9073  1.5494  1.2771  1.0758  1.0552  1.9329  1.1006  3.0155  2.4900  3.3639  2.0971  2.5788  1.1185  1.1413  1.6545  -2.3398  2.3127  1.4548  -1.1070  -1.1154  -1.8067  1.5483  2.8563  2.2056  1.4810  3.6312  -1.5926  1.5406  2.4792  -1.5535  -1.8220  2.2487  -1.2712  1.8234  2.6961  -1.2118  -1.1904  3.8432  -2.3738  1.9451  -1.8741  1.3849  -2.0892  2.2385  2.8916  -1.8769  2.7680  -2.4105  3.3222  1.0961  1.8292  2.6394  2.7018  1.7381  2.3193  3.2417  2.7400  2.3767  4.0983  -2.3528  2.2703  -1.4630  1.1811  1.6924  1.8103  -1.4132  -3.6554  1.3687  -2.8882  2.1413  1.1715  3.4057  2.6715  2.2255  2.4394  -1.6704  2.1561  -1.6976  1.7309  -2.1029  -1.5508  -3.6620  3.2835  3.1012  -1.3396  1.8216  2.8128  1.2128  1.6304  -2.1001  3.2227  2.1799  1.2294  2.6571  8.7390  1.0717  -1.8468  3.6635  2.2986  -1.7945  2.0057  1.2859  2.5225  2.9486  1.4716  1.7295  2.1710  1.5604  2.3579  2.6982  1.4988  2.0203  2.7168  2.4218  2.8558  2.8949  -3.6821  3.9841  -1.1558  2.4912  3.2456  4.4245  3.5670  -4.3927  2.0877  -2.3508  2.4879  1.6522  2.6791  -1.5641  1.9846  2.7090  3.2945  7.1312  5.3009  -1.4572  3.4882  3.4929  3.5034  3.9448  2.5959  2.3935  3.9876  2.4318  3.8687  1.7022  -1.5786  -1.9216  -2.0789  1.3941  3.1522  3.6191  2.7462  3.5396  -1.8192  5.7935  1.2225  -2.5483  2.3526  1.8110  2.2295  2.7540  1.9902  3.9131  -2.1440  -2.3446  2.2323  2.3246  2.0562  -1.3623  2.8407  1.9884  2.8521  2.7296  1.4409  2.9603  2.2281  1.4114  -2.5739  1.4517  1.5013  2.9795  1.5224  1.9659  2.5909  1.4483  -1.8262  -3.4458  -2.4856  -4.5967  2.6927  2.5039  -1.5745  2.2613  1.6001  1.4589  3.1705  2.2177  1.5871  3.1968  1.5386  -3.2592  -1.9862  -1.1607  -1.6715  -2.4679  3.2408  1.1539  -1.3415  -1.3771  -2.3230  -1.1884  -2.5029  -1.8530  -1.1810  1.0137  2.4830  1.4386  -2.2964  2.8379  1.3809  -1.3904  1.5884  1.5476  1.4422  1.9485  1.6567  -3.0579  1.3229  1.7730  1.9907  1.8231  1.9099  2.5924  1.8212  4.0882  -1.7675  1.5937  1.0487  -1.5530  1.2563  1.8054  -2.1253  1.0854  -3.6755  4.6029  -1.5708  -1.0144  -2.0389  -1.8283  3.2298  2.7126  1.5622  -2.0667  -1.2346  -2.8224  -3.6822  -2.7378  -1.7504  -1.2852  3.1598  -1.3945  -1.9674  2.5668  2.4761  -1.8235  1.4524  1.6463  4.5614  2.7745  2.2670  2.4715  2.9126  2.3647  4.5671  3.4624  3.0290  1.6829  1.1369  -1.7865  -2.3662  1.9835  1.0015  3.0498  2.4605  1.0812  2.0543  1.0312  -1.8126  -3.6859  -1.8357  -1.5798  -2.6316  2.7869  1.3524  -1.1942  4.4144  1.6878  -2.1672  1.4378  2.6916  -1.6561  2.2687  1.8222  1.2653  3.3753  3.0811  1.3315  1.9234  -2.6661  2.3703  -1.9070  1.7476  1.3282  -2.6174  -2.0561  -1.6164  -2.1952  -1.2540  2.5019  -2.7197  -2.0455  1.1157  3.6356  2.8998  -1.6314  1.5100  2.7446  2.2562  4.0260  -2.8636  -2.9231  3.6107  1.1596  1.8593  -1.6675  -1.4257  -1.2148  -1.3557  -2.4362  -2.2561  -1.5839  -1.5137  1.5325  2.0879  -1.0767  1.6949  -2.2109  1.1967  4.5483  3.0937  2.7603  1.2174  2.1250  2.5052  2.2853  -2.8941  1.6156  1.9418  1.7127  3.3462  1.2971  -1.7565  -1.9210  -1.1169  -2.2895  -1.0855  1.7883  -1.1879  1.0193  2.9154  -1.3424  1.6148  -3.7344  2.0964  -1.3451  -5.7451  -1.4789  -2.0251  1.8279  -3.1284  -1.3919  -1.5112  -2.2507  -2.7111  2.7541  -2.1147  -1.5700  -2.4014  1.7702  -1.2493  1.1496  -2.6354  -1.4458  -2.3719  1.5727  1.3607  -1.9894  2.1298  1.1963  -1.9385  1.4638  1.0486  -2.3675  -2.2101  -1.9189  -1.9023  -3.0918  2.1124  -1.7967  -2.8208  1.4550  -1.8381  -2.3213  1.7544  1.4039  1.9047  -1.8111  1.2867  -1.9220  -1.0925  2.2479  -1.3531  3.3622  2.7795  2.2886  2.3184  3.3029  -2.3251  2.9603  -2.7851  1.5446  2.0766  1.9971  -1.5565  1.8834  1.4624  2.4760  3.4903  -1.1662  1.2864  -1.8036  3.9639  -2.2254  -2.4613  2.4567  2.7062  2.4692  -1.8080  -1.2425  2.3037  -2.1234  -1.9154  -1.5456  2.5578  -1.1407  -3.7810  -3.4300  -1.6236  -2.1389  -2.3916  -2.1596  -2.0067  -1.4472  -1.6877  3.6285  -1.7586  4.0110  1.6797  1.0471  -2.1621  -1.7703  -2.8995  -2.2031  -2.1788  -2.5352  1.8300  1.4250  2.6193  -1.6355  -2.5322  -1.5610  2.8122  -1.2348  -3.2743  -2.0654  -1.2979  -2.7706  -2.6950  -1.4688  -2.7502  1.7324  1.8359  -1.9688  -4.2350  -3.0743  -2.2684  -2.7005  -1.6252  2.9461  1.6567  -2.1238  1.3630  1.6759  -1.7390  2.9925  2.2908  -2.7280  -1.9691  4.3213  2.6739  1.7620  -2.0862  -3.0347  1.8461  3.3771  1.2840  3.0244  1.3502  -1.2870  1.5251  1.8125  -1.8793  2.6488  -1.4782  -1.8679  1.2709  -2.3260  1.8552  1.0669  4.0800  2.3543  -1.4965  -1.8154  -2.0262  -2.5845  2.3646  -2.1424  -4.5946  3.0056  3.2256  -1.1020  1.7187  -1.7484  2.3676  -1.1554  1.7568  3.0220  2.5837  4.1717  1.1504  1.2585  -1.7167  1.0164  3.6285  -2.0224  2.5445  1.9895  -1.3795  1.0551  -1.1064  -1.1858  -2.3619  -1.3496  1.2025  1.2935  -1.2407  -1.6764  -1.9264  1.5396  -1.5568  -1.5927  -2.6346  -1.5018  -1.4122  -1.3625  -1.9685  2.1258  -1.0675  -1.0137  1.9254  2.3868  2.0074  1.0524  1.2330  4.2163  -1.2503  -2.0617  -1.0052  -1.0479  1.7135  -1.2706  -1.0614  -1.0954  1.8119  -1.4079  -2.5420  -1.5818  -1.8473  3.9240  1.3488  -1.8889  -1.5042  2.2473  -1.4190  -1.9231  -2.5882  1.2380  1.2565  1.8684  -1.9562  -1.8208  -2.3391  4.2292  -2.1557  -1.4326  2.9725  1.3153  -1.3444  3.6331  -2.1589  -1.7399  -1.5645  2.7363  -2.7306  -1.9402  -2.6324  -2.3113  1.7741  -1.4872  -2.8018  -3.7634  -2.2224  1.4978  -1.7181  -1.5190  -1.9984  1.9540  -1.6267  -3.0045  -1.7481  1.7571  -1.5680  2.8283  1.9246  3.4590  1.5952  -1.0633  -1.1717  -1.3548  2.4654  4.2059  6.8930  -1.4676  -1.3168  -1.5653  -2.9408  1.6682  -1.5892  1.2435  1.4706  2.2643  1.7882  1.4045  -1.2852  3.6806  -2.3851  -1.6321  1.5683  2.0999  -1.9191  -1.3267  -1.5689  -1.3889  -2.1832  -4.3688  -2.1436  -1.6556  -1.9114  -1.5044  -1.6577  1.4777  1.0662  4.3540  -1.2913  1.0007  4.0651  2.4845  -2.5194  -2.3029  -1.0742  -2.4457  1.4460  1.6966  2.8082  -2.2243  -1.3922  1.4890  -2.0716  3.5039  1.5007  -1.5419  -1.6807  3.0148  -1.6217  2.8760  1.7147  1.8867  2.3472  1.8647 | 0.0000  0.0000  0.0000  0.0061  0.0000  0.0000  0.0290  0.0000  0.0000  0.0000  0.0000  0.0000  0.0012  0.0007  0.0000  0.0028  0.0000  0.0000  0.0002  0.0433  0.0002  0.0106  0.0000  0.0000  0.0000  0.0000  0.0110  0.0090  0.0381  0.0217  0.0140  0.0000  0.0024  0.0002  0.0000  0.0491  0.0002  0.0000  0.0013  0.0044  0.0056  0.0401  0.0000  0.0293  0.0000  0.0047  0.0000  0.0004  0.0000  0.0017  0.0000  0.0000  0.0000  0.0484  0.0000  0.0000  0.0000  0.0000  0.0142  0.0006  0.0000  0.0233  0.0000  0.0000  0.0000  0.0328  0.0000  0.0368  0.0031  0.0142  0.0033  0.0000  0.0000  0.0000  0.0000  0.0000  0.0001  0.0124  0.0000  0.0317  0.0004  0.0001  0.0000  0.0000  0.0332  0.0000  0.0000  0.0400  0.0487  0.0298  0.0000  0.0000  0.0002  0.0000  0.0000  0.0000  0.0456  0.0000  0.0000  0.0003  0.0000  0.0028  0.0000  0.0000  0.0000  0.0155  0.0001  0.0009  0.0000  0.0000  0.0187  0.0000  0.0000  0.0000  0.0005  0.0000  0.0001  0.0000  0.0225  0.0000  0.0000  0.0045  0.0000  0.0000  0.0000  0.0009  0.0000  0.0479  0.0000  0.0255  0.0000  0.0000  0.0000  0.0000  0.0000  0.0254  0.0000  0.0000  0.0000  0.0000  0.0000  0.0000  0.0000  0.0000  0.0212  0.0049  0.0145  0.0001  0.0119  0.0055  0.0000  0.0330  0.0000  0.0000  0.0261  0.0000  0.0350  0.0013  0.0000  0.0000  0.0000  0.0000  0.0000  0.0000  0.0060  0.0024  0.0000  0.0000  0.0000  0.0321  0.0000  0.0000  0.0000  0.0000  0.0005  0.0000  0.0000  0.0069  0.0403  0.0001  0.0007  0.0000  0.0000  0.0000  0.0002  0.0293  0.0039  0.0009  0.0061  0.0013  0.0000  0.0000  0.0340  0.0039  0.0000  0.0000  0.0000  0.0000  0.0139  0.0000  0.0041  0.0072  0.0238  0.0028  0.0113  0.0076  0.0000  0.0022  0.0166  0.0000  0.0008  0.0243  0.0003  0.0362  0.0331  0.0460  0.0000  0.0132  0.0120  0.0000  0.0118  0.0002  0.0000  0.0000  0.0000  0.0000  0.0018  0.0002  0.0387  0.0004  0.0000  0.0071  0.0000  0.0000  0.0000  0.0000  0.0089  0.0135  0.0001  0.0344  0.0000  0.0000  0.0153  0.0083  0.0015  0.0000  0.0188  0.0277  0.0000  0.0341  0.0000  0.0000  0.0083  0.0167  0.0019  0.0100  0.0005  0.0049  0.0171  0.0286  0.0000  0.0457  0.0118  0.0000  0.0000  0.0348  0.0095  0.0008  0.0037  0.0006  0.0019  0.0344  0.0000  0.0000  0.0029  0.0000  0.0074  0.0109  0.0421  0.0261  0.0086  0.0000  0.0421  0.0000  0.0000  0.0000  0.0240  0.0309  0.0180  0.0014  0.0191  0.0001  0.0017  0.0000  0.0000  0.0101  0.0055  0.0000  0.0372  0.0000  0.0000  0.0070  0.0009  0.0000  0.0000  0.0000  0.0000  0.0095  0.0000  0.0378  0.0456  0.0445  0.0000  0.0004  0.0308  0.0173  0.0323  0.0289  0.0148  0.0000  0.0107  0.0059  0.0003  0.0305  0.0000  0.0384  0.0000  0.0000  0.0036  0.0000  0.0009  0.0049  0.0356  0.0000  0.0000  0.0198  0.0496  0.0183  0.0027  0.0029  0.0029  0.0281  0.0049  0.0446  0.0002  0.0314  0.0238  0.0069  0.0085  0.0000  0.0001  0.0000  0.0142  0.0190  0.0004  0.0000  0.0026  0.0081  0.0002  0.0001  0.0000  0.0001  0.0157  0.0380  0.0131  0.0425  0.0153  0.0264  0.0207  0.0125  0.0000  0.0276  0.0001  0.0152  0.0490  0.0318  0.0001  0.0193  0.0260  0.0357  0.0007  0.0162  0.0376  0.0086  0.0001  0.0000  0.0152  0.0060  0.0206  0.0059  0.0246  0.0010  0.0026  0.0060  0.0018  0.0090  0.0000  0.0042  0.0000  0.0047  0.0090  0.0030  0.0020  0.0092  0.0076  0.0013  0.0088  0.0108  0.0088  0.0055  0.0077  0.0000  0.0246  0.0051  0.0000  0.0127  0.0000  0.0064  0.0006  0.0107  0.0049  0.0003  0.0379  0.0000  0.0000  0.0000  0.0000  0.0021  0.0050  0.0000  0.0002  0.0073  0.0000  0.0045  0.0077  0.0014  0.0149  0.0000  0.0000  0.0330  0.0333  0.0286  0.0000  0.0003  0.0129  0.0005  0.0001  0.0000  0.0225  0.0010  0.0000  0.0061  0.0000  0.0001  0.0000  0.0189  0.0142  0.0295  0.0035  0.0001  0.0033  0.0003  0.0419  0.0013  0.0003  0.0294  0.0196  0.0000  0.0050  0.0017  0.0066  0.0420  0.0240  0.0000  0.0272  0.0217  0.0379  0.0289  0.0012  0.0233  0.0257  0.0430  0.0032  0.0240  0.0000  0.0130  0.0401  0.0078  0.0022  0.0393  0.0155  0.0317  0.0378  0.0122  0.0050  0.0016  0.0006  0.0012  0.0313  0.0071  0.0000  0.0002  0.0000  0.0000  0.0018  0.0009  0.0008  0.0409  0.0073  0.0000  0.0000  0.0001  0.0006  0.0023  0.0033  0.0000  0.0080  0.0000  0.0002  0.0006  0.0001  0.0000  0.0368  0.0000  0.0044  0.0079  0.0008  0.0129  0.0001  0.0002  0.0144  0.0000  0.0015  0.0183  0.0034  0.0394  0.0000  0.0327  0.0013  0.0000  0.0000  0.0225  0.0000  0.0011  0.0478  0.0185  0.0006  0.0000  0.0000  0.0000  0.0000  0.0013  0.0453  0.0008  0.0294  0.0074  0.0000  0.0209  0.0005  0.0358  0.0274  0.0234  0.0005  0.0001  0.0335  0.0000  0.0058  0.0244  0.0112  0.0016  0.0002  0.0000  0.0009  0.0043  0.0012  0.0013  0.0330  0.0043  0.0122  0.0038  0.0002  0.0000  0.0471  0.0051  0.0040  0.0000  0.0207  0.0001  0.0153  0.0197  0.0000  0.0464  0.0342  0.0008  0.0000  0.0069  0.0212  0.0009  0.0136  0.0000  0.0000  0.0002  0.0150  0.0000  0.0007  0.0149  0.0295  0.0000  0.0003  0.0034  0.0038  0.0406  0.0002  0.0000  0.0208  0.0001  0.0000  0.0144  0.0446  0.0305  0.0021  0.0180  0.0259  0.0000  0.0016  0.0032  0.0021  0.0004  0.0192  0.0178  0.0230  0.0000  0.0051  0.0000  0.0018  0.0009  0.0076  0.0000  0.0000  0.0001  0.0023  0.0001  0.0265  0.0000  0.0009  0.0000  0.0001  0.0220  0.0030  0.0003  0.0000  0.0000  0.0000  0.0102  0.0008  0.0428  0.0172  0.0072  0.0092  0.0000  0.0249  0.0003  0.0000  0.0000  0.0122  0.0000  0.0414  0.0316  0.0001  0.0000  0.0066  0.0379  0.0488  0.0150  0.0038  0.0025  0.0022  0.0108  0.0059  0.0084  0.0290  0.0406  0.0126  0.0000  0.0177  0.0001  0.0140  0.0000  0.0487  0.0123  0.0402  0.0000  0.0000  0.0063  0.0000  0.0359  0.0007  0.0004  0.0243  0.0000  0.0000  0.0190  0.0188  0.0000  0.0197  0.0000  0.0043  0.0000  0.0000  0.0000 | 0.0000  0.0000  0.0000  0.0345  0.0001  0.0000  0.1171  0.0000  0.0000  0.0000  0.0000  0.0000  0.0088  0.0057  0.0001  0.0184  0.0000  0.0000  0.0020  0.1564  0.0017  0.0540  0.0000  0.0000  0.0002  0.0000  0.0556  0.0471  0.1418  0.0947  0.0675  0.0000  0.0158  0.0022  0.0004  0.1731  0.0015  0.0000  0.0097  0.0270  0.0325  0.1481  0.0000  0.1180  0.0000  0.0285  0.0000  0.0034  0.0000  0.0119  0.0000  0.0000  0.0000  0.1716  0.0000  0.0000  0.0000  0.0001  0.0679  0.0048  0.0000  0.1001  0.0000  0.0000  0.0000  0.1280  0.0003  0.1391  0.0199  0.0679  0.0210  0.0000  0.0000  0.0000  0.0000  0.0000  0.0007  0.0611  0.0000  0.1249  0.0034  0.0012  0.0000  0.0000  0.1286  0.0000  0.0000  0.1479  0.1722  0.1191  0.0000  0.0000  0.0018  0.0000  0.0000  0.0001  0.1635  0.0000  0.0000  0.0023  0.0000  0.0184  0.0000  0.0000  0.0000  0.0721  0.0007  0.0071  0.0000  0.0000  0.0843  0.0000  0.0000  0.0000  0.0041  0.0000  0.0014  0.0000  0.0972  0.0000  0.0000  0.0271  0.0000  0.0001  0.0000  0.0071  0.0000  0.1700  0.0000  0.1063  0.0002  0.0000  0.0000  0.0000  0.0000  0.1063  0.0000  0.0000  0.0000  0.0000  0.0000  0.0001  0.0000  0.0000  0.0927  0.0291  0.0688  0.0012  0.0595  0.0321  0.0000  0.1282  0.0000  0.0000  0.1082  0.0000  0.1339  0.0094  0.0000  0.0000  0.0000  0.0000  0.0000  0.0000  0.0342  0.0160  0.0000  0.0000  0.0000  0.1258  0.0000  0.0000  0.0000  0.0000  0.0043  0.0000  0.0000  0.0386  0.1482  0.0007  0.0058  0.0000  0.0000  0.0000  0.0019  0.1180  0.0242  0.0070  0.0347  0.0092  0.0000  0.0000  0.1313  0.0243  0.0000  0.0000  0.0000  0.0000  0.0671  0.0000  0.0252  0.0395  0.1016  0.0186  0.0570  0.0414  0.0000  0.0150  0.0766  0.0000  0.0061  0.1029  0.0027  0.1371  0.1283  0.1643  0.0000  0.0639  0.0598  0.0000  0.0592  0.0021  0.0000  0.0000  0.0001  0.0000  0.0123  0.0022  0.1439  0.0033  0.0000  0.0392  0.0000  0.0000  0.0004  0.0000  0.0467  0.0657  0.0012  0.1319  0.0003  0.0000  0.0712  0.0444  0.0104  0.0000  0.0847  0.1130  0.0000  0.1313  0.0000  0.0000  0.0444  0.0770  0.0132  0.0515  0.0042  0.0291  0.0787  0.1163  0.0001  0.1635  0.0593  0.0000  0.0000  0.1332  0.0488  0.0066  0.0232  0.0048  0.0135  0.1319  0.0000  0.0000  0.0191  0.0000  0.0403  0.0553  0.1530  0.1082  0.0456  0.0000  0.1530  0.0000  0.0000  0.0000  0.1018  0.1228  0.0817  0.0101  0.0855  0.0010  0.0119  0.0000  0.0000  0.0517  0.0318  0.0000  0.1403  0.0000  0.0000  0.0390  0.0070  0.0000  0.0000  0.0000  0.0000  0.0492  0.0000  0.1417  0.1635  0.1605  0.0000  0.0038  0.1224  0.0792  0.1266  0.1171  0.0699  0.0000  0.0546  0.0339  0.0025  0.1216  0.0000  0.1429  0.0000  0.0000  0.0225  0.0000  0.0070  0.0291  0.1359  0.0000  0.0000  0.0877  0.1741  0.0828  0.0175  0.0191  0.0189  0.1144  0.0291  0.1606  0.0015  0.1240  0.1016  0.0384  0.0449  0.0000  0.0010  0.0001  0.0679  0.0849  0.0034  0.0000  0.0175  0.0432  0.0016  0.0014  0.0000  0.0014  0.0726  0.1417  0.0637  0.1543  0.0712  0.1091  0.0910  0.0615  0.0000  0.1130  0.0006  0.0712  0.1727  0.1250  0.0008  0.0859  0.1081  0.1360  0.0055  0.0748  0.1416  0.0454  0.0006  0.0000  0.0712  0.0341  0.0907  0.0339  0.1035  0.0078  0.0175  0.0341  0.0128  0.0469  0.0001  0.0258  0.0000  0.0285  0.0469  0.0192  0.0137  0.0480  0.0414  0.0092  0.0464  0.0547  0.0464  0.0317  0.0414  0.0001  0.1035  0.0301  0.0000  0.0626  0.0000  0.0359  0.0048  0.0544  0.0291  0.0026  0.1417  0.0000  0.0000  0.0000  0.0000  0.0143  0.0296  0.0000  0.0019  0.0400  0.0001  0.0273  0.0416  0.0104  0.0703  0.0000  0.0000  0.1282  0.1290  0.1162  0.0000  0.0023  0.0631  0.0038  0.0012  0.0000  0.0972  0.0073  0.0000  0.0344  0.0000  0.0014  0.0000  0.0849  0.0679  0.1182  0.0223  0.0014  0.0209  0.0029  0.1530  0.0096  0.0028  0.1180  0.0869  0.0000  0.0292  0.0122  0.0369  0.1530  0.1018  0.0000  0.1121  0.0947  0.1417  0.1171  0.0088  0.1002  0.1072  0.1558  0.0206  0.1018  0.0000  0.0634  0.1480  0.0421  0.0150  0.1458  0.0720  0.1249  0.1417  0.0607  0.0294  0.0111  0.0049  0.0089  0.1240  0.0395  0.0003  0.0017  0.0000  0.0000  0.0128  0.0071  0.0061  0.1498  0.0401  0.0000  0.0000  0.0007  0.0048  0.0153  0.0210  0.0000  0.0430  0.0000  0.0018  0.0049  0.0008  0.0000  0.1390  0.0000  0.0270  0.0423  0.0060  0.0631  0.0005  0.0019  0.0685  0.0000  0.0110  0.0828  0.0217  0.1460  0.0000  0.1279  0.0092  0.0000  0.0004  0.0972  0.0001  0.0080  0.1700  0.0836  0.0049  0.0000  0.0000  0.0000  0.0000  0.0094  0.1628  0.0060  0.1180  0.0406  0.0000  0.0915  0.0039  0.1362  0.1125  0.1004  0.0041  0.0007  0.1293  0.0000  0.0337  0.1029  0.0567  0.0111  0.0016  0.0000  0.0070  0.0263  0.0086  0.0096  0.1282  0.0262  0.0606  0.0235  0.0017  0.0000  0.1676  0.0301  0.0247  0.0000  0.0910  0.0008  0.0712  0.0872  0.0000  0.1658  0.1314  0.0060  0.0000  0.0386  0.0928  0.0070  0.0660  0.0000  0.0004  0.0016  0.0703  0.0000  0.0058  0.0702  0.1182  0.0000  0.0027  0.0213  0.0234  0.1489  0.0019  0.0000  0.0912  0.0009  0.0000  0.0687  0.1606  0.1216  0.0144  0.0817  0.1078  0.0000  0.0112  0.0204  0.0144  0.0034  0.0857  0.0814  0.0990  0.0000  0.0301  0.0005  0.0123  0.0067  0.0413  0.0000  0.0000  0.0009  0.0153  0.0011  0.1092  0.0000  0.0067  0.0000  0.0012  0.0957  0.0195  0.0027  0.0000  0.0000  0.0000  0.0521  0.0066  0.1554  0.0788  0.0396  0.0480  0.0000  0.1042  0.0027  0.0000  0.0000  0.0606  0.0000  0.1516  0.1246  0.0010  0.0000  0.0372  0.1417  0.1724  0.0703  0.0238  0.0168  0.0150  0.0547  0.0338  0.0448  0.1171  0.1489  0.0621  0.0000  0.0809  0.0007  0.0675  0.0000  0.1722  0.0607  0.1482  0.0004  0.0000  0.0358  0.0000  0.1362  0.0059  0.0036  0.1029  0.0000  0.0000  0.0849  0.0848  0.0000  0.0872  0.0000  0.0263  0.0000  0.0000  0.0000 |

*Data were analyzed by miRNA sequencing.
