# Supplementary material for: Mesenchymal Stem Cells-Derived Exosomes Alleviate Acute Lung Injury by Inhibiting Alveolar Macrophage Pyroptosis
Source: Stem Cells Transl Med. 2024 Feb 13;13(4):371–86. doi: 10.1093/stcltm/szad094 (PMC11016849; doi:10.1093/stcltm/szad094)
Supplement: szad094_suppl_Supplementary_Materials [file szad094_suppl_supplementary_materials.zip › szad094_suppl_Supplementary_Tables_S2.docx]

**Table S2. Pyroptosis Signaling Pathway.**

| **Symbol** | **Entrez gene name** | **Location** | **Gene ID** |
| --- | --- | --- | --- |
| AIM2  BAX  CASP1  CASP3  CASP4  CASP5  CASP8  CASP9  DHX9  ELAVL1  FOXO3  GAS5  GBP1  GBP2  GBP3  GBP4  GBP5  GBP7  GSDMB  GSDMD  GSDME  GZMA  HMGB1  IL18  IL1A  IL1B  IL1R1  IRF2  KCNQ1OT1  MAPK1  MAPK11  MAPK12  MAPK13  MAPK14  MEFV  NAIP  NEK7  NFKB1  NFKB2  NGFR  NLRC4  NLRP1  NLRP10  NLRP11  NLRP12  NLRP13  NLRP14  NLRP2  NLRP3  NLRP4  NLRP5  NLRP6  NLRP7  NLRP8  NLRP9  NOL3  P2RX7  PANX1  PRKACA  PRKACB  PRKACG  PRKAG1  PRKAG2  PRKAR1A  PRKAR1B  PRKAR2A  PRKAR2B  PTGER4  PYCARD  RHOA  TLR1  TLR10  TLR2  TLR3  TLR4  TLR5  TLR6  TLR7  TLR8  TLR9  TNF  TNFRSF11B  TNFRSF1A  TNFRSF1B  TRAF3  TXNIP | absent in melanoma 2  BCL2 associated X, apoptosis regulator  caspase 1  caspase 3  caspase 4  caspase 5  caspase 8  caspase 9  DExH-box helicase 9  ELAV like RNA binding protein 1  forkhead box O3  growth arrest specific 5  guanylate binding protein 1  guanylate binding protein 2  guanylate binding protein 3  guanylate binding protein 4  guanylate binding protein 5  guanylate binding protein 7  gasdermin B  gasdermin D  gasdermin E  granzyme A  high mobility group box 1  interleukin 18  interleukin 1 alpha  interleukin 1 beta  interleukin 1 receptor type 1  interferon regulatory factor 2  KCNQ1 opposite strand/antisense transcript 1  mitogen-activated protein kinase 1  mitogen-activated protein kinase 11  mitogen-activated protein kinase 12  mitogen-activated protein kinase 13  mitogen-activated protein kinase 14  MEFV innate immuity regulator, pyrin  NLR family apoptosis inhibitory protein  NIMA related kinase 7  nuclear factor kappa B subunit 1  nuclear factor kappa B subunit 2  nerve growth factor receptor  NLR family CARD domain containing 4  NLR family pyrin domain containing 1  NLR family pyrin domain containing 10  NLR family pyrin domain containing 11  NLR family pyrin domain containing 12  NLR family pyrin domain containing 13  NLR family pyrin domain containing 14  NLR family pyrin domain containing 2  NLR family pyrin domain containing 3  NLR family pyrin domain containing 4  NLR family pyrin domain containing 5  NLR family pyrin domain containing 6  NLR family pyrin domain containing 7  NLR family pyrin domain containing 8  NLR family pyrin domain containing 9  nucleolar protein 3  purinergic receptor P2X 7  pannexin 1  protein kinase cAMP-activated catalytic subunit alpha  protein kinase cAMP-activated catalytic subunit beta  protein kinase cAMP-activated catalytic subunit gamma  protein kinase AMP-activated non-catalytic subunit gamma 1  protein kinase AMP-activated non-catalytic subunit gamma 2  protein kinase cAMP-dependent type I regulatory subunit alpha  protein kinase cAMP-dependent type I regulatory subunit beta  protein kinase cAMP-dependent type II regulatory subunit alpha  protein kinase cAMP-dependent type II regulatory subunit beta  prostaglandin E receptor 4  PYD and CARD domain containing  ras homolog family member A  toll like receptor 1  toll like receptor 10  toll like receptor 2  toll like receptor 3  toll like receptor 4  toll like receptor 5  toll like receptor 6  toll like receptor 7  toll like receptor 8  toll like receptor 9  tumor necrosis factor  TNF receptor superfamily member 11b  TNF receptor superfamily member 1A  TNF receptor superfamily member 1B  TNF receptor associated factor 3  thioredoxin interacting protein | Cytoplasm  Cytoplasm  Cytoplasm  Cytoplasm  Cytoplasm  Cytoplasm  Nucleus  Cytoplasm  Nucleus  Cytoplasm  Nucleus  Other  Cytoplasm  Cytoplasm  Cytoplasm  Cytoplasm  Plasma Membrane  Cytoplasm  Cytoplasm  Extracellular Space  Plasma Membrane  Cytoplasm  Nucleus  Extracellular Space  Extracellular Space  Extracellular Space  Plasma Membrane  Nucleus  Other  Cytoplasm  Cytoplasm  Cytoplasm  Cytoplasm  Cytoplasm  Nucleus  Cytoplasm  Nucleus  Nucleus  Nucleus  Plasma Membrane  Cytoplasm  Cytoplasm  Other  Extracellular Space  Cytoplasm  Other  Extracellular Space  Nucleus  Cytoplasm  Cytoplasm  Cytoplasm  Plasma Membrane  Extracellular Space  Other  Cytoplasm  Nucleus  Plasma Membrane  Plasma Membrane  Cytoplasm  Cytoplasm  Cytoplasm  Nucleus  Cytoplasm  Cytoplasm  Cytoplasm  Cytoplasm  Cytoplasm  Plasma Membrane  Cytoplasm  Cytoplasm  Plasma Membrane  Plasma Membrane  Plasma Membrane  Plasma Membrane  Plasma Membrane  Plasma Membrane  Plasma Membrane  Plasma Membrane  Plasma Membrane  Plasma Membrane  Extracellular Space  Plasma Membrane  Plasma Membrane  Plasma Membrane  Cytoplasm  Cytoplasm | 9447  581  834  836  837  838  841  842  1660  1994  2309  60674  2633  2634  2635  115361  115362  388646  55876  79792  1687  3001  3146  3606  3552  3553  3554  3660  10984  5594  5600  6300  5603  1432  4210  4671  140609  4790  4791  4804  58484  22861  338322  204801  91662  126204  338323  55655  114548  147945  126206  171389  199713  126205  338321  8996  5027  24145  5566  5567  5568  5571  51422  5573  5575  5576  5577  5734  29108  387  7096  81793  7097  7098  7099  7100  10333  51284  51311  54106  7124  4982  7132  7133  7187  10628 |

*Data were collected from the IPA software.
