# Supplementary material for: Mesenchymal Stem Cells-Derived Exosomes Alleviate Acute Lung Injury by Inhibiting Alveolar Macrophage Pyroptosis
Source: Stem Cells Transl Med. 2024 Feb 13;13(4):371–86. doi: 10.1093/stcltm/szad094 (PMC11016849; doi:10.1093/stcltm/szad094)
Supplement: szad094_suppl_Supplementary_Materials [file szad094_suppl_supplementary_materials.zip › szad094_suppl_Supplementary_Tables_S3.docx]

**Table S3. Upregulated miRNAs and target PRGs.**

| **MiRNA** | ***P*-value** | **Target-gene** | **Confidence** |
| --- | --- | --- | --- |
| hsa-miR-98-3p  hsa-let-7i-5p  hsa-let-7i-5p  hsa-let-7i-5p  hsa-let-7i-5p  hsa-let-7i-5p  hsa-let-7i-5p  hsa-let-7d-3p  hsa-let-7d-3p  hsa-let-7d-3p  hsa-miR-100-5p  hsa-miR-101-3p  hsa-miR-101-3p  hsa-miR-101-3p  hsa-miR-101-3p  hsa-miR-101-3p  hsa-miR-101-3p  hsa-miR-103a-3p  hsa-miR-103a-3p  hsa-miR-103a-3p  hsa-miR-103a-3p  hsa-miR-103a-3p  hsa-miR-1908-5p  hsa-miR-1908-5p  hsa-miR-1908-5p  hsa-miR-1908-5p  hsa-miR-1908-5p  hsa-miR-1908-5p  hsa-miR-1908-5p  hsa-miR-1908-5p  hsa-miR-1908-5p  hsa-miR-10400-5p  hsa-miR-10400-5p  hsa-miR-10400-5p  hsa-miR-10b-5p  hsa-miR-10b-5p  hsa-miR-10b-5p  hsa-miR-1197  hsa-miR-1197  hsa-miR-1200  hsa-miR-1200  hsa-miR-3972  hsa-miR-3972  hsa-miR-4763-3p  hsa-miR-4763-3p  hsa-miR-4763-3p  hsa-miR-4763-3p  hsa-miR-4763-3p  hsa-miR-4763-3p  hsa-miR-4763-3p  hsa-miR-4763-3p  hsa-miR-4763-3p  hsa-miR-4763-3p  hsa-miR-4763-3p  hsa-miR-4763-3p  hsa-miR-1208  hsa-miR-627-5p  hsa-miR-627-5p  hsa-miR-627-5p  hsa-miR-1226-3p  hsa-miR-1226-3p  hsa-miR-1226-3p  hsa-miR-1226-3p  hsa-miR-1226-3p  hsa-miR-1226-3p  hsa-miR-1226-3p  hsa-miR-21-3p  hsa-miR-21-3p  hsa-miR-21-3p  hsa-miR-21-3p  hsa-miR-1237-3p  hsa-miR-1237-3p  hsa-miR-1258  hsa-miR-1258  hsa-miR-1258  hsa-miR-1258  hsa-miR-125b-1-3p  hsa-miR-125b-1-3p  hsa-miR-125b-1-3p  hsa-miR-224-5p  hsa-miR-4319  hsa-miR-4319  hsa-miR-4319  hsa-miR-4319  hsa-miR-4319  hsa-miR-4319  hsa-miR-4319  hsa-miR-127-3p  hsa-miR-127-5p  hsa-miR-1275  hsa-miR-1275  hsa-miR-1275  hsa-miR-1275  hsa-miR-1275  hsa-miR-1275  hsa-miR-1275  hsa-miR-1278  hsa-miR-1278  hsa-miR-1278  hsa-miR-128-3p  hsa-miR-128-3p  hsa-miR-128-3p  hsa-miR-128-3p  hsa-miR-128-3p  hsa-miR-128-3p  hsa-miR-128-3p  hsa-miR-128-3p  hsa-miR-1290  hsa-miR-1290  hsa-miR-1290  hsa-miR-1290  hsa-miR-1296-5p  hsa-miR-1299  hsa-miR-1299  hsa-miR-1299  hsa-miR-1299  hsa-miR-1299  hsa-miR-1299  hsa-miR-1303  hsa-miR-1303  hsa-miR-1303  hsa-miR-1304-3p  hsa-miR-1304-3p  hsa-miR-1304-3p  hsa-miR-1306-5p  hsa-miR-1306-5p  hsa-miR-1306-5p  hsa-miR-1306-5p  hsa-miR-130b-3p  hsa-miR-130b-3p  hsa-miR-130b-3p  hsa-miR-130b-3p  hsa-miR-130b-3p  hsa-miR-130b-3p  hsa-miR-132-3p  hsa-miR-132-3p  hsa-miR-136-3p  hsa-miR-137-3p  hsa-miR-137-3p  hsa-miR-137-3p  hsa-miR-137-3p  hsa-miR-139-3p  hsa-miR-139-3p  hsa-miR-140-3p  hsa-miR-140-3p  hsa-miR-140-3p  hsa-miR-140-3p  hsa-miR-140-3p  hsa-miR-140-3p  hsa-miR-140-3p  hsa-miR-140-5p  hsa-miR-140-5p  hsa-miR-140-5p  hsa-miR-140-5p  hsa-miR-140-5p  hsa-miR-141-3p  hsa-miR-141-3p  hsa-miR-141-3p  hsa-miR-143-3p  hsa-miR-143-3p  hsa-miR-143-3p  hsa-miR-143-3p  hsa-miR-143-3p  hsa-miR-143-3p  hsa-miR-143-3p  hsa-miR-143-5p  hsa-miR-143-5p  hsa-miR-143-5p  hsa-miR-145-3p  hsa-miR-145-5p  hsa-miR-145-5p  hsa-miR-146a-5p  hsa-miR-146a-5p  hsa-miR-146a-5p  hsa-miR-146a-5p  hsa-miR-146a-5p  hsa-miR-148a-3p  hsa-miR-148a-3p  hsa-miR-148a-3p  hsa-miR-148a-3p  hsa-miR-151a-3p  hsa-miR-151a-5p  hsa-miR-487a-3p  hsa-miR-487a-3p  hsa-miR-487a-3p  hsa-miR-154-5p  hsa-miR-154-5p  hsa-miR-154-5p  hsa-miR-155-5p  hsa-miR-155-5p  hsa-miR-155-5p  hsa-miR-155-5p  hsa-miR-1587  hsa-miR-1587  hsa-miR-1587  hsa-miR-1587  hsa-miR-1587  hsa-miR-1587  hsa-miR-1587  hsa-miR-1587  hsa-miR-1587  hsa-miR-15b-3p  hsa-miR-15b-3p  hsa-miR-15b-3p  hsa-miR-424-5p  hsa-miR-424-5p  hsa-miR-424-5p  hsa-miR-424-5p  hsa-miR-424-5p  hsa-miR-424-5p  hsa-miR-424-5p  hsa-miR-424-5p  hsa-miR-20a-5p  hsa-miR-20a-5p  hsa-miR-20a-5p  hsa-miR-20a-5p  hsa-miR-20a-5p  hsa-miR-20a-5p  hsa-miR-20a-5p  hsa-miR-181a-2-3p  hsa-miR-181a-2-3p  hsa-miR-181a-2-3p  hsa-miR-181a-2-3p  hsa-miR-181a-2-3p  hsa-miR-181a-2-3p  hsa-miR-181b-3p  hsa-miR-181b-3p  hsa-miR-183-5p  hsa-miR-183-5p  hsa-miR-183-5p  hsa-miR-183-5p  hsa-miR-183-5p  hsa-miR-183-5p  hsa-miR-185-5p  hsa-miR-185-5p  hsa-miR-185-5p  hsa-miR-185-5p  hsa-miR-185-5p  hsa-miR-185-5p  hsa-miR-185-5p  hsa-miR-185-5p  hsa-miR-185-5p  hsa-miR-186-5p  hsa-miR-6769b-5p  hsa-miR-6769b-5p  hsa-miR-6769b-5p  hsa-miR-6769b-5p  hsa-miR-6769b-5p  hsa-miR-6769b-5p  hsa-miR-6769b-5p  hsa-miR-18a-3p  hsa-miR-18a-3p  hsa-miR-18a-3p  hsa-miR-4632-3p  hsa-miR-4632-3p  hsa-miR-4632-3p  hsa-miR-191-5p  hsa-miR-191-5p  hsa-miR-1913  hsa-miR-1913  hsa-miR-6764-5p  hsa-miR-6764-5p  hsa-miR-6764-5p  hsa-miR-6764-5p  hsa-miR-6764-5p  hsa-miR-192-5p  hsa-miR-193b-3p  hsa-miR-193b-3p  hsa-miR-193b-3p  hsa-miR-193b-3p  hsa-miR-193a-5p  hsa-miR-193a-5p  hsa-miR-196a-5p  hsa-miR-196a-5p  hsa-miR-199b-3p  hsa-miR-199b-5p  hsa-miR-199b-5p  hsa-miR-199b-5p  hsa-miR-199b-5p  hsa-miR-19b-2-5p  hsa-miR-19b-2-5p  hsa-miR-19a-3p  hsa-miR-19a-3p  hsa-miR-19a-3p  hsa-miR-19a-3p  hsa-miR-19a-3p  hsa-miR-19a-3p  hsa-miR-19a-3p  hsa-miR-19a-3p  hsa-miR-200b-5p  hsa-miR-205-5p  hsa-miR-20b-3p  hsa-miR-20b-3p  hsa-miR-21-5p  hsa-miR-21-5p  hsa-miR-21-5p  hsa-miR-21-5p  hsa-miR-21-5p  hsa-miR-214-3p  hsa-miR-214-3p  hsa-miR-214-3p  hsa-miR-214-3p  hsa-miR-214-3p  hsa-miR-214-3p  hsa-miR-214-3p  hsa-miR-214-3p  hsa-miR-218-5p  hsa-miR-218-5p  hsa-miR-218-5p  hsa-miR-218-5p  hsa-miR-22-3p  hsa-miR-22-3p  hsa-miR-22-3p  hsa-miR-22-3p  hsa-miR-22-3p  hsa-miR-22-3p  hsa-miR-22-3p  hsa-miR-22-3p  hsa-miR-221-3p  hsa-miR-221-3p  hsa-miR-221-3p  hsa-miR-221-3p  hsa-miR-221-5p  hsa-miR-221-5p  hsa-miR-221-5p  hsa-miR-221-5p  hsa-miR-23b-3p  hsa-miR-23b-3p  hsa-miR-23b-3p  hsa-miR-23b-3p  hsa-miR-23b-3p  hsa-miR-24-3p  hsa-miR-24-3p  hsa-miR-24-3p  hsa-miR-24-3p  hsa-miR-24-3p  hsa-miR-24-3p  hsa-miR-24-3p  hsa-miR-6781-3p  hsa-miR-6781-3p  hsa-miR-6781-3p  hsa-miR-26b-5p  hsa-miR-26b-5p  hsa-miR-27b-3p  hsa-miR-27b-3p  hsa-miR-27b-3p  hsa-miR-27b-3p  hsa-miR-27a-5p  hsa-miR-27a-5p  hsa-miR-27a-5p  hsa-miR-27a-5p  hsa-miR-27a-5p  hsa-miR-27a-5p  hsa-miR-27a-5p  hsa-miR-28-3p  hsa-miR-28-3p  hsa-miR-2861  hsa-miR-2861  hsa-miR-2861  hsa-miR-298  hsa-miR-299-3p  hsa-miR-299-3p  hsa-miR-299-3p  hsa-miR-299-3p  hsa-miR-299-3p  hsa-miR-299-3p  hsa-miR-29a-3p  hsa-miR-29a-3p  hsa-miR-29a-3p  hsa-miR-29a-3p  hsa-miR-29c-5p  hsa-miR-6504-5p  hsa-miR-6504-5p  hsa-miR-6504-5p  hsa-miR-6504-5p  hsa-miR-6504-5p  hsa-miR-6504-5p  hsa-miR-6504-5p  hsa-miR-6504-5p  hsa-miR-6504-5p  hsa-miR-6504-5p  hsa-miR-4727-5p  hsa-miR-4727-5p  hsa-miR-4727-5p  hsa-miR-4727-5p  hsa-miR-6071  hsa-miR-30e-3p  hsa-miR-30e-3p  hsa-miR-30e-3p  hsa-miR-30e-3p  hsa-miR-30e-3p  hsa-miR-30c-2-3p  hsa-miR-30c-2-3p  hsa-miR-30c-2-3p  hsa-miR-30c-2-3p  hsa-miR-30c-2-3p  hsa-miR-30a-5p  hsa-miR-30a-5p  hsa-miR-31-3p  hsa-miR-31-5p  hsa-miR-31-5p  hsa-miR-31-5p  hsa-miR-31-5p  hsa-miR-31-5p  hsa-miR-31-5p  hsa-miR-31-5p  hsa-miR-3117-3p  hsa-miR-3117-3p  hsa-miR-611  hsa-miR-611  hsa-miR-611  hsa-miR-611  hsa-miR-611  hsa-miR-3135a  hsa-miR-3135a  hsa-miR-3144-3p  hsa-miR-3144-3p  hsa-miR-3181  hsa-miR-3181  hsa-miR-3182  hsa-miR-3190-5p  hsa-miR-3190-5p  hsa-miR-3190-5p  hsa-miR-3190-5p  hsa-miR-3190-5p  hsa-miR-3193  hsa-miR-3193  hsa-miR-3193  hsa-miR-320a-3p  hsa-miR-323a-3p  hsa-miR-323a-3p  hsa-miR-323a-3p  hsa-miR-323b-3p  hsa-miR-324-5p  hsa-miR-324-5p  hsa-miR-324-5p  hsa-miR-324-5p  hsa-miR-324-5p  hsa-miR-324-5p  hsa-miR-324-5p  hsa-miR-329-3p  hsa-miR-329-3p  hsa-miR-329-3p  hsa-miR-329-3p  hsa-miR-335-5p  hsa-miR-337-3p  hsa-miR-337-3p  hsa-miR-337-5p  hsa-miR-337-5p  hsa-miR-339-5p  hsa-miR-339-5p  hsa-miR-339-5p  hsa-miR-339-5p  hsa-miR-345-5p  hsa-miR-4430  hsa-miR-4430  hsa-miR-4430  hsa-miR-4430  hsa-miR-4430  hsa-miR-4430  hsa-miR-4430  hsa-miR-34a-5p  hsa-miR-34a-5p  hsa-miR-34a-5p  hsa-miR-34a-5p  hsa-miR-34a-5p  hsa-miR-34a-5p  hsa-miR-34a-5p  hsa-miR-34a-5p  hsa-miR-34a-5p  hsa-miR-34a-5p  hsa-miR-34a-5p  hsa-miR-34a-5p  hsa-miR-3529-3p  hsa-miR-3529-3p  hsa-miR-3529-3p  hsa-miR-3529-3p  hsa-miR-3529-3p  hsa-miR-765  hsa-miR-765  hsa-miR-765  hsa-miR-765  hsa-miR-765  hsa-miR-765  hsa-miR-765  hsa-miR-361-3p  hsa-miR-361-3p  hsa-miR-361-3p  hsa-miR-361-3p  hsa-miR-361-3p  hsa-miR-361-3p  hsa-miR-361-3p  hsa-miR-361-5p  hsa-miR-361-5p  hsa-miR-3621  hsa-miR-3621  hsa-miR-3621  hsa-miR-3621  hsa-miR-365b-5p  hsa-miR-365b-5p  hsa-miR-365b-5p  hsa-miR-365b-5p  hsa-miR-365a-3p  hsa-miR-365a-3p  hsa-miR-365a-3p  hsa-miR-365a-3p  hsa-miR-365a-3p  hsa-miR-6864-3p  hsa-miR-6864-3p  hsa-miR-3680-5p  hsa-miR-369-5p  hsa-miR-374a-3p  hsa-miR-374a-3p  hsa-miR-374b-3p  hsa-miR-374b-3p  hsa-miR-374b-5p  hsa-miR-376a-3p  hsa-miR-376a-3p  hsa-miR-376a-3p  hsa-miR-376a-3p  hsa-miR-376a-3p  hsa-miR-376a-3p  hsa-miR-376b-5p  hsa-miR-376b-5p  hsa-miR-376c-3p  hsa-miR-376c-3p  hsa-miR-376c-3p  hsa-miR-376c-3p  hsa-miR-377-3p  hsa-miR-379-5p  hsa-miR-381-3p  hsa-miR-382-5p  hsa-miR-3919  hsa-miR-3919  hsa-miR-3919  hsa-miR-3919  hsa-miR-6806-3p  hsa-miR-6806-3p  hsa-miR-3944-5p  hsa-miR-3944-5p  hsa-miR-3944-5p  hsa-miR-3944-5p  hsa-miR-3978  hsa-miR-411-3p  hsa-miR-411-3p  hsa-miR-411-3p  hsa-miR-411-3p  hsa-miR-411-5p  hsa-miR-421  hsa-miR-421  hsa-miR-421  hsa-miR-423-3p  hsa-miR-3184-5p  hsa-miR-3184-5p  hsa-miR-3184-5p  hsa-miR-3184-5p  hsa-miR-3184-5p  hsa-miR-3184-5p  hsa-miR-424-3p  hsa-miR-424-3p  hsa-miR-425-5p  hsa-miR-4269  hsa-miR-4269  hsa-miR-4279  hsa-miR-4279  hsa-miR-4279  hsa-miR-4279  hsa-miR-4281  hsa-miR-4303  hsa-miR-4303  hsa-miR-4303  hsa-miR-4303  hsa-miR-431-5p  hsa-miR-431-5p  hsa-miR-431-5p  hsa-miR-4310  hsa-miR-4310  hsa-miR-4310  hsa-miR-4310  hsa-miR-433-5p  hsa-miR-4435  hsa-miR-4435  hsa-miR-4435  hsa-miR-4435  hsa-miR-4435  hsa-miR-4435  hsa-miR-4435  hsa-miR-4435  hsa-miR-4446-3p  hsa-miR-4446-3p  hsa-miR-4446-3p  hsa-miR-4446-3p  hsa-miR-4446-3p  hsa-miR-4446-3p  hsa-miR-4446-3p  hsa-miR-4446-3p  hsa-miR-4446-3p  hsa-miR-4446-3p  hsa-miR-4446-3p  hsa-miR-4446-3p  hsa-miR-4448  hsa-miR-4448  hsa-miR-4448  hsa-miR-4448  hsa-miR-4449  hsa-miR-4449  hsa-miR-4487  hsa-miR-4487  hsa-miR-4493  hsa-miR-4493  hsa-miR-4493  hsa-miR-4493  hsa-miR-4493  hsa-miR-4493  hsa-miR-4493  hsa-miR-449c-3p  hsa-miR-450b-5p  hsa-miR-450b-5p  hsa-miR-4515  hsa-miR-4515  hsa-miR-4515  hsa-miR-452-5p  hsa-miR-452-5p  hsa-miR-452-5p  hsa-miR-452-5p  hsa-miR-4530  hsa-miR-4530  hsa-miR-4530  hsa-miR-4530  hsa-miR-4530  hsa-miR-4530  hsa-miR-4530  hsa-miR-4531  hsa-miR-4531  hsa-miR-4531  hsa-miR-4534  hsa-miR-4534  hsa-miR-4534 | 0.0000  0.0000  0.0000  0.0000  0.0000  0.0000  0.0000  0.0061  0.0061  0.0061  0.0000  0.0000  0.0000  0.0000  0.0000  0.0000  0.0000  0.0000  0.0000  0.0000  0.0000  0.0000  0.0000  0.0000  0.0000  0.0000  0.0000  0.0000  0.0000  0.0000  0.0000  0.0000  0.0000  0.0000  0.0000  0.0000  0.0000  0.0000  0.0000  0.0002  0.0002  0.0000  0.0000  0.0088  0.0088  0.0088  0.0088  0.0088  0.0088  0.0088  0.0088  0.0088  0.0088  0.0088  0.0088  0.0000  0.0144  0.0144  0.0144  0.0293  0.0293  0.0293  0.0293  0.0293  0.0293  0.0293  0.0000  0.0000  0.0000  0.0000  0.0000  0.0000  0.0000  0.0000  0.0000  0.0000  0.0017  0.0017  0.0017  0.0000  0.0000  0.0000  0.0000  0.0000  0.0000  0.0000  0.0000  0.0000  0.0000  0.0000  0.0000  0.0000  0.0000  0.0000  0.0000  0.0000  0.0142  0.0142  0.0142  0.0000  0.0000  0.0000  0.0000  0.0000  0.0000  0.0000  0.0000  0.0000  0.0000  0.0000  0.0000  0.0368  0.0142  0.0142  0.0142  0.0142  0.0142  0.0142  0.0033  0.0033  0.0033  0.0000  0.0000  0.0000  0.0000  0.0000  0.0000  0.0000  0.0000  0.0000  0.0000  0.0000  0.0000  0.0000  0.0001  0.0001  0.0000  0.0000  0.0000  0.0000  0.0000  0.0000  0.0000  0.0000  0.0000  0.0000  0.0000  0.0000  0.0000  0.0000  0.0400  0.0400  0.0400  0.0400  0.0400  0.0487  0.0487  0.0487  0.0000  0.0000  0.0000  0.0000  0.0000  0.0000  0.0000  0.0000  0.0000  0.0000  0.0002  0.0000  0.0000  0.0000  0.0000  0.0000  0.0000  0.0000  0.0000  0.0000  0.0000  0.0000  0.0000  0.0028  0.0003  0.0003  0.0003  0.0000  0.0000  0.0000  0.0000  0.0000  0.0000  0.0000  0.0155  0.0155  0.0155  0.0155  0.0155  0.0155  0.0155  0.0155  0.0155  0.0009  0.0009  0.0009  0.0000  0.0000  0.0000  0.0000  0.0000  0.0000  0.0000  0.0000  0.0000  0.0000  0.0000  0.0000  0.0000  0.0000  0.0000  0.0000  0.0000  0.0000  0.0000  0.0000  0.0000  0.0005  0.0005  0.0000  0.0000  0.0000  0.0000  0.0000  0.0000  0.0000  0.0000  0.0000  0.0000  0.0000  0.0000  0.0000  0.0000  0.0000  0.0000  0.0000  0.0000  0.0000  0.0000  0.0000  0.0000  0.0000  0.0045  0.0045  0.0045  0.0001  0.0001  0.0001  0.0000  0.0000  0.0000  0.0000  0.0002  0.0002  0.0002  0.0002  0.0002  0.0000  0.0000  0.0000  0.0000  0.0000  0.0000  0.0000  0.0000  0.0000  0.0000  0.0000  0.0000  0.0000  0.0000  0.0000  0.0000  0.0000  0.0000  0.0000  0.0000  0.0000  0.0000  0.0000  0.0000  0.0212  0.0056  0.0330  0.0330  0.0000  0.0000  0.0000  0.0000  0.0000  0.0000  0.0000  0.0000  0.0000  0.0000  0.0000  0.0000  0.0000  0.0000  0.0000  0.0000  0.0000  0.0000  0.0000  0.0000  0.0000  0.0000  0.0000  0.0000  0.0000  0.0000  0.0000  0.0000  0.0000  0.0000  0.0000  0.0000  0.0000  0.0000  0.0000  0.0000  0.0000  0.0000  0.0000  0.0000  0.0000  0.0000  0.0000  0.0000  0.0000  0.0000  0.0000  0.0000  0.0000  0.0000  0.0000  0.0000  0.0000  0.0000  0.0005  0.0005  0.0005  0.0005  0.0005  0.0005  0.0005  0.0000  0.0000  0.0069  0.0069  0.0069  0.0001  0.0007  0.0007  0.0007  0.0007  0.0007  0.0007  0.0000  0.0000  0.0000  0.0000  0.0002  0.0000  0.0000  0.0000  0.0000  0.0000  0.0000  0.0000  0.0000  0.0000  0.0000  0.0010  0.0010  0.0010  0.0010  0.0033  0.0000  0.0000  0.0000  0.0000  0.0000  0.0039  0.0039  0.0039  0.0039  0.0039  0.0000  0.0000  0.0139  0.0000  0.0000  0.0000  0.0000  0.0000  0.0000  0.0000  0.0041  0.0041  0.0001  0.0001  0.0001  0.0001  0.0001  0.0000  0.0000  0.0022  0.0022  0.0460  0.0460  0.0000  0.0000  0.0000  0.0000  0.0000  0.0000  0.0118  0.0118  0.0118  0.0000  0.0000  0.0000  0.0000  0.0018  0.0387  0.0387  0.0387  0.0387  0.0387  0.0387  0.0387  0.0004  0.0004  0.0004  0.0004  0.0000  0.0071  0.0071  0.0000  0.0000  0.0000  0.0000  0.0000  0.0000  0.0000  0.0036  0.0036  0.0036  0.0036  0.0036  0.0036  0.0036  0.0000  0.0000  0.0000  0.0000  0.0000  0.0000  0.0000  0.0000  0.0000  0.0000  0.0000  0.0000  0.0001  0.0001  0.0001  0.0001  0.0001  0.0000  0.0000  0.0000  0.0000  0.0000  0.0000  0.0000  0.0000  0.0000  0.0000  0.0000  0.0000  0.0000  0.0000  0.0000  0.0000  0.0000  0.0000  0.0000  0.0000  0.0083  0.0083  0.0083  0.0083  0.0000  0.0000  0.0000  0.0000  0.0000  0.0000  0.0000  0.0000  0.0000  0.0095  0.0095  0.0037  0.0037  0.0006  0.0000  0.0000  0.0000  0.0000  0.0000  0.0000  0.0029  0.0029  0.0000  0.0000  0.0000  0.0000  0.0109  0.0000  0.0000  0.0000  0.0309  0.0309  0.0309  0.0309  0.0003  0.0003  0.0000  0.0000  0.0000  0.0000  0.0055  0.0000  0.0000  0.0000  0.0000  0.0000  0.0009  0.0009  0.0009  0.0000  0.0132  0.0132  0.0132  0.0132  0.0132  0.0132  0.0000  0.0000  0.0000  0.0456  0.0456  0.0000  0.0000  0.0000  0.0000  0.0005  0.0000  0.0000  0.0000  0.0000  0.0003  0.0003  0.0003  0.0305  0.0305  0.0305  0.0305  0.0000  0.0000  0.0000  0.0000  0.0000  0.0000  0.0000  0.0000  0.0000  0.0356  0.0356  0.0356  0.0356  0.0356  0.0356  0.0356  0.0356  0.0356  0.0356  0.0356  0.0356  0.0000  0.0000  0.0000  0.0000  0.0000  0.0000  0.0446  0.0446  0.0002  0.0002  0.0002  0.0002  0.0002  0.0002  0.0002  0.0238  0.0085  0.0085  0.0001  0.0001  0.0001  0.0000  0.0000  0.0000  0.0000  0.0142  0.0142  0.0142  0.0142  0.0142  0.0142  0.0142  0.0190  0.0190  0.0190  0.0004  0.0004  0.0004 | RHOA  CASP3  CASP5  MAPK11  PRKAR2A  TLR4  TNFRSF1B  PRKACB  PRKAR1A  TLR4  NLRP2  CASP3  IL1R1  MAPK1  NAIP  NEK7  PTGER4  CASP3  NOL3  RHOA  TNF  TRAF3  GBP2  GBP5  MAPK13  NGFR  NLRP1  NLRP8  NOL3  PRKAR1B  TLR3  NLRP1  PRKAR2A  TNFRSF1A  CASP8  PRKACA  TNFRSF11B  CASP5  PRKAG2  NLRP3  TNFRSF1B  IL1B  TNF  BAX  CASP9  FOXO3  MAPK13  NGFR  PRKACA  PRKAG1  PRKAR1B  TLR6  TNF  TNFRSF1B  TRAF3  CASP3  NLRP1  NLRP10  TXNIP  CASP8  GSDME  MAPK1  MAPK12  NEK7  PRKACA  PTGER4  FOXO3  NEK7  PRKAR2A  TLR3  NLRP13  TRAF3  FOXO3  MAPK13  PRKAR1A  PTGER4  CASP9  IL1B  TNF  BAX  ELAVL1  GBP2  MAPK12  MAPK14  NLRP2  PRKAR2A  TNFRSF1B  PRKAR2B  NEK7  GBP4  NEK7  NGFR  P2RX7  PRKACA  PRKAR1B  TNFRSF1B  GBP2  NEK7  PRKACB  BAX  CASP1  CASP5  CASP8  MAPK14  NGFR  TLR8  TXNIP  CASP1  IRF2  NLRP3  PTGER4  NFKB1  CASP5  GBP2  GBP4  NAIP  NLRP9  PTGER4  MAPK14  P2RX7  PRKACB  GBP5  GBP7  PRKAR2A  MAPK13  NLRP13  TLR4  TLR6  CASP8  MAPK1  PRKACB  TLR4  TNF  TNFRSF1B  MAPK1  PRKAG2  CASP8  CASP3  GBP3  NLRP9  PRKAR2B  ELAVL1  FOXO3  CASP5  DHX9  MAPK1  PRKAR2A  RHOA  TLR1  TLR8  CASP3  NAIP  NLRP11  TLR4  TNF  IL1A  PRKACB  TXNIP  BAX  CASP5  CASP8  IL18  MAPK1  MAPK12  TLR2  CASP8  GSDME  TLR7  PRKAG1  NAIP  TNFRSF11B  IL1R1  TLR1  TLR10  TLR4  TLR9  GBP3  PRKAG2  TNFRSF1B  TXNIP  CASP5  MAPK11  PRKACB  RHOA  TLR1  GBP3  GBP5  PRKACB  FOXO3  PRKAR1A  PRKAR1B  RHOA  CASP4  CASP5  IL1R1  NGFR  NOL3  PRKACA  PRKAR2A  RHOA  TRAF3  CASP8  MAPK13  NEK7  CASP5  GSDMD  mir-9  NOL3  PANX1  PRKAR1A  PRKAR2A  TLR1  CASP8  GBP3  MAPK1  PRKACB  TLR7  TNF  TXNIP  CASP3  CASP9  MAPK12  MAPK13  NLRP10  PRKACG  GSDME  TNFRSF11B  MAPK13  NFKB1  PRKACB  PRKACG  PRKAR1A  TLR7  AIM2  CASP1  IL1B  MAPK11  MAPK13  PRKAR2A  PRKAR2B  RHOA  TNF  NEK7  CASP5  GSDMD  NFKB2  PRKACA  PRKAR2A  PYCARD  RHOA  GSDMD  PRKAR1A  TLR3  MAPK13  NAIP  TLR6  TLR3  TRAF3  TLR5  TNFRSF1B  MAPK13  MEFV  NOL3  TNF  TNFRSF1B  PRKAR1A  CASP9  GSDMD  NGFR  TNFRSF1B  GBP5  NLRP6  MAPK1  TLR5  NLRP1  NFKB1  NLRP7  PRKAG2  TRAF3  IL18  P2RX7  CASP8  IL1R1  MAPK1  PRKACB  PRKAR2A  TLR5  TNF  TNFRSF1B  HMGB1  MEFV  IRF2  MEFV  CASP8  IL1B  NEK7  TNF  TNFRSF11B  BAX  CASP1  IL1R1  MAPK1  PRKAR1A  PRKAR2A  TLR5  TRAF3  NLRP9  PRKACG  PRKAG1  PRKAR2B  GSDMD  MAPK1  MAPK14  NLRP14  NLRP3  PRKAR2A  TLR8  TNFRSF1B  CASP3  FOXO3  IRF2  TNFRSF11B  GSDMD  IL1A  IL1B  PRKAR2A  CASP3  IL18  IRF2  NEK7  PTGER4  BAX  CASP9  IL1A  MAPK14  PTGER4  TNF  TNFRSF1A  IL18  MAPK11  P2RX7  GBP3  TRAF3  BAX  CASP8  NGFR  NLRP10  CASP5  CASP8  IL1R1  MAPK14  PRKACA  PRKACG  RHOA  NAIP  TRAF3  MAPK13  PRKACA  TXNIP  TLR3  CASP1  CASP5  IL1B  MEFV  PRKAR2A  TNFRSF1B  CASP3  CASP8  FOXO3  TNFRSF1A  NEK7  CASP5  IL1B  IL1R1  MEFV  NLRP1  P2RX7  PRKACA  PRKACG  TLR6  TXNIP  IL1B  MAPK13  RHOA  TRAF3  MAPK13  GBP1  GSDME  HMGB1  IL1B  MAPK13  CASP1  CASP8  MAPK13  MAPK14  PRKACA  CASP3  IL1A  RHOA  ELAVL1  GBP3  GSDME  IL1R1  MAPK11  PRKAR2A  TLR4  IL1A  TLR8  CASP8  MAPK11  TLR6  TNFRSF1B  TRAF3  GBP2  PRKAR2A  CASP1  PTGER4  GBP2  PRKAR1B  PRKACB  GSDMD  IL1R1  TLR10  TNF  TNFRSF1B  CASP3  CASP9  NLRP1  PRKAG2  CASP3  GBP3  PRKAR1A  RHOA  CASP1  CASP8  ELAVL1  MAPK13  NGFR  NLRP11  NOL3  DHX9  NLRP9  PRKACB  TNFRSF1B  GBP7  CASP3  NLRP10  MAPK13  TLR4  CASP8  NLRP4  NLRP7  PRKACA  CASP5  GBP4  IL1R1  NGFR  PRKACA  PRKACG  PRKAR1A  TNFRSF1B  BAX  GBP2  GBP5  GSDMB  IL1R1  MAPK13  NAIP  NGFR  PRKACB  PRKAG1  TLR4  TNFRSF11B  CASP3  HMGB1  PRKACB  PRKAR2A  PTGER4  BAX  CASP9  IL1R1  NLRP2  PRKACG  PRKAR2A  TNFRSF11B  BAX  MAPK13  PRKACG  PRKAG1  PRKAR1B  TNF  TRAF3  PRKACB  PRKAR2B  MEFV  NLRP1  P2RX7  PRKAR1B  NLRP12  NLRP6  PRKAR2A  TLR2  BAX  CASP5  GBP5  IL1A  PRKAR2A  NLRP11  PRKACB  NLRP10  PRKACB  NEK7  PRKAR2B  NAIP  TRAF3  NLRP11  CASP3  CASP8  CASP9  PRKACB  TLR2  TLR5  NAIP  NLRP10  GSDME  PRKAR2B  TLR3  TLR5  NLRP11  GSDMD  PRKAR2A  CASP3  IL1A  NFKB1  NLRP14  PRKACB  CASP3  CASP8  CASP8  GSDME  PRKAR2A  TRAF3  GBP2  GSDME  IL1A  NLRP2  NLRP4  TXNIP  CASP3  MAPK14  TLR1  PRKAG2  CASP9  MAPK13  NGFR  NLRP4  PRKACA  PRKAR1B  GSDME  TRAF3  GBP5  NAIP  PRKAR2A  CASP5  MAPK13  PRKAG1  PRKAG2  MAPK11  IL1B  MAPK1  PRKAR2A  PTGER4  IL1R1  MEFV  PRKAR2B  MAPK14  PRKACB  PRKAG2  TNF  GBP2  GSDME  NEK7  NLRP12  NLRP8  TLR10  TLR5  TLR7  TNF  CASP4  CASP5  GSDMD  IL18  IL1B  MAPK13  MEFV  NFKB1  NGFR  PRKACA  PRKACG  RHOA  CASP4  FOXO3  MEFV  NLRP10  MAPK11  MAPK14  BAX  GBP2  IRF2  MAPK13  NLRP4  PRKAR2A  TLR5  TNF  TNFRSF1A  NLRP9  HMGB1  NFKB1  CASP9  MAPK1  TLR6  GSDME  PRKAR2B  RHOA  TLR1  CASP8  GBP5  IRF2  NFKB1  NOL3  PRKAG1  TLR4  NLRP2  PRKAR1A  PRKAR2A  IL18  MAPK13  NLRP1 | High (predicted)  Experimentally Observed,High (predicted)  Moderate (predicted)  Moderate (predicted)  High (predicted)  Experimentally Observed  Moderate (predicted)  High (predicted)  Moderate (predicted)  Moderate (predicted)  High (predicted)  Moderate (predicted)  Moderate (predicted)  Moderate (predicted)  Moderate (predicted)  Moderate (predicted)  Moderate (predicted)  Moderate (predicted)  Moderate (predicted)  Moderate (predicted)  Moderate (predicted)  Moderate (predicted)  Moderate (predicted)  High (predicted)  Moderate (predicted)  Moderate (predicted)  Moderate (predicted)  Moderate (predicted)  Moderate (predicted)  Moderate (predicted)  Moderate (predicted)  Moderate (predicted)  Moderate (predicted)  High (predicted)  Moderate (predicted)  Moderate (predicted)  Moderate (predicted)  Moderate (predicted)  Moderate (predicted)  Moderate (predicted)  Moderate (predicted)  Moderate (predicted)  High (predicted)  Moderate (predicted)  Moderate (predicted)  High (predicted)  Moderate (predicted)  High (predicted)  Moderate (predicted)  Moderate (predicted)  High (predicted)  Moderate (predicted)  Moderate (predicted)  High (predicted)  Moderate (predicted)  Moderate (predicted)  Moderate (predicted)  High (predicted)  Moderate (predicted)  Moderate (predicted)  Moderate (predicted)  Moderate (predicted)  Moderate (predicted)  Moderate (predicted)  Moderate (predicted)  High (predicted)  Moderate (predicted)  High (predicted)  Moderate (predicted)  Moderate (predicted)  Moderate (predicted)  Moderate (predicted)  Moderate (predicted)  Moderate (predicted)  Moderate (predicted)  Moderate (predicted)  Moderate (predicted)  Experimentally Observed  Experimentally Observed  Experimentally Observed  Experimentally Observed  Moderate (predicted)  High (predicted)  Moderate (predicted)  Moderate (predicted)  Moderate (predicted)  Moderate (predicted)  Moderate (predicted)  Moderate (predicted)  Moderate (predicted)  Moderate (predicted)  Moderate (predicted)  High (predicted)  High (predicted)  Moderate (predicted)  Moderate (predicted)  Moderate (predicted)  High (predicted)  Moderate (predicted)  High (predicted)  Moderate (predicted)  Moderate (predicted)  High (predicted)  Moderate (predicted)  Moderate (predicted)  Moderate (predicted)  Experimentally Observed  Moderate (predicted)  Moderate (predicted)  Moderate (predicted)  Moderate (predicted)  Moderate (predicted)  High (predicted)  Moderate (predicted)  Moderate (predicted)  Moderate (predicted)  Moderate (predicted)  Moderate (predicted)  Moderate (predicted)  Moderate (predicted)  Moderate (predicted)  Moderate (predicted)  High (predicted)  Moderate (predicted)  Moderate (predicted)  Moderate (predicted)  Moderate (predicted)  High (predicted)  Moderate (predicted)  Moderate (predicted)  Moderate (predicted)  Moderate (predicted)  Moderate (predicted)  Moderate (predicted)  Moderate (predicted)  Moderate (predicted)  Moderate (predicted)  High (predicted)  High (predicted)  Moderate (predicted)  Moderate (predicted)  Experimentally Observed  Moderate (predicted)  Moderate (predicted)  Moderate (predicted)  Moderate (predicted)  Moderate (predicted)  Moderate (predicted)  Moderate (predicted)  Moderate (predicted)  High (predicted)  Moderate (predicted)  Moderate (predicted)  Moderate (predicted)  High (predicted)  Moderate (predicted)  High (predicted)  Moderate (predicted)  Moderate (predicted)  Moderate (predicted)  Moderate (predicted)  High (predicted)  Moderate (predicted)  Experimentally Observed  Moderate (predicted)  Moderate (predicted)  Moderate (predicted)  Moderate (predicted)  Moderate (predicted)  Moderate (predicted)  Moderate (predicted)  Experimentally Observed  Experimentally Observed  Experimentally Observed  Experimentally Observed  Experimentally Observed  Moderate (predicted)  Moderate (predicted)  Moderate (predicted)  Moderate (predicted)  High (predicted)  High (predicted)  Moderate (predicted)  Moderate (predicted)  Moderate (predicted)  Moderate (predicted)  Moderate (predicted)  Moderate (predicted)  Moderate (predicted)  Moderate (predicted)  Moderate (predicted)  Experimentally Observed  Moderate (predicted)  Moderate (predicted)  High (predicted)  Moderate (predicted)  Moderate (predicted)  High (predicted)  Moderate (predicted)  Moderate (predicted)  Moderate (predicted)  Moderate (predicted)  Moderate (predicted)  Moderate (predicted)  High (predicted)  Moderate (predicted)  Experimentally Observed  Moderate (predicted)  Experimentally Observed  Moderate (predicted)  Moderate (predicted)  Moderate (predicted)  Moderate (predicted)  Moderate (predicted)  Moderate (predicted)  Moderate (predicted)  Experimentally Observed  Experimentally Observed  Moderate (predicted)  Moderate (predicted)  Moderate (predicted)  Moderate (predicted)  Moderate (predicted)  Moderate (predicted)  Moderate (predicted)  Moderate (predicted)  Moderate (predicted)  Moderate (predicted)  Moderate (predicted)  Moderate (predicted)  Moderate (predicted)  Moderate (predicted)  Moderate (predicted)  High (predicted)  Moderate (predicted)  Moderate (predicted)  Moderate (predicted)  High (predicted)  Moderate (predicted)  Moderate (predicted)  Experimentally Observed  Moderate (predicted)  High (predicted)  Moderate (predicted)  Moderate (predicted)  Moderate (predicted)  High (predicted)  High (predicted)  Moderate (predicted)  Moderate (predicted)  Moderate (predicted)  Moderate (predicted)  Moderate (predicted)  High (predicted)  Moderate (predicted)  Moderate (predicted)  Experimentally Observed  Moderate (predicted)  Moderate (predicted)  Moderate (predicted)  Moderate (predicted)  High (predicted)  Moderate (predicted)  Moderate (predicted)  High (predicted)  Moderate (predicted)  Moderate (predicted)  Moderate (predicted)  Moderate (predicted)  Moderate (predicted)  Moderate (predicted)  Moderate (predicted)  Moderate (predicted)  Moderate (predicted)  Moderate (predicted)  Moderate (predicted)  Moderate (predicted)  Moderate (predicted)  Moderate (predicted)  Moderate (predicted)  Moderate (predicted)  Moderate (predicted)  Moderate (predicted)  Moderate (predicted)  Moderate (predicted)  Moderate (predicted)  Moderate (predicted)  Moderate (predicted)  Moderate (predicted)  Moderate (predicted)  Moderate (predicted)  Moderate (predicted)  Moderate (predicted)  Moderate (predicted)  Moderate (predicted)  Moderate (predicted)  Experimentally Observed  Moderate (predicted)  High (predicted)  Moderate (predicted)  Moderate (predicted)  Moderate (predicted)  Moderate (predicted)  Moderate (predicted)  Moderate (predicted)  Moderate (predicted)  Moderate (predicted)  Moderate (predicted)  Moderate (predicted)  Moderate (predicted)  Moderate (predicted)  Moderate (predicted)  High (predicted)  Moderate (predicted)  High (predicted)  Moderate (predicted)  High (predicted)  Moderate (predicted)  Moderate (predicted)  Experimentally Observed  High (predicted)  Moderate (predicted)  Moderate (predicted)  Moderate (predicted)  Moderate (predicted)  Moderate (predicted)  Moderate (predicted)  High (predicted)  Moderate (predicted)  Moderate (predicted)  Moderate (predicted)  Experimentally Observed  Moderate (predicted)  High (predicted)  Experimentally Observed,Moderate (predicted)  Moderate (predicted)  Moderate (predicted)  Moderate (predicted)  Moderate (predicted)  Moderate (predicted)  Moderate (predicted)  Moderate (predicted)  Moderate (predicted)  Experimentally Observed  Moderate (predicted)  Moderate (predicted)  Moderate (predicted)  Moderate (predicted)  High (predicted)  Moderate (predicted)  Moderate (predicted)  Moderate (predicted)  Moderate (predicted)  Moderate (predicted)  Moderate (predicted)  Moderate (predicted)  Moderate (predicted)  Moderate (predicted)  High (predicted)  Moderate (predicted)  Moderate (predicted)  Moderate (predicted)  Moderate (predicted)  Moderate (predicted)  Moderate (predicted)  Moderate (predicted)  Moderate (predicted)  High (predicted)  High (predicted)  High (predicted)  Moderate (predicted)  Moderate (predicted)  Moderate (predicted)  Moderate (predicted)  High (predicted)  Moderate (predicted)  High (predicted)  Moderate (predicted)  Moderate (predicted)  Moderate (predicted)  Moderate (predicted)  High (predicted)  High (predicted)  Moderate (predicted)  Moderate (predicted)  High (predicted)  Moderate (predicted)  Moderate (predicted)  Moderate (predicted)  High (predicted)  Moderate (predicted)  Moderate (predicted)  Moderate (predicted)  High (predicted)  Moderate (predicted)  Moderate (predicted)  High (predicted)  Moderate (predicted)  Moderate (predicted)  Moderate (predicted)  High (predicted)  Moderate (predicted)  Moderate (predicted)  Moderate (predicted)  High (predicted)  Moderate (predicted)  Moderate (predicted)  Moderate (predicted)  High (predicted)  Moderate (predicted)  Moderate (predicted)  Moderate (predicted)  Moderate (predicted)  Moderate (predicted)  Moderate (predicted)  Moderate (predicted)  Moderate (predicted)  Moderate (predicted)  High (predicted)  Moderate (predicted)  Moderate (predicted)  Moderate (predicted)  Moderate (predicted)  Moderate (predicted)  Moderate (predicted)  High (predicted)  Moderate (predicted)  Moderate (predicted)  Moderate (predicted)  Moderate (predicted)  Moderate (predicted)  Moderate (predicted)  Moderate (predicted)  Moderate (predicted)  Moderate (predicted)  Moderate (predicted)  Moderate (predicted)  Moderate (predicted)  High (predicted)  High (predicted)  Moderate (predicted)  Moderate (predicted)  Moderate (predicted)  Moderate (predicted)  Moderate (predicted)  Moderate (predicted)  Moderate (predicted)  Moderate (predicted)  Moderate (predicted)  Moderate (predicted)  High (predicted)  Moderate (predicted)  Moderate (predicted)  Moderate (predicted)  Moderate (predicted)  Moderate (predicted)  Moderate (predicted)  Moderate (predicted)  Moderate (predicted)  Moderate (predicted)  Moderate (predicted)  Moderate (predicted)  Moderate (predicted)  Moderate (predicted)  High (predicted)  Moderate (predicted)  High (predicted)  Moderate (predicted)  Moderate (predicted)  Moderate (predicted)  Moderate (predicted)  Moderate (predicted)  Moderate (predicted)  Moderate (predicted)  Moderate (predicted)  High (predicted)  Moderate (predicted)  Moderate (predicted)  High (predicted)  High (predicted)  Moderate (predicted)  Moderate (predicted)  Moderate (predicted)  Moderate (predicted)  High (predicted)  Moderate (predicted)  Moderate (predicted)  Moderate (predicted)  Moderate (predicted)  Moderate (predicted)  High (predicted)  High (predicted)  Moderate (predicted)  Moderate (predicted)  Moderate (predicted)  Moderate (predicted)  Moderate (predicted)  Moderate (predicted)  Moderate (predicted)  Moderate (predicted)  Moderate (predicted)  Moderate (predicted)  High (predicted)  Moderate (predicted)  Moderate (predicted)  High (predicted)  Moderate (predicted)  Moderate (predicted)  Moderate (predicted)  Moderate (predicted)  Moderate (predicted)  Moderate (predicted)  Moderate (predicted)  Moderate (predicted)  Moderate (predicted)  Moderate (predicted)  High (predicted)  High (predicted)  Moderate (predicted)  High (predicted)  Moderate (predicted)  Moderate (predicted)  Moderate (predicted)  Moderate (predicted)  Moderate (predicted)  Moderate (predicted)  Moderate (predicted)  Moderate (predicted)  Moderate (predicted)  High (predicted)  Moderate (predicted)  Moderate (predicted)  High (predicted)  Moderate (predicted)  Moderate (predicted)  Moderate (predicted)  Moderate (predicted)  High (predicted)  Moderate (predicted)  Moderate (predicted)  Moderate (predicted)  Moderate (predicted)  Moderate (predicted)  Moderate (predicted)  Moderate (predicted)  Moderate (predicted)  Moderate (predicted)  Experimentally Observed  High (predicted)  Moderate (predicted)  Moderate (predicted)  Moderate (predicted)  High (predicted)  Moderate (predicted)  High (predicted)  Moderate (predicted)  High (predicted)  High (predicted)  Moderate (predicted)  Moderate (predicted)  Moderate (predicted)  Moderate (predicted)  High (predicted)  Moderate (predicted)  Moderate (predicted)  Moderate (predicted)  Moderate (predicted)  Moderate (predicted)  Moderate (predicted)  Moderate (predicted)  Moderate (predicted)  Moderate (predicted)  Moderate (predicted)  High (predicted)  Moderate (predicted)  Moderate (predicted)  Moderate (predicted)  Moderate (predicted)  High (predicted)  Moderate (predicted)  High (predicted)  Moderate (predicted)  High (predicted)  Moderate (predicted)  Moderate (predicted)  Moderate (predicted)  Moderate (predicted)  Moderate (predicted)  Moderate (predicted)  Moderate (predicted)  Moderate (predicted)  Moderate (predicted)  Moderate (predicted)  Moderate (predicted)  Moderate (predicted)  Moderate (predicted)  Moderate (predicted)  Moderate (predicted)  Moderate (predicted)  High (predicted)  High (predicted)  Moderate (predicted)  Moderate (predicted)  Moderate (predicted)  Moderate (predicted)  Moderate (predicted)  Moderate (predicted)  Moderate (predicted)  Moderate (predicted)  Moderate (predicted)  High (predicted)  Moderate (predicted)  High (predicted)  Moderate (predicted)  High (predicted)  Moderate (predicted)  Moderate (predicted)  Moderate (predicted)  Moderate (predicted)  Moderate (predicted)  Moderate (predicted)  Moderate (predicted)  Moderate (predicted)  Moderate (predicted)  Moderate (predicted)  High (predicted)  Moderate (predicted)  Moderate (predicted)  Moderate (predicted)  High (predicted)  Moderate (predicted)  Moderate (predicted)  Moderate (predicted)  Moderate (predicted)  Moderate (predicted)  High (predicted)  Moderate (predicted)  Moderate (predicted) |

*Data were analyzed by IPA software. PRGs: pyroptosis-related genes.
