# Supplementary material for: Mesenchymal Stem Cells-Derived Exosomes Alleviate Acute Lung Injury by Inhibiting Alveolar Macrophage Pyroptosis
Source: Stem Cells Transl Med. 2024 Feb 13;13(4):371–86. doi: 10.1093/stcltm/szad094 (PMC11016849; doi:10.1093/stcltm/szad094)
Supplement: szad094_suppl_Supplementary_Materials [file szad094_suppl_supplementary_materials.zip › szad094_suppl_Supplementary_Tables_S4.docx]

**Table S4. The differential proteins between MSCs-Exo and MRC-5-Exo.**

| **Accession** | **Gene Symbol** | **Ratio** | ***P*-value** |
| --- | --- | --- | --- |
| \| P01308 \| \| --- \| \| Q9NZP8 \| \| P43251 \| \| P00915 \| \| O75460 \| \| Q7Z7G0 \| \| Q9UGM5 \| \| Q66K66 \| \| P02654 \| \| P02042 \| \| P0C0L5 \| \| Q03405 \| \| Q9UNN8 \| \| O14556 \| \| Q01105 \| \| P07333 \| \| P68871 \| \| P07359 \| \| P01859 \| \| P04217 \| \| Q9P2B2 \| \| O75351 \| \| P84085 \| \| P04066 \| \| P02760 \| \| Q9HB71 \| \| P09972 \| \| P02652 \| \| Q4V9L6 \| \| P00738 \| \| O60711 \| \| P16403 \| \| P35542 \| \| P01011 \| \| P19652 \| \| P02766 \| \| Q9Y3I0 \| \| P43652 \| \| P61081 \| \| P08571 \| \| P00450 \| \| Q6UXB8 \| \| P02750 \| \| P80108 \| \| P06276 \| \| P07814 \| \| O60831 \| \| P35080 \| \| P04180 \| \| P01009 \| \| P01834 \| \| P25311 \| \| Q92820 \| \| Q9NP79 \| \| P02790 \| \| Q96PD5 \| \| Q9H223 \| \| P22792 \| \| P40261 \| \| Q6UX71 \| \| Q9BUD6 \| \| O75882 \| \| O60687 \| \| P00739 \| \| P01857 \| \| P01871 \| \| P12259 \| \| O75954 \| \| Q9BTY2 \| \| P17174 \| \| P02763 \| \| P02655 \| \| P22303 \| \| P43007 \| \| Q99832 \| \| P01019 \| \| Q15043 \| \| P12110 \| \| Q01459 \| \| P62241 \| \| P05155 \| \| P15169 \| \| P51570 \| \| P12109 \| \| P61970 \| \| Q9Y600 \| \| P07384 \| \| P49411 \| \| P62424 \| \| Q15113 \| \| P08195 \| \| P05386 \| \| P04040 \| \| P62244 \| \| P09429 \| \| P02765 \| \| O00560 \| \| P13693 \| \| P26006 \| \| P46781 \| \| P30044 \| \| Q96FQ6 \| \| P41180 \| \| P36578 \| \| P06703 \| \| Q08380 \| \| Q96S97 \| \| P01111 \| \| P80723 \| \| Q13228 \| \| Q15404 \| \| Q96AM1 \| \| P10606 \| \| P05452 \| \| P62906 \| \| P14618 \| \| P62873 \| \| P61160 \| \| P13612 \| \| Q15582 \| \| P69905 \| \| P07225 \| \| P68371 \| \| Q16698 \| \| Q13740 \| \| Q02818 \| \| P54289 \| \| P00558 \| \| P62851 \| \| P13497 \| \| P61224 \| \| P09382 \| \| P51148 \| \| Q9NVM1 \| \| P05023 \| \| P61353 \| \| P63244 \| \| P17813 \| \| O14786 \| \| Q9H4G4 \| \| P04899 \| \| P63261 \| \| P50991 \| \| P08133 \| \| P30084 \| \| P22314 \| \| P12955 \| \| P31949 \| \| P62937 \| \| P61247 \| \| P13639 \| \| P35613 \| \| P62834 \| \| P18065 \| \| Q71U36 \| \| P30085 \| \| P13611 \| \| P60033 \| \| Q96EK6 \| \| P04114 \| \| P05090 \| \| P13987 \| \| P50281 \| \| P35858 \| \| P23396 \| \| P01023 \| \| P68104 \| \| Q14624 \| \| P62854 \| \| Q12884 \| \| Q15366 \| \| P27487 \| \| Q99623 \| \| P07355 \| \| P18124 \| \| P06733 \| \| P08238 \| \| P15880 \| \| P50995 \| \| P07339 \| \| B9A064 \| \| P46777 \| \| P05556 \| \| P08253 \| \| P25788 \| \| P21589 \| \| P21926 \| \| P59998 \| \| P08648 \| \| O95497 \| \| Q09666 \| \| P60174 \| \| P04083 \| \| P07900 \| \| P01033 \| \| Q9H4B7 \| \| P08572 \| \| P18077 \| \| Q13724 \| \| P31946 \| \| P07737 \| \| P04179 \| \| O75083 \| \| P49368 \| \| Q00839 \| \| P05546 \| \| P62258 \| \| P43121 \| \| P05388 \| \| Q9NZN4 \| \| P09619 \| \| O00159 \| \| P61978 \| \| P08567 \| \| P62736 \| \| Q04941 \| \| Q58FF6 \| \| P0DP25 \| \| P62701 \| \| P15151 \| \| P46778 \| \| P14923 \| \| P08865 \| \| Q14699 \| \| P55072 \| \| P00734 \| \| Q14764 \| \| Q8IVF7 \| \| P04792 \| \| P62987 \| \| O43854 \| \| O00299 \| \| Q02878 \| \| P20742 \| \| P27348 \| \| P19022 \| \| P78371 \| \| Q13404 \| \| P61769 \| \| Q9NZN3 \| \| P01024 \| \| O95183 \| \| P60981 \| \| Q96QD8 \| \| P20073 \| \| P23634 \| \| O00161 \| \| P17301 \| \| Q8WUM4 \| \| P02774 \| \| P01116 \| \| P35221 \| \| Q9BY43 \| \| Q9NRY6 \| \| P19338 \| \| P30050 \| \| P26038 \| \| P02771 \| \| P04075 \| \| P63000 \| \| A0A087X1C5 \| \| O15031 \| \| P06737 \| \| Q96I99 \| \| Q9H3Z4 \| \| P08670 \| \| Q9NZM1 \| \| Q13308 \| \| P54709 \| \| Q92626 \| \| O95084 \| \| P55084 \| \| P61981 \| \| P30101 \| \| Q9HCU0 \| \| Q9H8M2 \| \| Q06481 \| \| Q01995 \| \| Q93070 \| \| P00533 \| \| P60660 \| \| Q9Y2G3 \| \| Q92692 \| \| Q8WWZ4 \| \| O60701 \| \| P00505 \| \| Q9ULC3 \| \| P09486 \| \| P08758 \| \| P02452 \| \| Q07020 \| \| P98160 \| \| P02751 \| \| P02461 \| \| Q15293 \| \| P31939 \| \| P63104 \| \| Q14315 \| \| P61313 \| \| Q9Y4K0 \| \| P30038 \| \| Q5JWF2 \| \| P11142 \| \| P00338 \| \| Q96JB1 \| \| P22392 \| \| P02748 \| \| P35579 \| \| Q15019 \| \| P39060 \| \| Q04756 \| \| P52907 \| \| P17931 \| \| Q9Y262 \| \| Q9NQC3 \| \| P04424 \| \| Q8NFP9 \| \| P14625 \| \| P19827 \| \| P11166 \| \| P01008 \| \| P36955 \| \| Q06033 \| \| Q9BRL6 \| \| P62820 \| \| P07195 \| \| P18621 \| \| P11717 \| \| P68431 \| \| P39023 \| \| P07996 \| \| P06727 \| \| P51149 \| \| P62330 \| \| Q9Y678 \| \| O75340 \| \| P60866 \| \| P19823 \| \| P32119 \| \| P68036 \| \| Q9H5V8 \| \| P02788 \| \| P62491 \| \| P49755 \| \| Q99829 \| \| P12814 \| \| P08123 \| \| P53396 \| \| P00488 \| \| O14818 \| \| Q86YZ3 \| \| P15311 \| \| P62995 \| \| P16070 \| \| O15260 \| \| P62070 \| \| P35241 \| \| P20908 \| \| P62805 \| \| P50914 \| \| O14756 \| \| O43852 \| \| P22352 \| \| P00742 \| \| P10809 \| \| P60903 \| \| Q9Y490 \| \| P00367 \| \| P61019 \| \| P62917 \| \| O60716 \| \| P34897 \| \| P52565 \| \| O75531 \| \| Q5VU97 \| \| P21333 \| \| Q9Y4F1 \| \| Q12805 \| \| P00747 \| \| Q5ZPR3 \| \| P84098 \| \| Q08722 \| \| Q96BY6 \| \| Q6UVK1 \| \| P39748 \| \| P62847 \| \| Q99584 \| \| P14543 \| \| O00391 \| \| Q99798 \| \| Q08431 \| \| P09871 \| \| Q04917 \| \| P61106 \| \| Q86Y39 \| \| Q15365 \| \| P18206 \| \| P38646 \| \| P50454 \| \| P30041 \| \| P78509 \| \| Q16363 \| \| P21796 \| \| P01031 \| \| P62266 \| \| P61163 \| \| P00736 \| \| Q00341 \| \| Q8NBM4 \| \| P36542 \| \| Q9UK41 \| \| Q6YHK3 \| \| P62913 \| \| Q00610 \| \| Q9Y639 \| \| Q14019 \| \| Q96TA1 \| \| O75452 \| \| Q9P2R7 \| \| P11021 \| \| P15559 \| \| P63010 \| \| P02649 \| \| P49747 \| \| P61088 \| \| Q9Y2A7 \| \| P22760 \| \| O15484 \| \| O75891 \| \| Q9NPH3 \| \| P12081 \| \| P32969 \| \| O95197 \| \| P50148 \| \| Q16610 \| \| P83731 \| \| Q9HDC9 \| \| P22307 \| \| P20337 \| \| Q9Y625 \| \| P42766 \| \| O75131 \| \| P25789 \| \| P10768 \| \| P31327 \| \| P10916 \| \| P62081 \| \| O43491 \| \| P05787 \| \| P27797 \| \| P46783 \| \| P40925 \| \| P30512 \| \| P00352 \| \| P13591 \| \| O00468 \| \| O75144 \| \| Q99715 \| \| P56199 \| \| P05091 \| \| Q14108 \| \| Q06830 \| \| Q03135 \| \| P11413 \| \| Q9GZX5 \| \| P13726 \| \| P07942 \| \| P11047 \| \| P62191 \| \| P55285 \| \| P07099 \| \| Q9Y6C2 \| \| Q93088 \| \| P11498 \| \| P11233 \| \| O43242 \| \| Q14112 \| \| Q12907 \| \| P11279 \| \| P35268 \| \| P22692 \| \| P03951 \| \| Q9Y3B3 \| \| P09110 \| \| P00966 \| \| P33121 \| \| Q00266 \| \| P61927 \| \| P36871 \| \| Q9Y5U8 \| \| Q8N6Y2 \| \| Q9Y617 \| \| P49638 \| \| Q53S08 \| \| P23284 \| \| P07585 \| \| P46940 \| \| Q9BXJ4 \| \| P22033 \| \| P08243 \| \| Q9P0V3 \| \| P52272 \| \| P07237 \| \| O00483 \| \| P13533 \| \| Q9Y5C1 \| \| P24752 \| \| P23526 \| \| P62333 \| \| O75489 \| \| Q9ULV4 \| \| P40926 \| \| P68402 \| \| Q9UKK3 \| \| O00264 \| \| P28161 \| \| P22626 \| \| P48723 \| \| P24821 \| \| P09467 \| \| Q96QK1 \| \| Q96AG4 \| \| O76081 \| \| Q7Z5G4 \| \| P53007 \| \| Q8N474 \| \| O75695 \| \| P06576 \| \| P00491 \| \| P48506 \| \| P23381 \| \| P49720 \| \| Q16851 \| \| O15145 \| \| P25705 \| \| O43181 \| \| P60059 \| \| P28838 \| \| Q04446 \| \| Q15008 \| \| Q9NRV9 \| \| P30042 \| \| Q8NE62 \| \| P55287 \| \| P67812 \| \| Q9HB63 \| \| P51665 \| \| Q6P9B6 \| \| Q96BJ3 \| \| Q9BSR8 \| \| O00186 \| \| Q8NBS9 \| \| P12235 \| \| P11169 \| \| Q5TDH0 \| \| Q7L1Q6 \| \| Q9NUI1 \| \| Q6DD88 \| \| P11177 \| \| O43772 \| \| P84103 \| \| Q9GZP0 \| \| Q92542 \| \| Q00325 \| \| P60900 \| \| P17987 \| \| Q86TX2 \| \| P28288 \| \| P58546 \| \| Q76LX8 \| \| P21912 \| \| P05997 \| \| P26927 \| \| P16671 \| \| P56556 \| \| P05362 \| \| P07947 \| \| P54819 \| \| Q15916 \| \| P36957 \| \| P42357 \| \| Q16891 \| \| Q15233 \| \| P09543 \| \| O95498 \| \| P30086 \| \| Q9H8H3 \| \| O95831 \| \| O95563 \| \| P07358 \| \| P10632 \| \| Q14117 \| \| P62280 \| \| Q86UX7 \| \| P32929 \| \| P31930 \| \| O14495 \| \| P28066 \| \| P49748 \| \| P10646 \| \| Q9H1K4 \| \| Q14254 \| \| Q15286 \| \| P00439 \| \| P17661 \| \| P40939 \| \| Q16134 \| \| P54868 \| \| P02786 \| \| O60488 \| \| P49207 \| \| Q6NXR4 \| \| P12107 \| \| Q9UI17 \| \| P16152 \| \| P61604 \| \| Q9UL12 \| \| P08574 \| \| Q99816 \| \| Q8N884 \| \| P16035 \| \| Q9Y277 \| \| P51648 \| \| Q02338 \| \| O14949 \| \| P62328 \| \| P05026 \| \| O15144 \| \| Q15493 \| \| P45379 \| \| Q9Y623 \| \| P35609 \| \| Q15084 \| \| P62750 \| \| P19429 \| \| P21399 \| \| P30048 \| \| Q13813 \| \| Q9UHF3 \| \| P09493 \| \| P08590 \| \| P22695 \| \| Q07065 \| \| P47756 \| \| P39656 \| \| Q02978 \| \| Q9UJS0 \| \| P16435 \| \| P51659 \| \| Q4LDE5 \| \| P11766 \| \| Q5VTU8 \| \| P05106 \| \| P14854 \| \| O75367 \| \| P04745 \| \| P05783 \| \| P23141 \| \| Q14749 \| \| P15954 \| \| P0C0L4 \| \| P35232 \| \| O75390 \| \| P14550 \| \| P04844 \| \| P99999 \| \| P10643 \| \| P04843 \| \| P48047 \| \| P19224 \| \| P0C7P4 \| \| P12883 \| \| Q13162 \| \| P48735 \| \| P24539 \| \| P20618 \| \| Q05707 \| \| P32754 \| \| P38117 \| \| P13804 \| \| P16401 \| \| P07305 \| \| P24593 \| \| P10620 \| \| Q04828 \| \| Q96IX5 \| \| P00326 \| \| P05141 \| \| P00480 \| \| P46439 \| \| Q7RTV2 \| \| P00167 \| \| P07451 \| | \| INS \| \| --- \| \| C1RL \| \| BTD \| \| CA1 \| \| ERN1 \| \| ABI3BP \| \| FETUB \| \| TMEM198 \| \| APOC1 \| \| HBD \| \| C4B \| \| PLAUR \| \| PROCR \| \| GAPDHS \| \| SET \| \| CSF1R \| \| HBB \| \| GP1BA \| \| IGHG2 \| \| A1BG \| \| PTGFRN \| \| VPS4B \| \| ARF5 \| \| FUCA1 \| \| AMBP \| \| CACYBP \| \| ALDOC \| \| APOA2 \| \| TMEM119 \| \| HP \| \| LPXN \| \| HIST1H1C \| \| SAA4 \| \| SERPINA3 \| \| ORM2 \| \| TTR \| \| RTCB \| \| AFM \| \| UBE2M \| \| CD14 \| \| CP \| \| PI16 \| \| LRG1 \| \| GPLD1 \| \| BCHE \| \| EPRS \| \| PRAF2 \| \| PFN2 \| \| LCAT \| \| SERPINA1 \| \| IGKC \| \| AZGP1 \| \| GGH \| \| VTA1 \| \| HPX \| \| PGLYRP2 \| \| EHD4 \| \| CPN2 \| \| NNMT \| \| PLXDC2 \| \| SPON2 \| \| ATRN \| \| SRPX2 \| \| HPR \| \| IGHG1 \| \| IGHM \| \| F5 \| \| TSPAN9 \| \| FUCA2 \| \| GOT1 \| \| ORM1 \| \| APOC2 \| \| ACHE \| \| SLC1A4 \| \| CCT7 \| \| AGT \| \| SLC39A14 \| \| COL6A2 \| \| CTBS \| \| RPS8 \| \| SERPING1 \| \| CPN1 \| \| GALK1 \| \| COL6A1 \| \| NUTF2 \| \| CSAD \| \| CAPN1 \| \| TUFM \| \| RPL7A \| \| PCOLCE \| \| SLC3A2 \| \| RPLP1 \| \| CAT \| \| RPS15A \| \| HMGB1 \| \| AHSG \| \| SDCBP \| \| TPT1 \| \| ITGA3 \| \| RPS9 \| \| PRDX5 \| \| S100A16 \| \| CASR \| \| RPL4 \| \| S100A6 \| \| LGALS3BP \| \| MYADM \| \| NRAS \| \| BASP1 \| \| SELENBP1 \| \| RSU1 \| \| MRGPRF \| \| COX5B \| \| CLEC3B \| \| RPL10A \| \| PKM \| \| GNB1 \| \| ACTR2 \| \| ITGA4 \| \| TGFBI \| \| HBA1 \| \| PROS1 \| \| TUBB4B \| \| DECR1 \| \| ALCAM \| \| NUCB1 \| \| CACNA2D1 \| \| PGK1 \| \| RPS25 \| \| BMP1 \| \| RAP1B \| \| LGALS1 \| \| RAB5C \| \| EVA1B \| \| ATP1A1 \| \| RPL27 \| \| RACK1 \| \| ENG \| \| NRP1 \| \| GLIPR2 \| \| GNAI2 \| \| ACTG1 \| \| CCT4 \| \| ANXA6 \| \| ECHS1 \| \| UBA1 \| \| PEPD \| \| S100A11 \| \| PPIA \| \| RPS3A \| \| EEF2 \| \| BSG \| \| RAP1A \| \| IGFBP2 \| \| TUBA1A \| \| CMPK1 \| \| VCAN \| \| CD81 \| \| GNPNAT1 \| \| APOB \| \| APOD \| \| CD59 \| \| MMP14 \| \| IGFALS \| \| RPS3 \| \| A2M \| \| EEF1A1 \| \| ITIH4 \| \| RPS26 \| \| FAP \| \| PCBP2 \| \| DPP4 \| \| PHB2 \| \| ANXA2 \| \| RPL7 \| \| ENO1 \| \| HSP90AB1 \| \| RPS2 \| \| ANXA11 \| \| CTSD \| \| IGLL5 \| \| RPL5 \| \| ITGB1 \| \| MMP2 \| \| PSMA3 \| \| NT5E \| \| CD9 \| \| ARPC4 \| \| ITGA5 \| \| VNN1 \| \| AHNAK \| \| TPI1 \| \| ANXA1 \| \| HSP90AA1 \| \| TIMP1 \| \| TUBB1 \| \| COL4A2 \| \| RPL35A \| \| MOGS \| \| YWHAB \| \| PFN1 \| \| SOD2 \| \| WDR1 \| \| CCT3 \| \| HNRNPU \| \| SERPIND1 \| \| YWHAE \| \| MCAM \| \| RPLP0 \| \| EHD2 \| \| PDGFRB \| \| MYO1C \| \| HNRNPK \| \| PLEK \| \| ACTA2 \| \| PLP2 \| \| HSP90AB4P \| \| CALM3 \| \| RPS4X \| \| PVR \| \| RPL21 \| \| JUP \| \| RPSA \| \| RFTN1 \| \| VCP \| \| F2 \| \| MVP \| \| FMNL3 \| \| HSPB1 \| \| UBA52 \| \| EDIL3 \| \| CLIC1 \| \| RPL6 \| \| PZP \| \| YWHAQ \| \| CDH2 \| \| CCT2 \| \| UBE2V1 \| \| B2M \| \| EHD3 \| \| C3 \| \| VAMP5 \| \| DSTN \| \| SLC38A2 \| \| ANXA7 \| \| ATP2B4 \| \| SNAP23 \| \| ITGA2 \| \| PDCD6IP \| \| GC \| \| KRAS \| \| CTNNA1 \| \| CHMP4A \| \| PLSCR3 \| \| NCL \| \| RPL12 \| \| MSN \| \| AFP \| \| ALDOA \| \| RAC1 \| \| CYP2D7 \| \| PLXNB2 \| \| PYGL \| \| SUCLG2 \| \| DNAJC5 \| \| VIM \| \| MYOF \| \| PTK7 \| \| ATP1B3 \| \| PXDN \| \| PRSS23 \| \| HADHB \| \| YWHAG \| \| PDIA3 \| \| CD248 \| \| BRD9 \| \| APLP2 \| \| TAGLN \| \| ART4 \| \| EGFR \| \| MYL6 \| \| ATP11B \| \| NECTIN2 \| \| ABCA10 \| \| UGDH \| \| GOT2 \| \| RAB23 \| \| SPARC \| \| ANXA5 \| \| COL1A1 \| \| RPL18 \| \| HSPG2 \| \| FN1 \| \| COL3A1 \| \| RCN1 \| \| ATIC \| \| YWHAZ \| \| FLNC \| \| RPL15 \| \| LOXL2 \| \| ALDH4A1 \| \| GNAS \| \| HSPA8 \| \| LDHA \| \| DNAH8 \| \| NME2 \| \| C9 \| \| MYH9 \| \| SEPT2 \| \| COL18A1 \| \| HGFAC \| \| CAPZA1 \| \| LGALS3 \| \| EIF3L \| \| RTN4 \| \| ASL \| \| NBEA \| \| HSP90B1 \| \| ITIH1 \| \| SLC2A1 \| \| SERPINC1 \| \| SERPINF1 \| \| ITIH3 \| \| SRSF8 \| \| RAB1A \| \| LDHB \| \| RPL17 \| \| IGF2R \| \| HIST1H3A \| \| RPL3 \| \| THBS1 \| \| APOA4 \| \| RAB7A \| \| ARF6 \| \| COPG1 \| \| PDCD6 \| \| RPS20 \| \| ITIH2 \| \| PRDX2 \| \| UBE2L3 \| \| CDCP1 \| \| LTF \| \| RAB11A \| \| TMED10 \| \| CPNE1 \| \| ACTN1 \| \| COL1A2 \| \| ACLY \| \| F13A1 \| \| PSMA7 \| \| HRNR \| \| EZR \| \| TRA2B \| \| CD44 \| \| SURF4 \| \| RRAS2 \| \| RDX \| \| COL5A1 \| \| HIST1H4A \| \| RPL14 \| \| HSD17B6 \| \| CALU \| \| GPX3 \| \| F10 \| \| HSPD1 \| \| S100A10 \| \| TLN1 \| \| GLUD1 \| \| RAB2A \| \| RPL8 \| \| CTNND1 \| \| SHMT2 \| \| ARHGDIA \| \| BANF1 \| \| CACHD1 \| \| FLNA \| \| FARP1 \| \| EFEMP1 \| \| PLG \| \| CD276 \| \| RPL19 \| \| CD47 \| \| DOCK10 \| \| CSPG4 \| \| FEN1 \| \| RPS24 \| \| S100A13 \| \| NID1 \| \| QSOX1 \| \| ACO2 \| \| MFGE8 \| \| C1S \| \| YWHAH \| \| RAB14 \| \| NDUFA11 \| \| PCBP1 \| \| VCL \| \| HSPA9 \| \| SERPINH1 \| \| PRDX6 \| \| RELN \| \| LAMA4 \| \| VDAC1 \| \| C5 \| \| RPS23 \| \| ACTR1A \| \| C1R \| \| HDLBP \| \| UBAC2 \| \| ATP5F1C \| \| VPS28 \| \| CD109 \| \| RPL11 \| \| CLTC \| \| NPTN \| \| COTL1 \| \| FAM129B \| \| RDH16 \| \| SUCLA2 \| \| HSPA5 \| \| NQO1 \| \| AP2B1 \| \| APOE \| \| COMP \| \| UBE2N \| \| NCKAP1 \| \| AADAC \| \| CAPN5 \| \| ALDH1L1 \| \| IL1RAP \| \| HARS \| \| RPL9 \| \| RTN3 \| \| GNAQ \| \| ECM1 \| \| RPL24 \| \| APMAP \| \| SCP2 \| \| RAB3B \| \| GPC6 \| \| RPL35 \| \| CPNE3 \| \| PSMA4 \| \| ESD \| \| CPS1 \| \| MYL2 \| \| RPS7 \| \| EPB41L2 \| \| KRT8 \| \| CALR \| \| RPS10 \| \| MDH1 \| \| HLA-A \| \| ALDH1A1 \| \| NCAM1 \| \| AGRN \| \| ICOSLG \| \| COL12A1 \| \| ITGA1 \| \| ALDH2 \| \| SCARB2 \| \| PRDX1 \| \| CAV1 \| \| G6PD \| \| ZNF350 \| \| F3 \| \| LAMB1 \| \| LAMC1 \| \| PSMC1 \| \| CDH6 \| \| EPHX1 \| \| EMILIN1 \| \| BHMT \| \| PC \| \| RALA \| \| PSMD3 \| \| NID2 \| \| LMAN2 \| \| LAMP1 \| \| RPL22 \| \| IGFBP4 \| \| F11 \| \| TMED7 \| \| ACAA1 \| \| ASS1 \| \| ACSL1 \| \| MAT1A \| \| RPL37 \| \| PGM1 \| \| MPC1 \| \| LRRC17 \| \| PSAT1 \| \| TTPA \| \| RAB6D \| \| PPIB \| \| DCN \| \| IQGAP1 \| \| C1QTNF3 \| \| MUT \| \| ASNS \| \| SH3BP4 \| \| HNRNPM \| \| P4HB \| \| NDUFA4 \| \| MYH6 \| \| ANGPTL3 \| \| ACAT1 \| \| AHCY \| \| PSMC6 \| \| NDUFS3 \| \| CORO1C \| \| MDH2 \| \| PAFAH1B2 \| \| PARP4 \| \| PGRMC1 \| \| GSTM2 \| \| HNRNPA2B1 \| \| HSPA13 \| \| TNC \| \| FBP1 \| \| VPS35 \| \| LRRC59 \| \| RGS20 \| \| GOLGA7 \| \| SLC25A1 \| \| SFRP1 \| \| RP2 \| \| ATP5F1B \| \| PNP \| \| GCLC \| \| WARS \| \| PSMB3 \| \| UGP2 \| \| ARPC3 \| \| ATP5F1A \| \| NDUFS4 \| \| SEC61G \| \| LAP3 \| \| GBE1 \| \| PSMD6 \| \| HEBP1 \| \| C21orf33 \| \| CHDH \| \| CDH11 \| \| SEC11A \| \| NTN4 \| \| PSMD7 \| \| TLDC1 \| \| AIDA \| \| YIPF4 \| \| STXBP3 \| \| TXNDC5 \| \| SLC25A4 \| \| SLC2A3 \| \| DDI2 \| \| BZW1 \| \| DECR2 \| \| ATL3 \| \| PDHB \| \| SLC25A20 \| \| SRSF3 \| \| PDGFD \| \| NCSTN \| \| SLC25A3 \| \| PSMA6 \| \| TCP1 \| \| ACOT1 \| \| ABCD3 \| \| MTPN \| \| ADAMTS13 \| \| SDHB \| \| COL5A2 \| \| MST1 \| \| CD36 \| \| NDUFA6 \| \| ICAM1 \| \| YES1 \| \| AK2 \| \| ZBTB6 \| \| DLST \| \| HAL \| \| IMMT \| \| NONO \| \| CNP \| \| VNN2 \| \| PEBP1 \| \| METTL7A \| \| AIFM1 \| \| MPC2 \| \| C8B \| \| CYP2C8 \| \| DPYS \| \| RPS11 \| \| FERMT3 \| \| CTH \| \| UQCRC1 \| \| PLPP3 \| \| PSMA5 \| \| ACADVL \| \| TFPI \| \| SLC25A18 \| \| FLOT2 \| \| RAB35 \| \| PAH \| \| DES \| \| HADHA \| \| ETFDH \| \| HMGCS2 \| \| TFRC \| \| ACSL4 \| \| RPL34 \| \| TTI2 \| \| COL11A1 \| \| DMGDH \| \| CBR1 \| \| HSPE1 \| \| SARDH \| \| CYC1 \| \| TSG101 \| \| CGAS \| \| TIMP2 \| \| VDAC3 \| \| ALDH3A2 \| \| BDH1 \| \| UQCRQ \| \| TMSB4X \| \| ATP1B1 \| \| ARPC2 \| \| RGN \| \| TNNT2 \| \| MYH4 \| \| ACTN2 \| \| PDIA6 \| \| RPL23A \| \| TNNI3 \| \| ACO1 \| \| PRDX3 \| \| SPTAN1 \| \| NAT8B \| \| TPM1 \| \| MYL3 \| \| UQCRC2 \| \| CKAP4 \| \| CAPZB \| \| DDOST \| \| SLC25A11 \| \| SLC25A13 \| \| POR \| \| HSD17B4 \| \| SVEP1 \| \| ADH5 \| \| ATP5EP2 \| \| ITGB3 \| \| COX6B1 \| \| H2AFY \| \| AMY1A \| \| KRT18 \| \| CES1 \| \| GNMT \| \| COX7C \| \| C4A \| \| PHB \| \| CS \| \| AKR1A1 \| \| RPN2 \| \| CYCS \| \| C7 \| \| RPN1 \| \| ATP5O \| \| UGT1A6 \| \| UQCRFS1P1 \| \| MYH7 \| \| PRDX4 \| \| IDH2 \| \| ATP5F1 \| \| PSMB1 \| \| COL14A1 \| \| HPD \| \| ETFB \| \| ETFA \| \| HIST1H1B \| \| H1F0 \| \| IGFBP5 \| \| MGST1 \| \| AKR1C1 \| \| USMG5 \| \| ADH1C \| \| SLC25A5 \| \| OTC \| \| GSTM5 \| \| GSTA5 \| \| CYB5A \| \| CA3 \| | \| 36752.6911 \| \| --- \| \| 20955.0373 \| \| 8441.8935 \| \| 6258.2418 \| \| 5476.0863 \| \| 4268.6758 \| \| 2535.4952 \| \| 1828.3017 \| \| 1587.1361 \| \| 828.2586 \| \| 690.1120 \| \| 586.4130 \| \| 502.0182 \| \| 438.6467 \| \| 415.4453 \| \| 329.6882 \| \| 315.4754 \| \| 272.5189 \| \| 256.1820 \| \| 233.0649 \| \| 186.0209 \| \| 163.8793 \| \| 163.1180 \| \| 152.2331 \| \| 151.1660 \| \| 143.2159 \| \| 133.3726 \| \| 131.6063 \| \| 130.4991 \| \| 111.2255 \| \| 91.3675 \| \| 67.8078 \| \| 61.2260 \| \| 51.4930 \| \| 46.6485 \| \| 32.3949 \| \| 28.8474 \| \| 26.1149 \| \| 25.9969 \| \| 25.2397 \| \| 22.1388 \| \| 20.6784 \| \| 17.8901 \| \| 16.1956 \| \| 15.7252 \| \| 15.6986 \| \| 15.6986 \| \| 14.6554 \| \| 14.1825 \| \| 13.6324 \| \| 10.8000 \| \| 10.7952 \| \| 9.5267 \| \| 7.9639 \| \| 7.6489 \| \| 7.5220 \| \| 7.4209 \| \| 7.3302 \| \| 6.9380 \| \| 6.9012 \| \| 6.6549 \| \| 6.3406 \| \| 6.0202 \| \| 5.8450 \| \| 5.4681 \| \| 5.0974 \| \| 5.0652 \| \| 4.6934 \| \| 4.5126 \| \| 4.2923 \| \| 4.2239 \| \| 3.9934 \| \| 3.7554 \| \| 3.6200 \| \| 3.5893 \| \| 3.2948 \| \| 3.2431 \| \| 3.0262 \| \| 2.9411 \| \| 2.5573 \| \| 2.3997 \| \| 2.3267 \| \| 2.1499 \| \| 0.4998 \| \| 0.4931 \| \| 0.4927 \| \| 0.4923 \| \| 0.4796 \| \| 0.4660 \| \| 0.4626 \| \| 0.4577 \| \| 0.4545 \| \| 0.4519 \| \| 0.4415 \| \| 0.4369 \| \| 0.4302 \| \| 0.4252 \| \| 0.4226 \| \| 0.4194 \| \| 0.4173 \| \| 0.4054 \| \| 0.3912 \| \| 0.3855 \| \| 0.3846 \| \| 0.3819 \| \| 0.3733 \| \| 0.3701 \| \| 0.3698 \| \| 0.3610 \| \| 0.3567 \| \| 0.3542 \| \| 0.3480 \| \| 0.3392 \| \| 0.3383 \| \| 0.3364 \| \| 0.3363 \| \| 0.3339 \| \| 0.3253 \| \| 0.3241 \| \| 0.3230 \| \| 0.3229 \| \| 0.3205 \| \| 0.3196 \| \| 0.3195 \| \| 0.3187 \| \| 0.3167 \| \| 0.3167 \| \| 0.3159 \| \| 0.3146 \| \| 0.3096 \| \| 0.3093 \| \| 0.3086 \| \| 0.3076 \| \| 0.3039 \| \| 0.3025 \| \| 0.3000 \| \| 0.2949 \| \| 0.2938 \| \| 0.2931 \| \| 0.2913 \| \| 0.2831 \| \| 0.2817 \| \| 0.2798 \| \| 0.2796 \| \| 0.2795 \| \| 0.2790 \| \| 0.2719 \| \| 0.2700 \| \| 0.2673 \| \| 0.2666 \| \| 0.2653 \| \| 0.2644 \| \| 0.2623 \| \| 0.2618 \| \| 0.2604 \| \| 0.2600 \| \| 0.2570 \| \| 0.2569 \| \| 0.2543 \| \| 0.2534 \| \| 0.2531 \| \| 0.2520 \| \| 0.2510 \| \| 0.2480 \| \| 0.2471 \| \| 0.2452 \| \| 0.2448 \| \| 0.2444 \| \| 0.2442 \| \| 0.2421 \| \| 0.2407 \| \| 0.2400 \| \| 0.2397 \| \| 0.2359 \| \| 0.2357 \| \| 0.2342 \| \| 0.2321 \| \| 0.2255 \| \| 0.2237 \| \| 0.2210 \| \| 0.2208 \| \| 0.2205 \| \| 0.2203 \| \| 0.2200 \| \| 0.2188 \| \| 0.2178 \| \| 0.2139 \| \| 0.2136 \| \| 0.2135 \| \| 0.2129 \| \| 0.2126 \| \| 0.2091 \| \| 0.2085 \| \| 0.2082 \| \| 0.2078 \| \| 0.2076 \| \| 0.2068 \| \| 0.2058 \| \| 0.2048 \| \| 0.2045 \| \| 0.2045 \| \| 0.2033 \| \| 0.2026 \| \| 0.2023 \| \| 0.2014 \| \| 0.2010 \| \| 0.1991 \| \| 0.1990 \| \| 0.1983 \| \| 0.1980 \| \| 0.1977 \| \| 0.1949 \| \| 0.1920 \| \| 0.1920 \| \| 0.1911 \| \| 0.1904 \| \| 0.1884 \| \| 0.1884 \| \| 0.1872 \| \| 0.1872 \| \| 0.1864 \| \| 0.1849 \| \| 0.1844 \| \| 0.1840 \| \| 0.1836 \| \| 0.1778 \| \| 0.1778 \| \| 0.1765 \| \| 0.1740 \| \| 0.1723 \| \| 0.1720 \| \| 0.1711 \| \| 0.1709 \| \| 0.1704 \| \| 0.1682 \| \| 0.1677 \| \| 0.1676 \| \| 0.1647 \| \| 0.1645 \| \| 0.1641 \| \| 0.1637 \| \| 0.1637 \| \| 0.1635 \| \| 0.1621 \| \| 0.1601 \| \| 0.1600 \| \| 0.1598 \| \| 0.1587 \| \| 0.1583 \| \| 0.1574 \| \| 0.1561 \| \| 0.1549 \| \| 0.1534 \| \| 0.1533 \| \| 0.1509 \| \| 0.1502 \| \| 0.1489 \| \| 0.1482 \| \| 0.1462 \| \| 0.1457 \| \| 0.1457 \| \| 0.1449 \| \| 0.1445 \| \| 0.1430 \| \| 0.1426 \| \| 0.1425 \| \| 0.1424 \| \| 0.1416 \| \| 0.1410 \| \| 0.1405 \| \| 0.1398 \| \| 0.1392 \| \| 0.1388 \| \| 0.1384 \| \| 0.1377 \| \| 0.1366 \| \| 0.1364 \| \| 0.1361 \| \| 0.1354 \| \| 0.1348 \| \| 0.1346 \| \| 0.1337 \| \| 0.1331 \| \| 0.1327 \| \| 0.1323 \| \| 0.1313 \| \| 0.1308 \| \| 0.1308 \| \| 0.1293 \| \| 0.1293 \| \| 0.1276 \| \| 0.1273 \| \| 0.1265 \| \| 0.1253 \| \| 0.1252 \| \| 0.1247 \| \| 0.1236 \| \| 0.1233 \| \| 0.1219 \| \| 0.1205 \| \| 0.1196 \| \| 0.1183 \| \| 0.1182 \| \| 0.1178 \| \| 0.1168 \| \| 0.1158 \| \| 0.1155 \| \| 0.1155 \| \| 0.1153 \| \| 0.1151 \| \| 0.1134 \| \| 0.1122 \| \| 0.1119 \| \| 0.1115 \| \| 0.1105 \| \| 0.1096 \| \| 0.1094 \| \| 0.1069 \| \| 0.1060 \| \| 0.1058 \| \| 0.1057 \| \| 0.1047 \| \| 0.1045 \| \| 0.1040 \| \| 0.1039 \| \| 0.1036 \| \| 0.1036 \| \| 0.1030 \| \| 0.1018 \| \| 0.1010 \| \| 0.0998 \| \| 0.0991 \| \| 0.0974 \| \| 0.0974 \| \| 0.0960 \| \| 0.0959 \| \| 0.0957 \| \| 0.0951 \| \| 0.0950 \| \| 0.0942 \| \| 0.0938 \| \| 0.0933 \| \| 0.0932 \| \| 0.0931 \| \| 0.0930 \| \| 0.0927 \| \| 0.0924 \| \| 0.0903 \| \| 0.0886 \| \| 0.0881 \| \| 0.0872 \| \| 0.0870 \| \| 0.0868 \| \| 0.0861 \| \| 0.0851 \| \| 0.0849 \| \| 0.0833 \| \| 0.0815 \| \| 0.0811 \| \| 0.0808 \| \| 0.0808 \| \| 0.0807 \| \| 0.0803 \| \| 0.0799 \| \| 0.0798 \| \| 0.0776 \| \| 0.0771 \| \| 0.0771 \| \| 0.0761 \| \| 0.0759 \| \| 0.0757 \| \| 0.0753 \| \| 0.0752 \| \| 0.0749 \| \| 0.0748 \| \| 0.0748 \| \| 0.0747 \| \| 0.0742 \| \| 0.0740 \| \| 0.0738 \| \| 0.0737 \| \| 0.0730 \| \| 0.0726 \| \| 0.0720 \| \| 0.0715 \| \| 0.0712 \| \| 0.0700 \| \| 0.0695 \| \| 0.0694 \| \| 0.0692 \| \| 0.0686 \| \| 0.0678 \| \| 0.0635 \| \| 0.0634 \| \| 0.0629 \| \| 0.0626 \| \| 0.0620 \| \| 0.0620 \| \| 0.0616 \| \| 0.0609 \| \| 0.0601 \| \| 0.0596 \| \| 0.0595 \| \| 0.0585 \| \| 0.0585 \| \| 0.0579 \| \| 0.0575 \| \| 0.0570 \| \| 0.0566 \| \| 0.0566 \| \| 0.0552 \| \| 0.0543 \| \| 0.0540 \| \| 0.0538 \| \| 0.0534 \| \| 0.0534 \| \| 0.0529 \| \| 0.0524 \| \| 0.0522 \| \| 0.0509 \| \| 0.0506 \| \| 0.0494 \| \| 0.0487 \| \| 0.0484 \| \| 0.0480 \| \| 0.0479 \| \| 0.0469 \| \| 0.0468 \| \| 0.0452 \| \| 0.0441 \| \| 0.0438 \| \| 0.0435 \| \| 0.0428 \| \| 0.0423 \| \| 0.0415 \| \| 0.0408 \| \| 0.0398 \| \| 0.0382 \| \| 0.0376 \| \| 0.0376 \| \| 0.0369 \| \| 0.0368 \| \| 0.0336 \| \| 0.0333 \| \| 0.0327 \| \| 0.0323 \| \| 0.0318 \| \| 0.0298 \| \| 0.0296 \| \| 0.0295 \| \| 0.0294 \| \| 0.0292 \| \| 0.0287 \| \| 0.0285 \| \| 0.0281 \| \| 0.0280 \| \| 0.0280 \| \| 0.0274 \| \| 0.0249 \| \| 0.0245 \| \| 0.0243 \| \| 0.0241 \| \| 0.0241 \| \| 0.0237 \| \| 0.0226 \| \| 0.0224 \| \| 0.0218 \| \| 0.0200 \| \| 0.0196 \| \| 0.0191 \| \| 0.0186 \| \| 0.0178 \| \| 0.0177 \| \| 0.0176 \| \| 0.0176 \| \| 0.0167 \| \| 0.0167 \| \| 0.0166 \| \| 0.0163 \| \| 0.0162 \| \| 0.0153 \| \| 0.0149 \| \| 0.0148 \| \| 0.0135 \| \| 0.0131 \| \| 0.0131 \| \| 0.0127 \| \| 0.0122 \| \| 0.0121 \| \| 0.0121 \| \| 0.0112 \| \| 0.0111 \| \| 0.0111 \| \| 0.0108 \| \| 0.0101 \| \| 0.0100 \| \| 0.0093 \| \| 0.0092 \| \| 0.0090 \| \| 0.0076 \| \| 0.0073 \| \| 0.0070 \| \| 0.0068 \| \| 0.0068 \| \| 0.0064 \| \| 0.0064 \| \| 0.0063 \| \| 0.0060 \| \| 0.0058 \| \| 0.0057 \| \| 0.0056 \| \| 0.0056 \| \| 0.0054 \| \| 0.0053 \| \| 0.0050 \| \| 0.0050 \| \| 0.0050 \| \| 0.0049 \| \| 0.0049 \| \| 0.0048 \| \| 0.0048 \| \| 0.0047 \| \| 0.0046 \| \| 0.0045 \| \| 0.0045 \| \| 0.0044 \| \| 0.0041 \| \| 0.0041 \| \| 0.0040 \| \| 0.0040 \| \| 0.0039 \| \| 0.0039 \| \| 0.0039 \| \| 0.0038 \| \| 0.0038 \| \| 0.0037 \| \| 0.0037 \| \| 0.0037 \| \| 0.0036 \| \| 0.0035 \| \| 0.0034 \| \| 0.0034 \| \| 0.0033 \| \| 0.0033 \| \| 0.0030 \| \| 0.0030 \| \| 0.0029 \| \| 0.0029 \| \| 0.0029 \| \| 0.0029 \| \| 0.0028 \| \| 0.0026 \| \| 0.0025 \| \| 0.0024 \| \| 0.0024 \| \| 0.0024 \| \| 0.0024 \| \| 0.0023 \| \| 0.0023 \| \| 0.0022 \| \| 0.0022 \| \| 0.0022 \| \| 0.0020 \| \| 0.0019 \| \| 0.0019 \| \| 0.0019 \| \| 0.0018 \| \| 0.0018 \| \| 0.0018 \| \| 0.0017 \| \| 0.0017 \| \| 0.0017 \| \| 0.0017 \| \| 0.0017 \| \| 0.0017 \| \| 0.0016 \| \| 0.0016 \| \| 0.0016 \| \| 0.0016 \| \| 0.0016 \| \| 0.0015 \| \| 0.0014 \| \| 0.0014 \| \| 0.0014 \| \| 0.0013 \| \| 0.0013 \| \| 0.0013 \| \| 0.0012 \| \| 0.0012 \| \| 0.0012 \| \| 0.0011 \| \| 0.0011 \| \| 0.0011 \| \| 0.0011 \| \| 0.0010 \| \| 0.0010 \| \| 0.0010 \| \| 0.0010 \| \| 0.0010 \| \| 0.0010 \| \| 0.0010 \| \| 0.0009 \| \| 0.0009 \| \| 0.0009 \| \| 0.0009 \| \| 0.0009 \| \| 0.0009 \| \| 0.0009 \| \| 0.0009 \| \| 0.0008 \| \| 0.0008 \| \| 0.0008 \| \| 0.0008 \| \| 0.0007 \| \| 0.0007 \| \| 0.0007 \| \| 0.0007 \| \| 0.0007 \| \| 0.0007 \| \| 0.0007 \| \| 0.0006 \| \| 0.0006 \| \| 0.0006 \| \| 0.0006 \| \| 0.0006 \| \| 0.0006 \| \| 0.0006 \| \| 0.0006 \| \| 0.0006 \| \| 0.0006 \| \| 0.0006 \| \| 0.0006 \| \| 0.0006 \| \| 0.0005 \| \| 0.0005 \| \| 0.0005 \| \| 0.0005 \| \| 0.0005 \| \| 0.0005 \| \| 0.0004 \| \| 0.0004 \| \| 0.0004 \| \| 0.0004 \| \| 0.0004 \| \| 0.0004 \| \| 0.0003 \| \| 0.0003 \| \| 0.0003 \| \| 0.0003 \| \| 0.0003 \| \| 0.0003 \| \| 0.0003 \| \| 0.0003 \| \| 0.0003 \| \| 0.0002 \| \| 0.0002 \| \| 0.0002 \| \| 0.0002 \| \| 0.0002 \| \| 0.0002 \| \| 0.0002 \| \| 0.0002 \| \| 0.0002 \| \| 0.0002 \| \| 0.0002 \| \| 0.0002 \| \| 0.0002 \| \| 0.0002 \| \| 0.0002 \| \| 0.0002 \| \| 0.0002 \| \| 0.0002 \| \| 0.0002 \| \| 0.0002 \| \| 0.0002 \| \| 0.0002 \| \| 0.0002 \| \| 0.0001 \| \| 0.0001 \| \| 0.0001 \| \| 0.0001 \| \| 0.0001 \| \| 0.0001 \| \| 0.0001 \| \| 0.0001 \| \| 0.0001 \| \| 0.0001 \| \| 0.0001 \| \| 0.0001 \| \| 0.0001 \| \| 0.0000 \| \| 0.0000 \| \| 0.0000 \| | \| 0.0105 \| \| --- \| \| 0.0011 \| \| 0.0010 \| \| 0.0015 \| \| 0.0169 \| \| 0.0009 \| \| 0.0009 \| \| 0.0182 \| \| 0.0069 \| \| 0.0483 \| \| 0.0008 \| \| 0.0111 \| \| 0.0389 \| \| 0.0090 \| \| 0.0018 \| \| 0.0082 \| \| 0.0019 \| \| 0.0097 \| \| 0.0014 \| \| 0.0018 \| \| 0.0004 \| \| 0.0277 \| \| 0.0024 \| \| 0.0218 \| \| 0.0111 \| \| 0.0301 \| \| 0.0105 \| \| 0.0018 \| \| 0.0028 \| \| 0.0016 \| \| 0.0361 \| \| 0.0025 \| \| 0.0097 \| \| 0.0013 \| \| 0.0001 \| \| 0.0008 \| \| 0.0036 \| \| 0.0038 \| \| 0.0003 \| \| 0.0191 \| \| 0.0004 \| \| 0.0002 \| \| 0.0000 \| \| 0.0009 \| \| 0.0004 \| \| 0.0258 \| \| 0.0258 \| \| 0.0032 \| \| 0.0000 \| \| 0.0060 \| \| 0.0054 \| \| 0.0058 \| \| 0.0113 \| \| 0.0044 \| \| 0.0002 \| \| 0.0009 \| \| 0.0002 \| \| 0.0048 \| \| 0.0164 \| \| 0.0020 \| \| 0.0026 \| \| 0.0000 \| \| 0.0217 \| \| 0.0217 \| \| 0.0011 \| \| 0.0140 \| \| 0.0410 \| \| 0.0085 \| \| 0.0180 \| \| 0.0171 \| \| 0.0091 \| \| 0.0290 \| \| 0.0179 \| \| 0.0332 \| \| 0.0109 \| \| 0.0034 \| \| 0.0256 \| \| 0.0474 \| \| 0.0043 \| \| 0.0098 \| \| 0.0002 \| \| 0.0258 \| \| 0.0097 \| \| 0.0065 \| \| 0.0036 \| \| 0.0124 \| \| 0.0104 \| \| 0.0076 \| \| 0.0216 \| \| 0.0024 \| \| 0.0060 \| \| 0.0075 \| \| 0.0345 \| \| 0.0059 \| \| 0.0301 \| \| 0.0340 \| \| 0.0252 \| \| 0.0102 \| \| 0.0019 \| \| 0.0015 \| \| 0.0053 \| \| 0.0089 \| \| 0.0308 \| \| 0.0113 \| \| 0.0280 \| \| 0.0341 \| \| 0.0033 \| \| 0.0159 \| \| 0.0015 \| \| 0.0010 \| \| 0.0024 \| \| 0.0291 \| \| 0.0125 \| \| 0.0082 \| \| 0.0469 \| \| 0.0003 \| \| 0.0499 \| \| 0.0283 \| \| 0.0101 \| \| 0.0001 \| \| 0.0071 \| \| 0.0167 \| \| 0.0001 \| \| 0.0150 \| \| 0.0233 \| \| 0.0108 \| \| 0.0152 \| \| 0.0048 \| \| 0.0019 \| \| 0.0244 \| \| 0.0295 \| \| 0.0273 \| \| 0.0056 \| \| 0.0068 \| \| 0.0047 \| \| 0.0227 \| \| 0.0242 \| \| 0.0174 \| \| 0.0183 \| \| 0.0086 \| \| 0.0014 \| \| 0.0052 \| \| 0.0008 \| \| 0.0015 \| \| 0.0442 \| \| 0.0077 \| \| 0.0018 \| \| 0.0005 \| \| 0.0157 \| \| 0.0101 \| \| 0.0217 \| \| 0.0000 \| \| 0.0392 \| \| 0.0246 \| \| 0.0036 \| \| 0.0171 \| \| 0.0113 \| \| 0.0070 \| \| 0.0080 \| \| 0.0051 \| \| 0.0210 \| \| 0.0013 \| \| 0.0407 \| \| 0.0124 \| \| 0.0113 \| \| 0.0008 \| \| 0.0017 \| \| 0.0010 \| \| 0.0239 \| \| 0.0220 \| \| 0.0071 \| \| 0.0000 \| \| 0.0118 \| \| 0.0037 \| \| 0.0001 \| \| 0.0081 \| \| 0.0000 \| \| 0.0109 \| \| 0.0319 \| \| 0.0041 \| \| 0.0028 \| \| 0.0071 \| \| 0.0019 \| \| 0.0155 \| \| 0.0034 \| \| 0.0080 \| \| 0.0242 \| \| 0.0259 \| \| 0.0243 \| \| 0.0298 \| \| 0.0314 \| \| 0.0043 \| \| 0.0047 \| \| 0.0297 \| \| 0.0017 \| \| 0.0169 \| \| 0.0083 \| \| 0.0145 \| \| 0.0496 \| \| 0.0462 \| \| 0.0093 \| \| 0.0232 \| \| 0.0010 \| \| 0.0385 \| \| 0.0364 \| \| 0.0012 \| \| 0.0234 \| \| 0.0000 \| \| 0.0142 \| \| 0.0215 \| \| 0.0041 \| \| 0.0224 \| \| 0.0003 \| \| 0.0299 \| \| 0.0049 \| \| 0.0305 \| \| 0.0435 \| \| 0.0185 \| \| 0.0491 \| \| 0.0132 \| \| 0.0029 \| \| 0.0448 \| \| 0.0000 \| \| 0.0185 \| \| 0.0005 \| \| 0.0000 \| \| 0.0079 \| \| 0.0201 \| \| 0.0005 \| \| 0.0121 \| \| 0.0009 \| \| 0.0043 \| \| 0.0002 \| \| 0.0179 \| \| 0.0123 \| \| 0.0272 \| \| 0.0405 \| \| 0.0324 \| \| 0.0060 \| \| 0.0134 \| \| 0.0002 \| \| 0.0324 \| \| 0.0342 \| \| 0.0000 \| \| 0.0438 \| \| 0.0122 \| \| 0.0208 \| \| 0.0000 \| \| 0.0075 \| \| 0.0218 \| \| 0.0470 \| \| 0.0034 \| \| 0.0244 \| \| 0.0209 \| \| 0.0000 \| \| 0.0326 \| \| 0.0018 \| \| 0.0166 \| \| 0.0189 \| \| 0.0260 \| \| 0.0052 \| \| 0.0404 \| \| 0.0149 \| \| 0.0085 \| \| 0.0491 \| \| 0.0000 \| \| 0.0125 \| \| 0.0287 \| \| 0.0039 \| \| 0.0192 \| \| 0.0121 \| \| 0.0118 \| \| 0.0293 \| \| 0.0154 \| \| 0.0448 \| \| 0.0174 \| \| 0.0080 \| \| 0.0192 \| \| 0.0369 \| \| 0.0015 \| \| 0.0058 \| \| 0.0122 \| \| 0.0095 \| \| 0.0215 \| \| 0.0001 \| \| 0.0039 \| \| 0.0246 \| \| 0.0112 \| \| 0.0100 \| \| 0.0185 \| \| 0.0066 \| \| 0.0050 \| \| 0.0021 \| \| 0.0055 \| \| 0.0417 \| \| 0.0182 \| \| 0.0039 \| \| 0.0143 \| \| 0.0339 \| \| 0.0186 \| \| 0.0061 \| \| 0.0051 \| \| 0.0057 \| \| 0.0084 \| \| 0.0003 \| \| 0.0027 \| \| 0.0007 \| \| 0.0207 \| \| 0.0286 \| \| 0.0021 \| \| 0.0445 \| \| 0.0095 \| \| 0.0124 \| \| 0.0112 \| \| 0.0069 \| \| 0.0468 \| \| 0.0253 \| \| 0.0194 \| \| 0.0035 \| \| 0.0009 \| \| 0.0005 \| \| 0.0172 \| \| 0.0381 \| \| 0.0235 \| \| 0.0033 \| \| 0.0073 \| \| 0.0113 \| \| 0.0227 \| \| 0.0362 \| \| 0.0316 \| \| 0.0071 \| \| 0.0234 \| \| 0.0000 \| \| 0.0084 \| \| 0.0019 \| \| 0.0003 \| \| 0.0099 \| \| 0.0050 \| \| 0.0037 \| \| 0.0146 \| \| 0.0318 \| \| 0.0202 \| \| 0.0095 \| \| 0.0012 \| \| 0.0055 \| \| 0.0157 \| \| 0.0096 \| \| 0.0154 \| \| 0.0008 \| \| 0.0026 \| \| 0.0177 \| \| 0.0258 \| \| 0.0324 \| \| 0.0041 \| \| 0.0270 \| \| 0.0130 \| \| 0.0003 \| \| 0.0118 \| \| 0.0132 \| \| 0.0042 \| \| 0.0010 \| \| 0.0353 \| \| 0.0003 \| \| 0.0013 \| \| 0.0041 \| \| 0.0156 \| \| 0.0042 \| \| 0.0135 \| \| 0.0121 \| \| 0.0088 \| \| 0.0232 \| \| 0.0126 \| \| 0.0175 \| \| 0.0043 \| \| 0.0101 \| \| 0.0334 \| \| 0.0108 \| \| 0.0047 \| \| 0.0111 \| \| 0.0113 \| \| 0.0000 \| \| 0.0244 \| \| 0.0234 \| \| 0.0021 \| \| 0.0019 \| \| 0.0271 \| \| 0.0126 \| \| 0.0083 \| \| 0.0108 \| \| 0.0188 \| \| 0.0073 \| \| 0.0076 \| \| 0.0470 \| \| 0.0011 \| \| 0.0120 \| \| 0.0003 \| \| 0.0084 \| \| 0.0109 \| \| 0.0085 \| \| 0.0028 \| \| 0.0429 \| \| 0.0052 \| \| 0.0010 \| \| 0.0019 \| \| 0.0003 \| \| 0.0142 \| \| 0.0000 \| \| 0.0004 \| \| 0.0057 \| \| 0.0081 \| \| 0.0001 \| \| 0.0000 \| \| 0.0003 \| \| 0.0067 \| \| 0.0051 \| \| 0.0008 \| \| 0.0472 \| \| 0.0002 \| \| 0.0073 \| \| 0.0046 \| \| 0.0293 \| \| 0.0427 \| \| 0.0045 \| \| 0.0053 \| \| 0.0074 \| \| 0.0470 \| \| 0.0246 \| \| 0.0141 \| \| 0.0084 \| \| 0.0000 \| \| 0.0410 \| \| 0.0009 \| \| 0.0061 \| \| 0.0323 \| \| 0.0059 \| \| 0.0072 \| \| 0.0079 \| \| 0.0077 \| \| 0.0076 \| \| 0.0001 \| \| 0.0001 \| \| 0.0002 \| \| 0.0047 \| \| 0.0192 \| \| 0.0024 \| \| 0.0078 \| \| 0.0067 \| \| 0.0108 \| \| 0.0037 \| \| 0.0170 \| \| 0.0151 \| \| 0.0198 \| \| 0.0229 \| \| 0.0018 \| \| 0.0229 \| \| 0.0075 \| \| 0.0317 \| \| 0.0147 \| \| 0.0020 \| \| 0.0086 \| \| 0.0314 \| \| 0.0005 \| \| 0.0299 \| \| 0.0282 \| \| 0.0104 \| \| 0.0018 \| \| 0.0023 \| \| 0.0001 \| \| 0.0023 \| \| 0.0147 \| \| 0.0002 \| \| 0.0026 \| \| 0.0264 \| \| 0.0064 \| \| 0.0039 \| \| 0.0368 \| \| 0.0044 \| \| 0.0016 \| \| 0.0007 \| \| 0.0376 \| \| 0.0114 \| \| 0.0124 \| \| 0.0185 \| \| 0.0069 \| \| 0.0012 \| \| 0.0009 \| \| 0.0072 \| \| 0.0077 \| \| 0.0070 \| \| 0.0114 \| \| 0.0031 \| \| 0.0163 \| \| 0.0182 \| \| 0.0132 \| \| 0.0058 \| \| 0.0193 \| \| 0.0110 \| \| 0.0301 \| \| 0.0263 \| \| 0.0259 \| \| 0.0186 \| \| 0.0260 \| \| 0.0074 \| \| 0.0066 \| \| 0.0144 \| \| 0.0277 \| \| 0.0138 \| \| 0.0027 \| \| 0.0465 \| \| 0.0114 \| \| 0.0184 \| \| 0.0031 \| \| 0.0189 \| \| 0.0267 \| \| 0.0007 \| \| 0.0001 \| \| 0.0044 \| \| 0.0017 \| \| 0.0016 \| \| 0.0175 \| \| 0.0201 \| \| 0.0027 \| \| 0.0413 \| \| 0.0093 \| \| 0.0067 \| \| 0.0057 \| \| 0.0272 \| \| 0.0049 \| \| 0.0052 \| \| 0.0398 \| \| 0.0352 \| \| 0.0340 \| \| 0.0303 \| \| 0.0065 \| \| 0.0049 \| \| 0.0283 \| \| 0.0017 \| \| 0.0414 \| \| 0.0013 \| \| 0.0051 \| \| 0.0028 \| \| 0.0415 \| \| 0.0097 \| \| 0.0329 \| \| 0.0330 \| \| 0.0044 \| \| 0.0457 \| \| 0.0212 \| \| 0.0071 \| \| 0.0085 \| \| 0.0240 \| \| 0.0058 \| \| 0.0040 \| \| 0.0026 \| \| 0.0211 \| \| 0.0288 \| \| 0.0159 \| \| 0.0030 \| \| 0.0084 \| \| 0.0463 \| \| 0.0408 \| \| 0.0284 \| \| 0.0010 \| \| 0.0117 \| \| 0.0245 \| \| 0.0233 \| \| 0.0357 \| \| 0.0007 \| \| 0.0321 \| \| 0.0086 \| \| 0.0077 \| \| 0.0487 \| \| 0.0257 \| \| 0.0215 \| \| 0.0114 \| \| 0.0203 \| \| 0.0125 \| \| 0.0242 \| \| 0.0042 \| \| 0.0239 \| \| 0.0443 \| \| 0.0061 \| \| 0.0332 \| \| 0.0453 \| \| 0.0189 \| \| 0.0405 \| \| 0.0377 \| \| 0.0051 \| \| 0.0146 \| \| 0.0405 \| \| 0.0044 \| \| 0.0054 \| \| 0.0261 \| \| 0.0070 \| \| 0.0189 \| \| 0.0224 \| \| 0.0410 \| \| 0.0023 \| \| 0.0163 \| \| 0.0028 \| \| 0.0465 \| \| 0.0174 \| \| 0.0082 \| \| 0.0053 \| \| 0.0246 \| \| 0.0066 \| \| 0.0047 \| \| 0.0141 \| \| 0.0023 \| \| 0.0375 \| \| 0.0103 \| \| 0.0109 \| \| 0.0144 \| \| 0.0256 \| \| 0.0198 \| \| 0.0044 \| \| 0.0146 \| \| 0.0461 \| \| 0.0061 \| \| 0.0209 \| \| 0.0375 \| \| 0.0019 \| \| 0.0112 \| \| 0.0498 \| \| 0.0200 \| \| 0.0183 \| \| 0.0070 \| \| 0.0256 \| \| 0.0021 \| \| 0.0192 \| \| 0.0250 \| \| 0.0159 \| \| 0.0419 \| \| 0.0099 \| \| 0.0099 \| \| 0.0030 \| \| 0.0182 \| \| 0.0152 \| \| 0.0140 \| \| 0.0171 \| \| 0.0011 \| \| 0.0112 \| \| 0.0100 \| \| 0.0011 \| \| 0.0021 \| \| 0.0053 \| \| 0.0028 \| \| 0.0183 \| \| 0.0037 \| \| 0.0061 \| \| 0.0092 \| \| 0.0092 \| \| 0.0036 \| \| 0.0013 \| \| 0.0149 \| \| 0.0252 \| \| 0.0261 \| \| 0.0383 \| \| 0.0051 \| \| 0.0010 \| \| 0.0270 \| \| 0.0181 \| \| 0.0057 \| \| 0.0099 \| \| 0.0088 \| \| 0.0040 \| \| 0.0036 \| \| 0.0000 \| \| 0.0124 \| \| 0.0352 \| \| 0.0041 \| \| 0.0049 \| \| 0.0039 \| \| 0.0140 \| \| 0.0087 \| \| 0.0159 \| \| 0.0125 \| \| 0.0013 \| \| 0.0193 \| \| 0.0272 \| \| 0.0071 \| \| 0.0202 \| \| 0.0033 \| \| 0.0202 \| \| 0.0099 \| \| 0.0118 \| \| 0.0232 \| \| 0.0023 \| \| 0.0067 \| \| 0.0236 \| \| 0.0134 \| \| 0.0062 \| \| 0.0076 \| |

*Data were analyzed by proteomic analysis.
