# Supplementary figures and images for: Mesenchymal Stem Cells-Derived Exosomes Alleviate Acute Lung Injury by Inhibiting Alveolar Macrophage Pyroptosis
Source: Stem Cells Transl Med. 2024 Feb 13;13(4):371–86. doi: 10.1093/stcltm/szad094 (PMC11016849; doi:10.1093/stcltm/szad094)

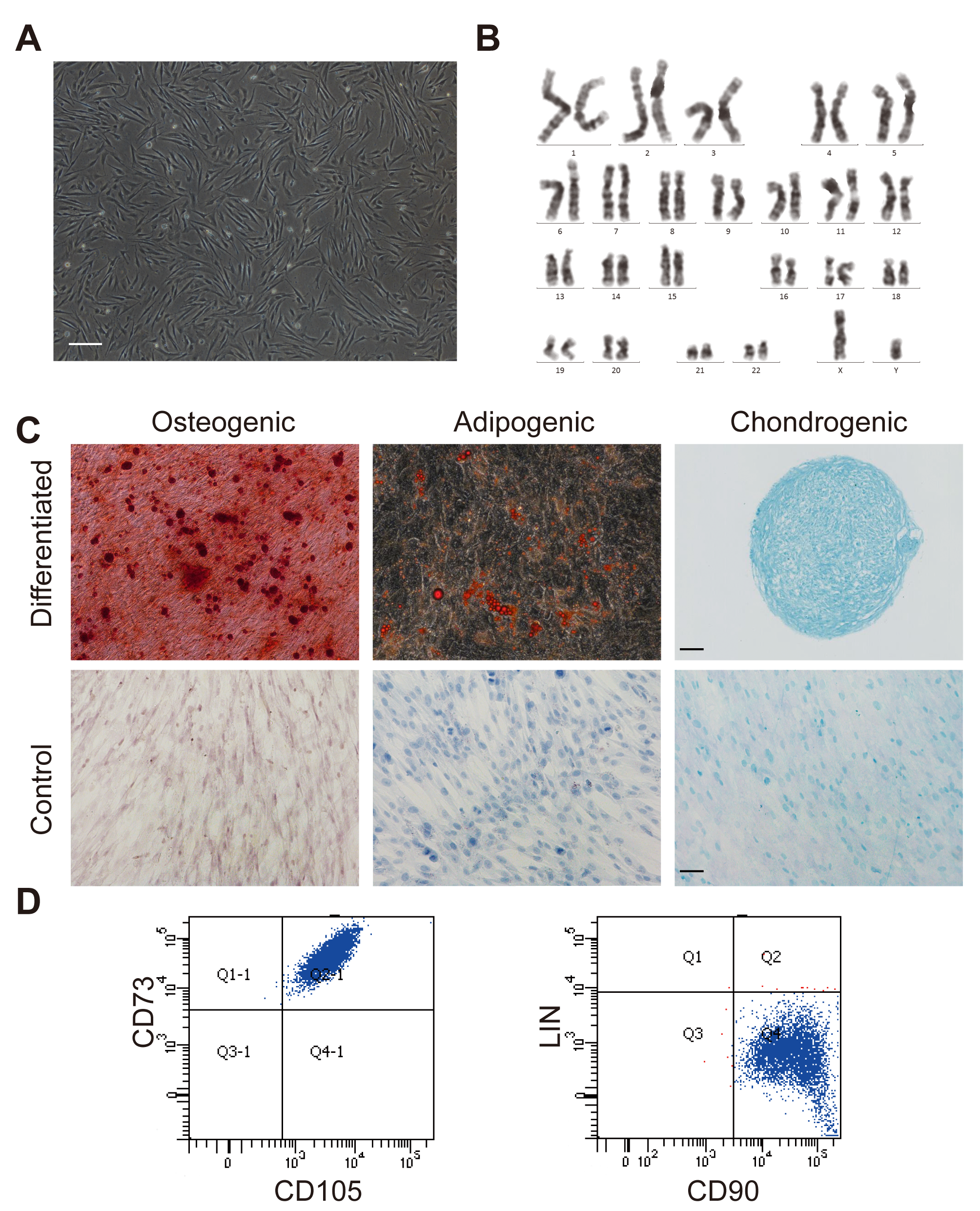

Supplement: szad094_suppl_Supplementary_Materials [file szad094_suppl_supplementary_materials.zip › szad094_suppl_Supplementary_Figures_S1.tif]

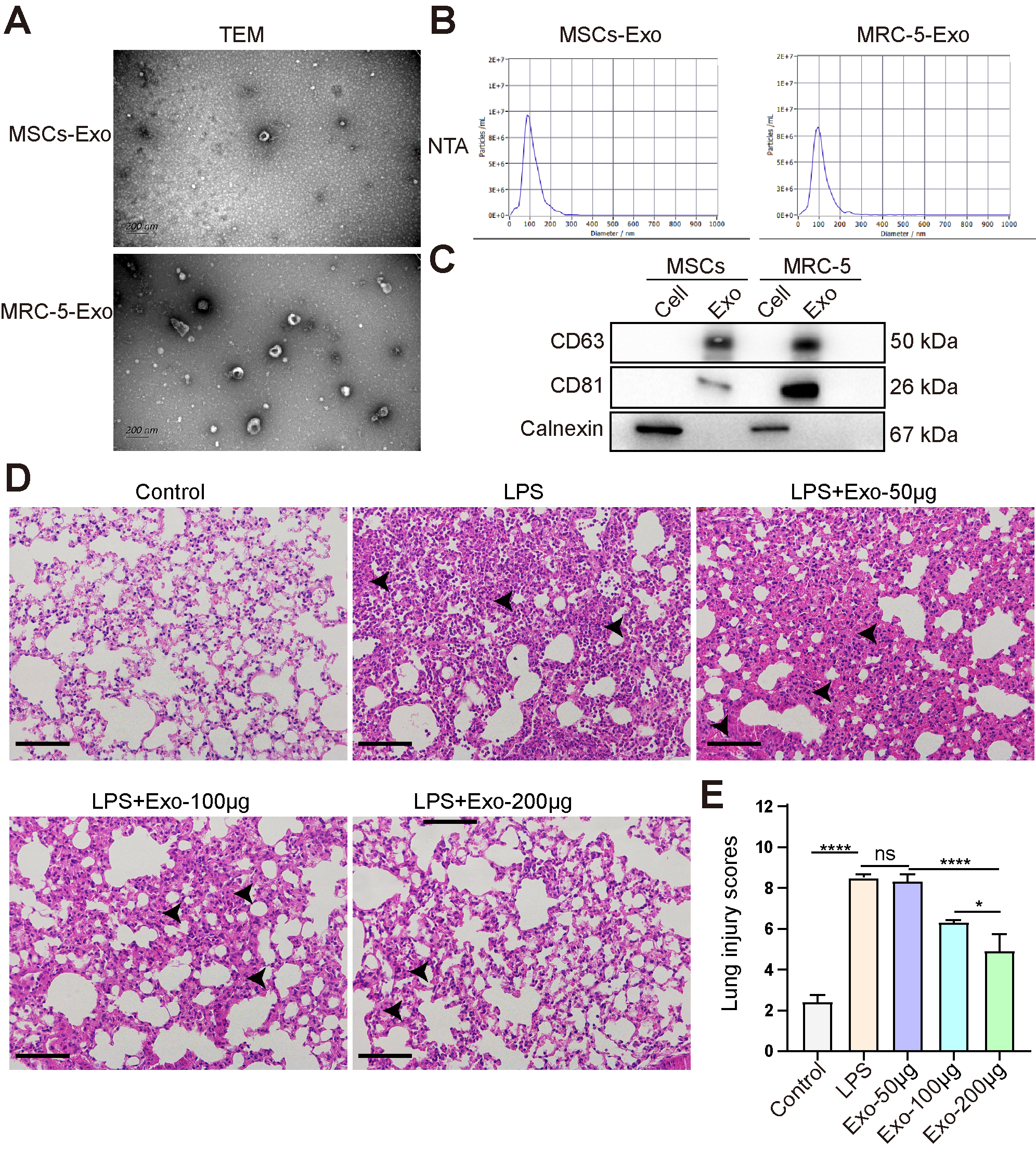

Supplement: szad094_suppl_Supplementary_Materials [file szad094_suppl_supplementary_materials.zip › szad094_suppl_Supplementary_Figures_S2.tif]

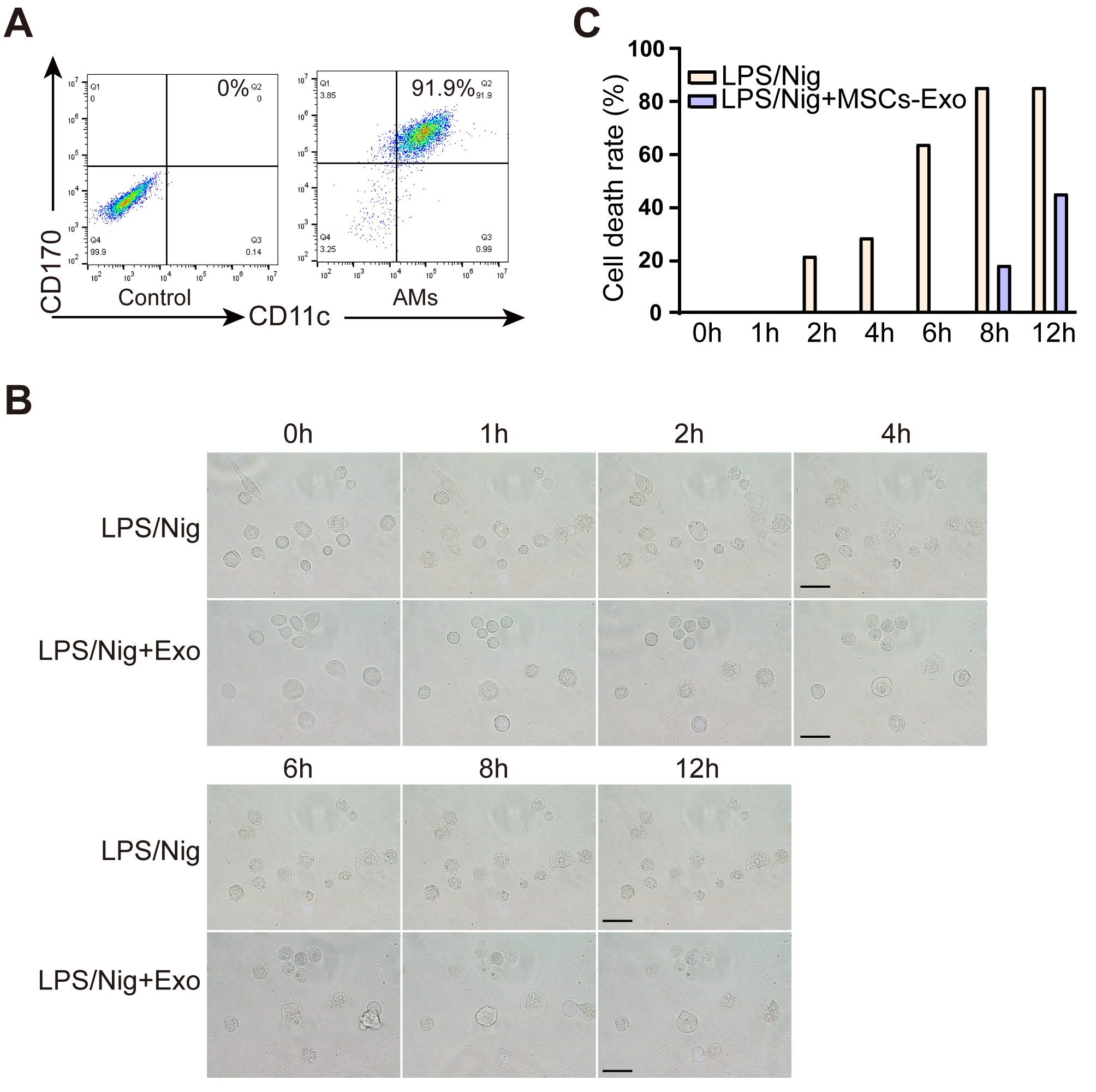

Supplement: szad094_suppl_Supplementary_Materials [file szad094_suppl_supplementary_materials.zip › szad094_suppl_Supplementary_Figures_S3.tif]

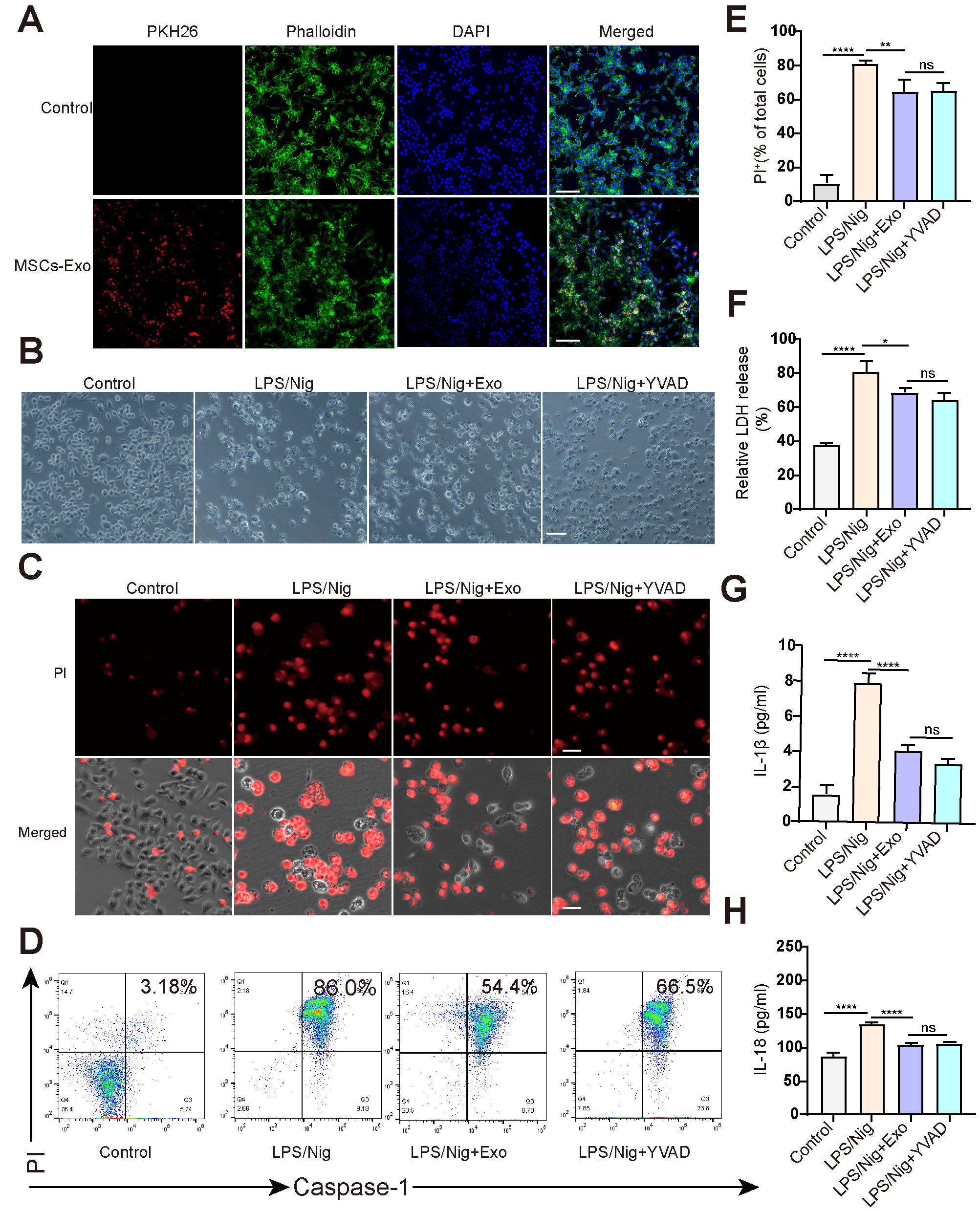

Supplement: szad094_suppl_Supplementary_Materials [file szad094_suppl_supplementary_materials.zip › szad094_suppl_Supplementary_Figures_S4.tif]

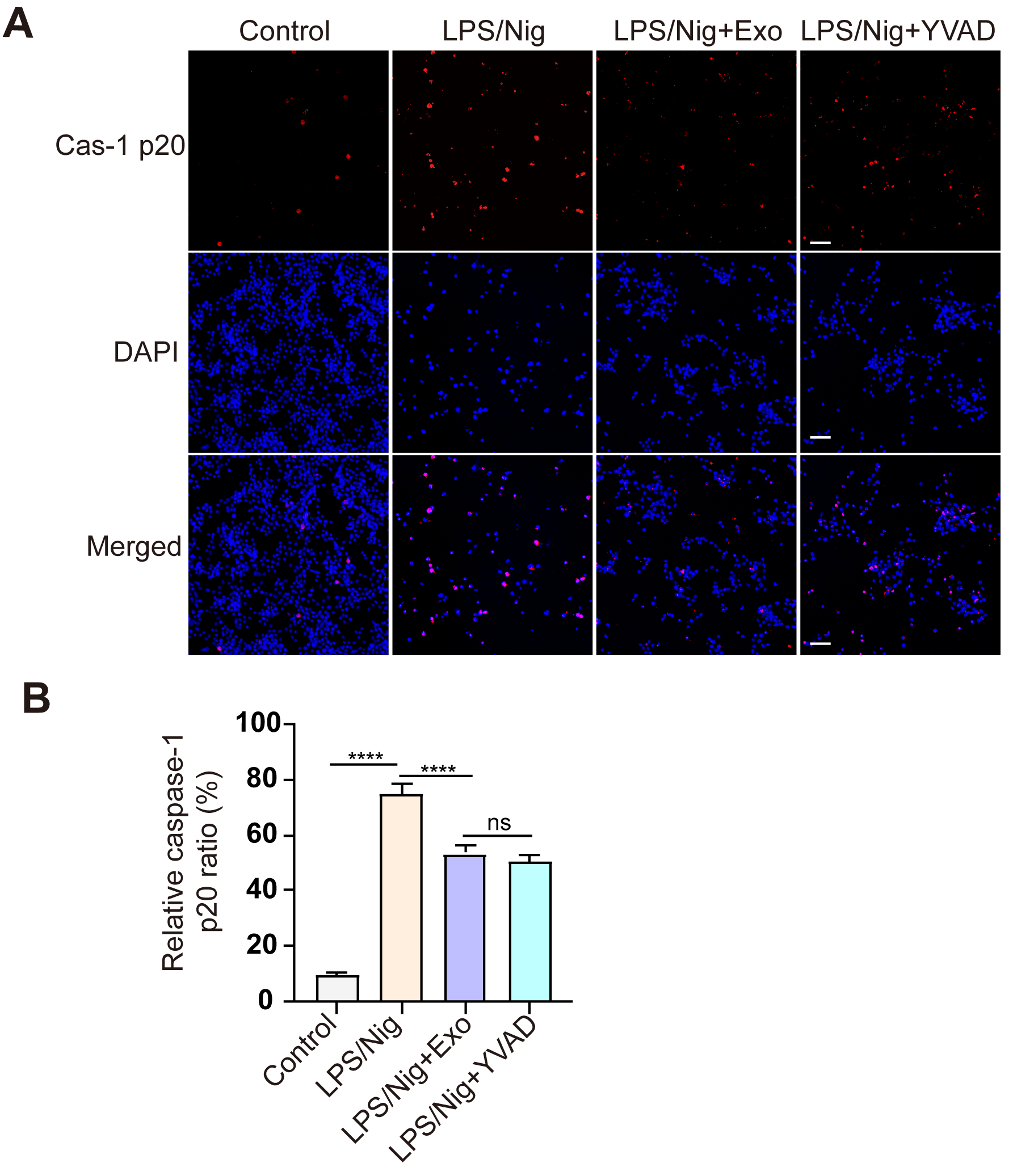

Supplement: szad094_suppl_Supplementary_Materials [file szad094_suppl_supplementary_materials.zip › szad094_suppl_Supplementary_Figures_S5.tif]

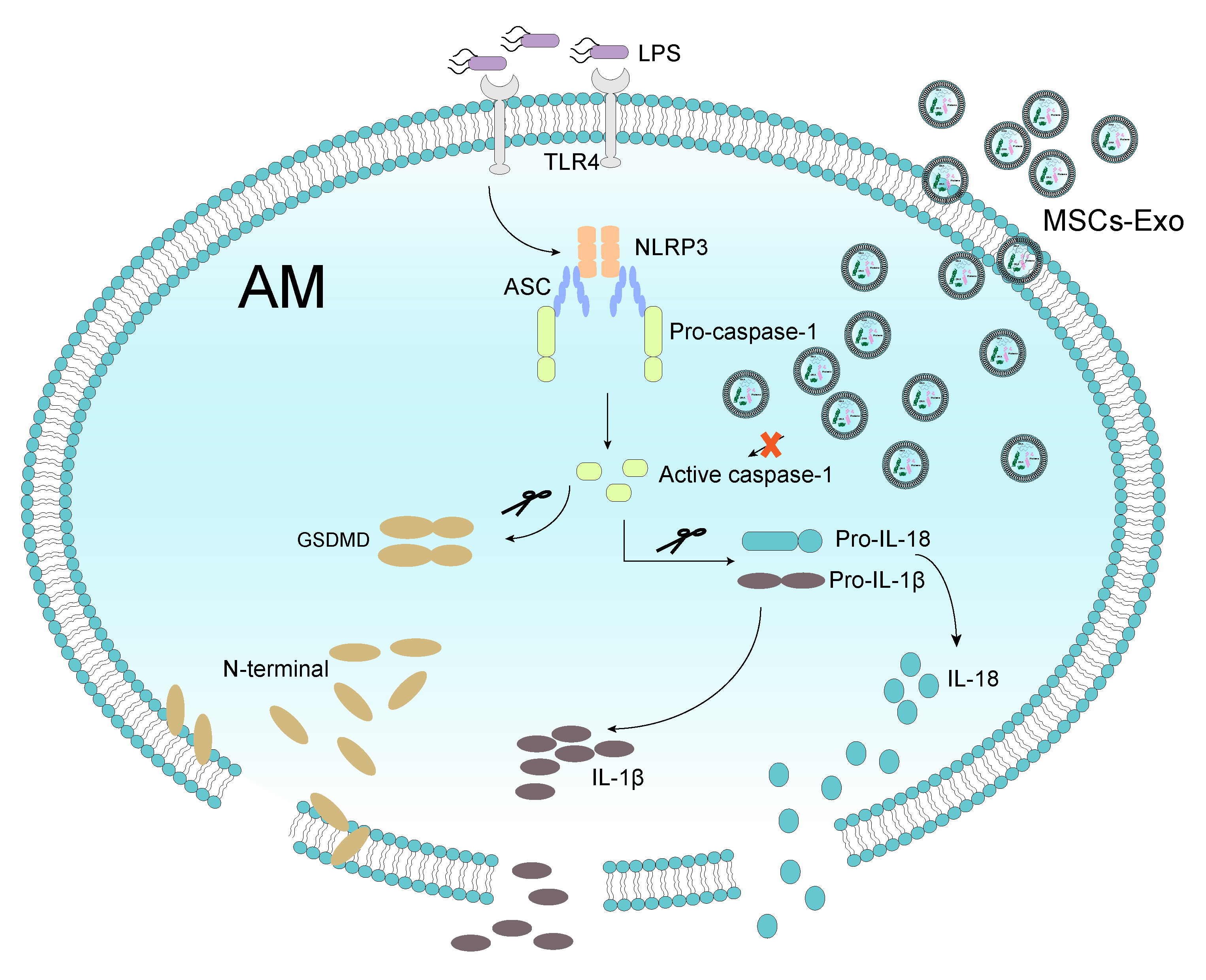

Supplement: szad094_suppl_Supplementary_Materials [file szad094_suppl_supplementary_materials.zip › szad094_suppl_Supplementary_Figures_S6.tif]
